# Direct estrogen receptor (ER) / HER family crosstalk mediating sensitivity to lumretuzumab and pertuzumab in ER+ breast cancer

**DOI:** 10.1371/journal.pone.0177331

**Published:** 2017-05-11

**Authors:** Denis Collins, Wolfgang Jacob, Juan Miguel Cejalvo, Maurizio Ceppi, Ian James, Max Hasmann, John Crown, Andrés Cervantes, Martin Weisser, Birgit Bossenmaier

**Affiliations:** 1 National Institute for Cellular Biotechnology, Dublin City University, Dublin, Ireland; 2 Roche Innovation Center Munich, Penzberg, Germany; 3 Department of Medical Oncology, Institute of Health Research INCLIVA, University of Valencia, Valencia, Spain; 4 A4P Consulting Ltd, Sandwich, United Kingdom; 5 Department of Medical Oncology, St. Vincent's University Hospital, Dublin, Ireland; Universita Campus Bio-Medico di Roma, ITALY

## Abstract

Bidirectional cross talk between members of the human epidermal growth factor family of receptors (HER) and the estrogen receptor (ER) is believed to underlie resistance mechanisms that develop in response to treatment with anti-HER agents and endocrine therapy. We investigated the interaction between HER2, HER3 and the ER *in vitro* using human embryonic kidney cells transfected with human HER2, HER3, and ERα. We also investigated the additive efficacy of combination regimens consisting of anti-HER3 (lumretuzumab), anti-HER2 (pertuzumab), and endocrine (fulvestrant) therapy *in vivo*. Our data show that both HER2 and HER3 can directly complex with the ER and can mediate phosphorylation of the ER. Phosphorylation of the ER was only observed in cells that expressed both HER2 and ERα or in heregulin-stimulated cells that expressed both HER3 and ERα. Using a mouse xenograft model of ER+/HER2-low (HER2 immunohistochemistry 1+ or 2+ without gene amplification) human breast cancer we show that the combination of lumretuzumab and pertuzumab is highly efficacious and induces long-lasting tumor regression *in vivo* and adding endocrine therapy (fulvestrant) to this combination further improved efficacy. In addition, a prolonged clinical response was observed with the combination of lumretuzumab and pertuzumab in a patient with ER+/HER2-low breast cancer who had failed endocrine therapy. These preclinical data confirm that direct cross talk exists between HER2/HER3 and ER which may explain the resistance mechanisms to endocrine therapy and monoclonal antibodies that target HER2 and HER3. Our data also indicate that the triplet of anti-HER2, anti-HER3, and endocrine therapy might be an efficacious combination for treating patients with ER+/HER2-low breast cancer, which is an area of significant unmet medical need.

## Introduction

Receptor tyrosine kinases such as the human epidermal growth factor receptor 1 (HER1/EGFR), HER2 (ERBB2), or HER3 (ERBB3) can act as primary oncogenic drivers when mutated [1–3]. *HER2* amplification is a marker of an aggressive tumor phenotype and before the advent of trastuzumab patients with HER2-positive (HER2+) breast cancer had a poor prognosis and shorter survival compared with those whose tumors did not overexpress HER2 [4–6]. Approximately 20–25% of breast cancer tumors are HER2+ and are now effectively treated with a combination of trastuzumab and pertuzumab added to standard chemotherapy [7,8], thereby transforming the treatment paradigm in this population of breast cancer.

A significant percentage of breast cancer cases have detectable HER2 expression without amplification of the *HER2* gene [9–11]; these cases can be termed ‘HER2-low’. This low level HER2 expression may favor the formation of HER2:HER3 or HER2:HER1 heterodimers over HER2:HER2 homodimers. The dominant heterodimer is HER2:HER3 –the formation of which is mediated by the binding of heregulin (HRG) to HER3 [12,13]. The HER2:HER3 heterodimer is the most potent oncogenic driver of all possible HER family dimers [14,15].

The hormone receptors for estrogen (ER) and progesterone (PR) are also widely used predictive biomarkers for classifying breast cancer and guiding therapeutic strategies [16]. Activation of the ER drives tumor growth in breast cancer and approximately 70% of all breast cancers are ER positive (ER+) [16,17]. Anti-hormonal agents that i) disrupt ER activity, such as tamoxifen, or ii) enzymatically block aromatase, such as letrozole or exemestane, or iii) selectively block and degrade ER, such as fulvestrant, are an essential part of current treatment strategies in adjuvant as well as advanced and metastatic disease [18].

Up to two-thirds of all HER2+ breast cancers also express hormone receptors [19–21]. There is evidence for crosstalk between HER2 and ER in this group demonstrated by preclinical and clinical data. The presence of ER and HER2 impacts on the efficacy of HER2-targeted therapies and HER2 status also impacts on response to endocrine therapy [22,23]. While adding HER2-targeted therapy to endocrine therapy significantly improves efficacy as compared to endocrine therapy alone in patients with advanced HER2+/ER+ disease [24,25], data from the neoadjuvant setting show that the response to anti-HER2 therapy is higher in patients with HER2+/ER- compared with HER2+/ER+ tumors [26,27].

Primary or secondary resistance to endocrine therapy is a major cause of treatment failure [28]. Several resistance mechanisms to endocrine therapy have been described, including alternative signaling through PI3K (phosphoinositide 3-kinase) and mTOR (mechanistic target of rapamycin) pathways, development of *ESR1* (estrogen receptor 1) mutations, and cell cycle checkpoint alterations [29]. A bidirectional crosstalk between ER and HER family receptors has also been hypothesized [30,31] as a resistance mechanism to endocrine therapy involving activation of HER2 and HER3 [32,33]. The HER2:HER3 heterodimer activates the associated PI3K/AKT and MAPK downstream signaling cascades but can also induce phosphorylation of the ER independent of estrogen [34,35] which may reduce the effectiveness of endocrine therapies [30]. Conversely, upregulation of the ER or its downstream products is believed to be a key escape pathway in the development of resistance to anti-HER2 therapy [36].

Despite several indirect descriptions of crosstalk between HER family members and the ER conferring drug resistance, no direct interaction between HER receptors and ER has been demonstrated. We investigated the interaction between HER2/HER3 and the ER in human cells transfected with both receptors. We also investigated the efficacy of the combination of lumretuzumab (an anti-HER3 antibody [37–39]) and pertuzumab (an anti-HER2 antibody [40]) with a selective estrogen receptor degrader (fulvestrant) in patient-derived mouse xenograft models of HER3+/HER2-low/ER+ breast cancer.

## Materials and methods

### Cell lines and therapeutic agents

Human ER+/HER2-low breast cancer cell lines (MCF-7 [#HTB-22], T47D [#HTB-133], ZR-75-1 [#CRL-1500], and MDA-MB-175 [#HTB-25]) and HEK 293 [#CRL-1573] human embryonic kidney cells were obtained from the American Type Culture Collection. This supplier routinely authenticates cells lines by karyotyping, short tandem repeat profiling, assessment of cell morphology, and species verification by isoenzymology. Upon receipt, cell lines were expanded and aliquots frozen. Cells were passaged for a maximum of 6 months after resuscitation; after 6 months cells were discarded and new aliquots of cells were resuscitated. Cells were cultured in RPMI 1640 or MEM Eagle medium (PAN Biotech GmbH, Aidenbach, Germany) containing 10% fetal calf serum (FCS; PAA Laboratories GmbH, Pasching, Austria) at 37°C in a water-saturated atmosphere containing 5% CO_2_.

The anti-HER3 antibody lumretuzumab, and the anti-HER2 antibodies pertuzumab (Perjeta^®^) and trastuzumab (Herceptin^®^) were obtained from Roche (Penzberg, Germany). The anti-estrogen fulvestrant (Faslodex^®^) was obtained from AstraZeneca.

### Inhibition of HER2 / HER3 signaling

Inhibition of HER2 / HER3 and the downstream MAPK and PI3K/AKT signaling pathways were examined in ER+/HER2-low/HER3+ MCF-7 cells stimulated with or without HRG (5 ng/mL for 10 minutes) *in vitro* and in tumor cells harvested from HBCx-19-bearing mice (see below) treated with lumretuzumab and pertuzumab.

Following cell lysis, standard SDS PAGE and Western blotting was used as described previously [39] to measure levels of HER3 (clone C-17, Santa Cruz Biotechnology, Heidelberg, Germany, #sc-285), phosphorylated HER3 (pHER3, clone 21D3 [Tyr1289], Cell Signaling Technologies, Danvers, USA, #4791), HER2 (Merck Millipore, Schaffhausen, Germany; #EP1045Y), pHER2 (Cell Signaling Technology, #2249), pMAPK (Cell Signaling Technology, #9106), AKT (Cell Signaling Technology, #9272), pAKT [Ser473] (Cell Signaling Technology, #4060), and phosphorylated src homology collagen (pSHC; Upstate Biotechnology, Lake Placid, USA, #07–206).

### HBCx-19 mouse xenograft model

All animal experiments were carried out in accordance with ethical and local authority guidelines. An ER+/HER2-low/HER3+ human breast cancer tumor fragment derived from a metastasis of a lobular carcinoma (HBCx-19) was obtained with informed consent from a patient treated at a cancer center. Tissue fragments were subcutaneously transplanted onto 5–10 female outbred athymic (*nu/nu*) donor mice (HSD:Athymic Nude-Foxn1^nu^; Harlan Laboratories, Gannat, France). Donor mice were sacrificed when tumors reached 1000–2000 mm^3^ in volume and tumors aseptically excised and dissected. Necrotic areas were removed and the remaining tumors were cut into fragments measuring approximately 20 mm^3^ and transferred in culture media before grafting. Mice were anaesthetized with 100 mg/kg ketamine hydrochloride and 10 mg/kg xylazine, the skin was sterilized with chlorhexidine solution, and incised at the level of the interscapular region. A 20 mm^3^ tumor fragment was placed in the subcutaneous tissue and the skin was closed with clips. All mice from the same experiment were implanted on the same day.

All animal experiments were conducted at the animal facility at XenTech (Evry, France) and were approved by the Direction des Services Vétérinaires, Ministère de l'Agriculture et de la Pêche, France (agreement No. B-91-228-107). Animals were delivered to the laboratory 7 days before the experiments during which time they were acclimatized to laboratory conditions. Mice were housed inside individually ventilated cages (IVC) under a light-dark cycle (14-hour circadian cycle of artificial light) and controlled room temperature and humidity. Food and water were provided *ad libitum*. Animals were controlled daily for clinical symptoms and detection of adverse effects. Mice received estrogen diluted in drinking water (ß-estradiol, 8.5 mg/L), from the date of tumor implant to the end of the study.

For investigation of lumretuzumab and pertuzumab, tumor-bearing animals were treated with vehicle (20 mM histidine monohydrochloride monohydrate, 140 mM NaCl, pH 6) or lumretuzumab (10 mg/kg intraperitoneally [i.p.]) or pertuzumab (15 mg/kg i.p. with a two-fold loading dose) as single agents or in combinations. For investigation of the additional benefit of adding fulvestrant to lumretuzumab and pertuzumab, the same animal model was used and was treated with vehicle, single-agent lumretuzumab (3 mg/kg i.p.), single-agent pertuzumab (3 mg/kg i.p.), single-agent fulvestrant (50 mg/kg intramuscularly [i.m.]) or with the reported combinations. Treatments were given weekly for six weeks or for up to 9 weeks starting at randomization when median tumor size was approximately 100–150 mm^3^. Tumor volume was measured using calipers every 3–4 days and the percentage tumor growth inhibition relative to control animals was calculated as described previously [37,39].

### HER2 / HER3 and ERα transfected cells

5 × 10^5^ HEK 293 cells in DMEM 10% FCS were seeded into six-well plates. Cells were transfected with human *ERα (ESR1)*, *HER2*, or *HER3* (alone or in combination) using 2 μg of each human cDNA or control vector (empty vector) and a standard FuGENE^®^ (Promega GmbH, Mannheim, Germany) protocol. Transfected cells were starved for 48 hours in DMEM supplemented with only 0.5% FCS, after which cells were either stimulated with HRG (5 ng/mL for 10 minutes) or left untreated. Cells were cultured in the absence of estrogen. Cells were lysed after 48 hours by a Triton X100 lysis buffer containing aprotinin 10 μL/mL, phenylmethylsulfonyl fluoride (PMSF) 10 μL/mL and orthovanadate 2 μL/mL.

### Direct HER2 / HER3 and ERα crosstalk in transfected cells

For assessment of HER2/ERα-complex formation, cell lysates were either immunoprecipitated with an anti-HER2 antibody (trastuzumab) and Western blotted using an anti-ERα antibody (Cell Signaling Technologies; #8644) or, immunoprecipitated with an anti-ERα antibody (Cell Signaling Technologies; #8644) and Western blotted using an anti-HER2 antibody (Merck Millipore, #EP1045Y). For assessment of HER3/ERα complex formation lysates were either immunoprecipitated with an anti-HER3 antibody (Roche; #Ab208) and Western blotted with an anti-ERα antibody (Cell Signaling Technologies; #8644), or immunoprecipitated with an anti-ERα antibody (Cell Signaling Technologies; #8644), and Western blotted with an anti-HER3 antibody (Roche; #Ab208). For assessment of total HER2, HER3, ERα and pERα levels, 20 μg of total cell lysate was separated by SDS PAGE and Western blotting performed according to standard methods using antibodies for pERα (Cell Signaling Technologies, #2511), ERα (Cell Signaling Technologies; #8644), HER2 (Merck Millipore; #EP1045Y) and HER3 (Roche #Ab208).

### Statistical methods

Differences in biomarker expression levels between the different treatment cohorts were examined using the Tukey-Kramer procedure which is a test procedure adjusting for multiple testing situations. The analysis was performed using the software JMP^®^ (Version 12.1.0, SAS Institute Inc., Cary, NC, 1989–2007). Differences in tumor growth inhibition between the different treatment groups was analyzed statistically using non-parametric methods due to the fact that the data showed asymmetrical behavior. Data were baseline corrected with the tumor volume at treatment start. Treatment-to-control ratios (TCR) were calculated with the upper and lower confidence intervals as described in S1 Statistical Analyses.

### Patient case study

The clinical efficacy of the combination of lumretuzumab and pertuzumab was described in a patient from a phase Ib, multicenter, open-label study (n = 35; clinicaltrials.gov identifier NCT01918254). The patient in question achieved a complete response (CR) under the trial conditions in Cohort 2 (lumretuzumab at 500 mg, pertuzumab at 840 mg for Cycle 1 and 420 mg for subsequent cycles; objective response rate– 30%) [41]. The study was conducted in accordance with the Declaration of Helsinki, current International Conference on Harmonisation of Technical Requirements for Registration of Pharmaceuticals for Human Use (ICH) guidelines, and all applicable regulatory and ethical requirements. The patient provided written informed consent before study-related procedures were performed. The study was approved by the Clinical Research Ethics Committee Parc de Salut MAR (Approval number: 2013/5076) on 07/05/2013. Tumors were assessed according to Response Evaluation Criteria in Solid Tumours (RECIST) version 1.1.

## Results

### Lumretuzumab and pertuzumab potently inhibit HER2/HER3 signaling *in vitro*

Pertuzumab was selected for investigation in combination with lumretuzumab as unlike trastuzumab (another anti-HER2 mAb) pertuzumab inhibits dimerization of HER2 with both HER3 and HER1 [8,42]. Therefore, we hypothesized that lumretuzumab and pertuzumab would provide comprehensive HER-family inhibition.

**Fig 1:**
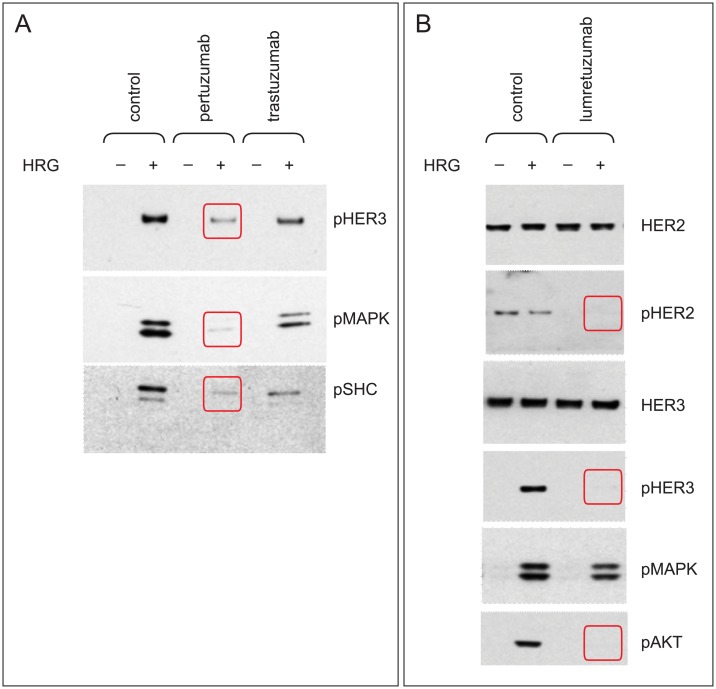
Lumretuzumab and pertuzumab both potently inhibit HER2/HER3 signaling. MC7-7 cells (3 × 10^5^ per six-well plate) were stimulated with or without HRG (5 ng/mL for 10 minutes) in vitro and treated with pertuzumab, trastuzumab, or lumretuzumab each at 10 μmol/L or vehicle control (PBS) for 1 hour, after which cell lysates were examined by Western blotting. Downregulation of pHER3, pMAPK, and pSHC was greater with pertuzumab than trastuzumab (**A**). Lumretuzumab monotherapy effectively inhibited HER2/HER3 signaling (**B**). All experiments were carried out in triplicate.

HER2 receptor dimerization in MCF-7 cells stimulated with HRG was more potently inhibited with single-agent pertuzumab than with single-agent trastuzumab, as evidenced by stronger inhibition of phosphorylation of HER3 and the downstream signaling molecules MAPK and SHC (Fig 1). Anti-HER3 treatment with single-agent lumretuzumab also potently inhibited HER2/HER3 signaling and virtually eliminated phosphorylation of HER2, HER3, and AKT in cells stimulated with HRG.

### The lumretuzumab plus pertuzumab combination induces complete and long-lasting tumor response in estrogen-dependent in vivo breast cancer models

**Fig 2:**
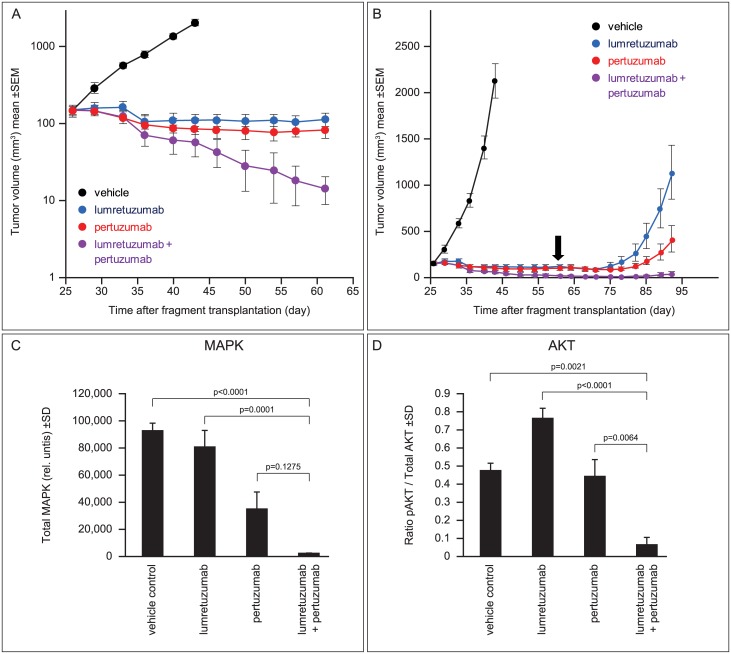
Lumretuzumab plus pertuzumab induces long-lasting tumor remission and inhibits HER2/HER3 signaling in estrogen-dependent breast cancer. (**A**) Outbred athymic (nu/nu) female mice (n = 10 per treatment group) bearing ER+/HER2-low/HER3+ human breast cancer xenografts (HBCx-19) were treated with single-agent lumretuzumab (10 mg/kg i.p.), single-agent pertuzumab (15 mg/kg i.p. with a two-fold loading dose), the combination of both antibodies, or with vehicle only (control). All treatments were given weekly beginning on Day 26 when median tumor size was 100–150 mm^3^ for 6 weeks (until Day 61). (**B**) Longer term follow-up of the mice depicted in Fig 1A showed that remissions in mice treated with lumretuzumab plus pertuzumab were long-lasting after the final dose of combination therapy on Day 61 (black arrow). Inhibition of MAPK (**C**) and AKT (**D**) in tumors harvested from mice on Day 94 (Day 40 for vehicle controls) was greatest with combination therapy.

To investigate the *in vivo* efficacy of lumretuzumab plus pertuzumab, we established an ER+/HER2-low/HER3+ human breast cancer mouse xenograft model using human breast cancer tissue derived from a woman with invasive lobular carcinoma. Mice were treated weekly with either lumretuzumab (10 mg/kg i.p.) or pertuzumab (15 mg/kg i.p. with a two-fold loading dose) as single-agents or in combination from Day 26 to Day 61 (six cycles). Single-agent lumretuzumab induced tumor stasis with a relative tumor volume (RTV) of 100% at Day 61 (Fig 2A). Pertuzumab monotherapy induced partial regression (RTV 61% at Day 61) whereas strong tumor regression was seen in mice treated with combination therapy (RTV 10% at Day 61). With regard to tumor growth inhibition, all treatment groups were significantly different to controls on Day 41. Tumor growth inhibition with combination therapy was significantly greater than with single agent treatments on Day 61 (S1 Statistical Analyses).

Mice were followed-up after discontinuation of antibody on Day 61 (Fig 2B). Regrowth of the HBCx-19 xenografts was seen in mice treated with either antibody as monotherapy but the combination of lumretuzumab and pertuzumab induced long-lasting tumor remission after treatment was discontinued.

Tumor remission in mice treated with the combination lumretuzumab/pertuzumab was associated with inhibition of HER2/HER3 signaling. In explanted tumors harvested from mice on Day 94 (Day 40 for vehicle controls), strong inhibition of the key downstream signaling molecules MAPK and AKT was seen in mice treated with the combination therapy (Fig 2C and 2D). Activation of AKT shows counter regulation of a compensatory pathway in tumors which escaped long-term lumretuzumab monotherapy.

To confirm these data, we investigated the efficacy of lumretuzumab and the combination with pertuzumab in a series of other ER+/HER2-low human breast cancer mouse xenograft models (Table 1). In all models tested, single-agent lumretuzumab induced moderate-to-strong tumor growth inhibition whereas the combination achieved the greatest efficacy (including complete tumor remission in two models).

**Table 1:** ER+/HER2-low human breast cancer mouse xenograft models and responses to single-agent lumretuzumab and the combination with pertuzumab.

	Tumor growth inhibition	
Xenograft (cell line/fragment-based)	Lumretuzumab monotherapy	Combination with pertuzumab
**T47D (cell line)**	> 100%	n.d.
**ZR-75-1 (cell line)**	moderate	additive effect
**ZR-75-1 (fragment)**	100%	n.d.
**MCF-7 (cell line)**	> 100%	n.d.
**MDA-MB-175 (cell line)**	47%-100%	complete remission
**HBCx-19 (fragment)**	100%	complete remission

TGI, tumor growth inhibition; n.d. not done

### HER2/HER3 crosstalk with the estrogen receptor

**Fig 3:**
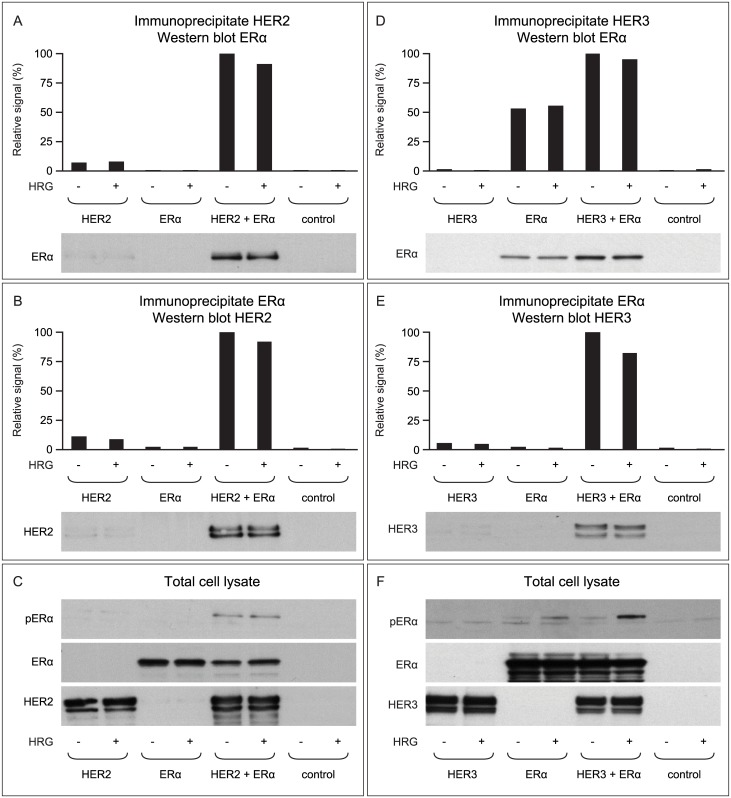
HER2 and HER3 both interact and co-immunoprecipitate with the ERα. (**A–C**) HEK 293 cells transfected with human *HER2* and *ERα* (alone or together) and stimulated with (+) or without (-) HRG were either immunoprecipitated with an anti-HER2 antibody and blotted against ERα (**A**) or vice versa (**B**). Results show that HER2 and ERα co-precipitated in cells transfected with cDNAs encoding both proteins, indicating that HER2 and ERα can form a complex. Western blotting of total cell lysate revealed that the ERα was phosphorylated only in cells that expressed both HER2 and ERα (**C**). The experiments were also conducted in HEK 293 cells transfected with human HER3 and ERα (alone or together; **D–F**). Again, HER3 and ERα co-precipitated (**D&E**) and ERα was only phosphorylated in HRG-treated cells expressing both HER3 and ERα (**F**). Data represent the results of 2–3 independent experiments.

To better understand the crosstalk of HER family members and the ER, we investigated the direct interaction between HER1/HER2/HER3 and ERα in a cellular model system. Lysates were prepared from HEK 293 cells transfected with human *HER2*, human *ERα*, or both *HER2* and *ERα*. The HEK 293 cells in these experiments were cultured in the absence of estrogen and with low (0.5%) levels of FCS. When HER2 was immunoprecipitated from this lysate and the resulting preparation was Western blotted using an anti-ERα antibody, ER co-precipitated with HER2 only in cells transfected with both proteins (Fig 3A). This indicates a direct and specific interaction between HER2 and ERα in transfected cells that express both proteins. Similar results were seen when ERα was immunoprecipitated from the lysate and the resulting preparation was Western blotted using an anti-HER2 antibody (Fig 3B). Importantly, ERα was phosphorylated (on serine 118) only in the cells that expressed both HER2 and ERα (Fig 3C).

In a similar set of experiments, we also demonstrated that HER3 forms a specific and direct complex with the ERα. Again, HER3 and ERα were shown to co-precipitate, indicating that HER3 and ERα can also form a complex (Fig 3D and 3E). This complex formed in both the presence and absence of HRG; however, ERα was only strongly phosphorylated in the presence of HRG (Fig 3F).

There was no evidence for crosstalk between HER1 and ERα.

### Anti-estrogen therapy further improves the efficacy of lumretuzumab and pertuzumab and leads to long-lasting responses

Based on these data showing that HER2 and HER3 can interact with the ERα, we investigated whether adding an agent that targets the ER could further improve on the efficacy seen with the lumretuzumab plus pertuzumab combination. The same ER+/HER2-low/HER3+ human breast cancer HBCx-19 xenograft model used previously was treated with lumretuzumab (3 mg/kg i.p.), pertuzumab (3 mg/kg i.p.), and the anti-estrogen fulvestrant (50 mg/kg i.m.), either as single-agents or in combination. Lower (sub-optimal) doses of lumretuzumab and pertuzumab than in previous experiments were used to better discriminate any additional efficacy of fulvestrant.

**Fig 4:**
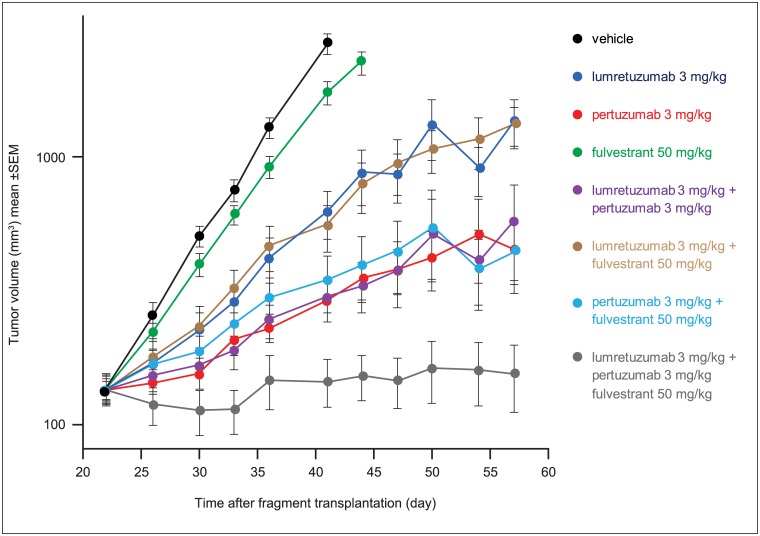
Adding anti-estrogen therapy further improves the efficacy of lumretuzumab and pertuzumab. HBCx-19 ER+/HER2-low/HER3+ breast cancer xenograft-bearing mice (n = 10 per treatment group) were treated with lumretuzumab (3 mg/kg i.p.), pertuzumab (3 mg/kg i.p.), and fulvestrant (50 mg/kg i.m.) either as single agents or in combination. Lower (sub-optimal) doses of lumretuzumab and pertuzumab were used to discriminate the additional contribution of fulvestrant. All treatments were given weekly beginning on Day 26 when median tumor size was 100–150 mm^3^ for 6 weeks (until Day 57).

The triplet combination was the most efficacious of all tested regimens, achieving an RTV on Day 57 of 118% (Fig 4). The monotherapies and dual combinations of lumretuzumab and pertuzumab at low doses were less efficacious and the HBCx-19 tumors progressed. All treatment groups were significantly different to controls on Day 41 (S1 Statistical Analyses).

### Patient case study

**Fig 5:**
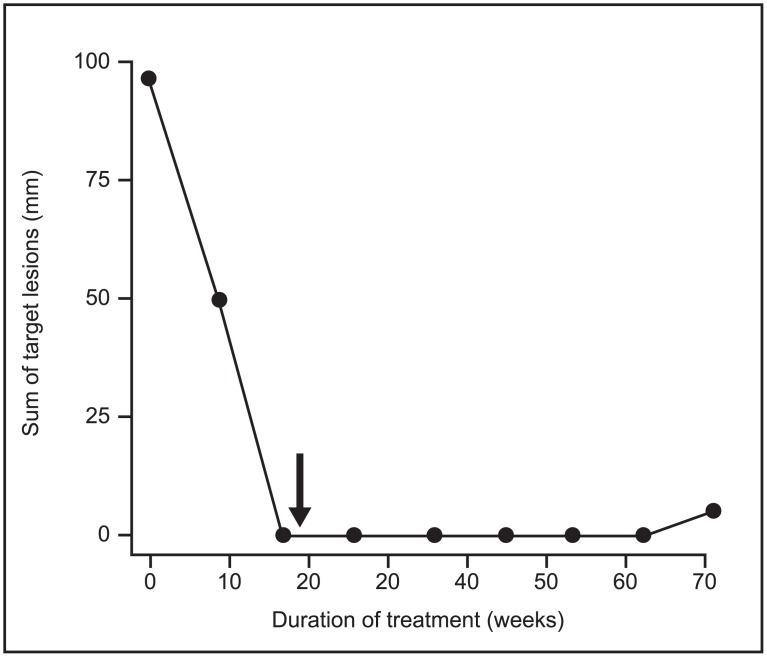
Tumor regression in a breast cancer patient treated with lumretuzumab plus pertuzumab and paclitaxel. This 37-year-old patient received 23 cycles of lumretuzumab and pertuzumab. The black arrow indicates the discontinuation of paclitaxel treatment after 18 weeks. Circles indicate the timing of tumor assessments. Tumors were assessed according to RECIST version 1.1.

A 37-year-old woman with ER+/HER2-low breast cancer participating in the NCT01918254 clinical trial [41] and treated with the combination of lumretuzumab plus pertuzumab was selected for a detailed analyses in a case study. A baseline tumor biopsy had been taken prior to enrollment to this study. Biomarker analyses revealed that the patient’s tumor was HER3+ (with an immune reactive score of 2.4), HER2-low (immunohistochemistry 1+), ER+, and PR+. In addition, targeted mutational analysis revealed a somatic mutation in *p53*, a somatic mutation of unknown function in the *HER2* gene (c. 1466C>T), but no *ESR1* mutations were detected [41]. The patient was diagnosed in October 2007 and received neoadjuvant treatment with doxorubicin and paclitaxel for four cycles followed by three more courses of CMF (cyclophosphamide, methotrexate, and 5-FU). In May 2008 a mastectomy and lymphadenectomy of the right breast was done. Subsequently, the patient received adjuvant radiotherapy of 50 Gy and treatment with the selective estrogen receptor modulator tamoxifen from 2008 to 2013. Metastatic disease was discovered in November 2013 while the patient was on tamoxifen and the patient was enrolled into the study in December 2013. This patient received lumretuzumab at a dose of 500 mg every three weeks (q3w) with pertuzumab (a loading dose of 840 mg and a maintenance dose of 420 mg q3w) and paclitaxel (80 mg/m^2^ every week); all administered as intravenous infusions. Paclitaxel was given for six three-weekly cycles only. After five cycles, the paclitaxel dose was reduced from 80 to 60 mg/m^2^ and after six cycles paclitaxel treatment was discontinued (both due to paresthesia of the patient). The patient continued to receive lumretuzumab and pertuzumab alone for a total of 23 cycles. Target lesions included one lesion in the left breast and one lesion in the mediastinum. Non-target lesion included pleural effusion and multiple axillary adenopathies. At the first tumor assessment after nine weeks of treatment the patient had a partial response with a decrease of the target lesions by 49% (according to RECIST version 1.1 criteria). From the second tumor assessment onwards, the patient showed a complete response, i.e. a complete disappearance of all target and non-target lesions (Fig 5). The duration of response was 14.6 months before the patient eventually discontinued the study due to progressive disease with regrowth of target and non-target lesions and development of new skin lesions. The progression-free survival for this patient was 16.7 months.

## Discussion

Our in vitro results demonstrate a direct interaction between ER, HER2 and HER3 with activation of ER. The triple combination of anti-HER2, anti-HER3, and anti-estrogen therapy lead to long lasting responses *in vivo*. Finally, we show a case study in a breast cancer patient heavily pretreated with chemotherapy and the anti-estrogen tamoxifen whose tumor responded to the combination of lumretuzumab plus pertuzumab.

Growing preclinical and clinical evidence indicates that a complex molecular bidirectional crosstalk exists between ER and HER2 [30,43]. ER expression is downregulated in a letrozole-resistant HER2-low xenograft mouse model [44]. In this model, addition of trastuzumab to letrozole increases levels of ER, restores sensitivity to letrozole, and leads to superior efficacy of the trastuzumab/letrozole combination. First results from clinical studies combining endocrine therapies with targeted therapies such as trastuzumab or everolimus in HER2+ disease are consistent with these preclinical data [45,46].

As single agents, the anti-HER3 antibody lumretuzumab and the anti-HER2 antibody pertuzumab lead to efficient blocking of HER3 and HER2 signaling, respectively, inhibiting the HER2:HER3 heterodimer and downstream signaling cascades *in vitro* and in animal models [37,39]. We demonstrated the additional benefit of dual HER2/HER3 blockage compared with single-agent lumretuzumab or pertuzumab alone in ER+ mouse xenograft models of human breast cancer that expressed HER3 and low levels of HER2 (Fig 2 and Table 1). Long-lasting tumor regression and complete inhibition of the central downstream signaling molecules AKT and MAPK was achieved only with the combination of lumretuzumab and pertuzumab.

The activation of compensatory mechanisms under treatment leading to resistance and tumor regrowth is seen with many targeted therapies including HER2+ tumors treated with trastuzumab, HER1+ tumors treated with cetuximab, and for hormone-dependent tumors treated with endocrine therapies [23,28,47]. To better understand the cross talk between HER family members and the ER, we examined the interaction between these receptors in HEK 293 cells transfected with HER2 or HER3 cDNAs and ER cDNA. We showed that the expression of HER2 or HRG-activated HER3 in breast cancer cell lines lead to phosphorylation and thus activation of ER (Fig 3), confirming the direct cross talk between these receptors. We could not demonstrate a direct cross talk between HER1 and ER in the HEK 293 cell model system. Most interesting was the demonstration of a physical complex formed by HER2 and ER as well as HER3 and ER (Fig 3). We hypothesize that direct interaction of the HER tyrosine kinases with the ER leads to the ER activation and is involved in the compensatory targeted therapy resistance mechanisms.

A combination of the best possible HER family targeted therapies (lumretuzumab plus pertuzumab) with an endocrine therapy (fulvestrant) subsequently lead to superior and long-lasting efficacy in an ER+/HER2-low/HER3+ breast cancer mouse xenograft model (Fig 4). As the combination of lumretuzumab plus pertuzumab at efficacious doses led to strong tumor regression (Fig 2), we employed lower doses of lumretuzumab and pertuzumab to enable us to see any additional efficacy provided by endocrine therapy. As a result, the 3 mg/kg doses of lumretuzumab and pertuzumab reduced the efficacy of the single-agents and the combination (Fig 4) compared with the tumor stasis observed with single-agent treatment with the higher doses (lumretuzumab 10 mg/kg and pertuzumab 15 mg/kg) and tumor regression with the combination shown in Fig 2.

The clinical value of the anti-HER3 and anti-HER2 combination strategy was demonstrated in a 37-year-old woman with ER+/HER2-low breast cancer who relapsed with metastatic disease while receiving adjuvant endocrine therapy with tamoxifen (Fig 5). After achieving a partial response under the triple combination of paclitaxel, pertuzumab, and lumretuzumab, the chemotherapy was discontinued due to toxicity and the patient continued with the antibodies alone, subsequently achieving a complete response. The duration of response of 14.6 months and a PFS of 16.7 months compares favorably to data generated with paclitaxel-based therapies in this setting [48,49] and is in the range of what has been reported for HER2+ patients treated with trastuzumab and pertuzumab [50]. This case study provides an example of a strong clinical response to the combination of lumretuzumab and pertuzumab in a patient who progressed on tamoxifen. This case study may be the first clinical indication of a compensatory cross talk between HER2:HER3-driven signaling pathways and ER-mediated signaling that could be therapeutically targeted by pertuzumab and lumretuzumab. These initial data need to be interpreted with caution as we cannot rule out that beneficial clinical or biological characteristics may have contributed to this favorable clinical course of this patient with the triple combination. Lumretuzumab was evaluated In HER2/HER3 positive patients with metastatic breast cancer (NCT01918254) in combination with pertuzumab and paclitaxel.

In conclusion, we demonstrate a direct interaction between the ER and HER2/HER3 which may underlie the development of resistance to these frequently targeted molecules in breast cancer. Our preclinical data also show that a triplet combination consisting of anti-HER3 (lumretuzumab), anti-HER2 (pertuzumab), and endocrine (fulvestrant) therapy achieved long-lasting tumor responses *in vivo*, whereas our case study demonstrates a clinical benefit of combined lumretuzumab and pertuzumab in a ER+/HER2-low breast cancer patient. Thus, the combination of lumretuzumab and pertuzumab with endocrine therapy may be an efficacious treatment strategy in patients with ER+/HER2-low breast cancer, an area of significant unmet medical need.

## Supporting information

S1 Dataset Raw data used to create charts in the manuscript. (ZIP)

S1 Protocol Protocol for the study from which the patients case study in this paper is derived. (PDF)

S1 Statistical Analyses Statistical analyses of the differences in tumor growth inhibition in the different treatment groups compared with control animals, and between combination therapy and single agent therapy (*in vivo* experiments; Figs 2a, 2b and 4). (DOCX)
