# Supplementary material for: Direct estrogen receptor (ER) / HER family crosstalk mediating sensitivity to lumretuzumab and pertuzumab in ER+ breast cancer
Source: PLoS One. 2017 May 11;12(5):e0177331. doi: 10.1371/journal.pone.0177331 (PMC5426757; doi:10.1371/journal.pone.0177331)
Supplement: S1 Protocol — (PDF) [file pone.0177331.s002.pdf]

PROTOCOL

**TITLE:**	**PHASE IB, OPEN-LABEL, MULTICENTER, DOSE-ESCALATION STUDY FOLLOWED BY AN EXTENSION PHASE TO EVALUATE THE SAFETY, PHARMACOKINETICS AND ACTIVITY OF RO5479599, A GLYCOENGINEERED ANTIBODY AGAINST HER3, ADMINISTERED IN COMBINATION WITH PERTUZUMAB AND PACLITAXEL IN PATIENTS WITH METASTATIC BREAST CANCER EXPRESSING HER3 & HER2 PROTEIN.**
**PROTOCOL NUMBER:**	BP28752
**VERSION NUMBER:**	E
**EUDRACT NUMBER:**	2013-000090-67
**IND NUMBER:**	To be determined
**TEST PRODUCT:**	RO5479599
**MEDICAL MONITOR:**	Martin Weisser
**SPONSOR:**	F. Hoffmann-La Roche Ltd
**DATE FINAL:**	See electronic date stamp below

FINAL PROTOCOL APPROVAL

**Approver's Name**	**Title**	**Date and Time (UTC)**
Weisser,Martin	Translational Medicine Leader	25-Dec-2014 16:03:12

CONFIDENTIAL STATEMENT

The information contained in this document, especially any unpublished data, is the property of F.Hoffmann-La Roche Ltd (or under its control) and therefore is provided to you in confidence as an investigator, potential investigator, or consultant, for review by you, your staff, and an applicable Ethics Committee or Institutional Review Board. It is understood that this information will not be disclosed to others without written authorization from Roche except to the extent necessary to obtain informed consent from persons to whom the drug may be administered.

SYNOPSIS OF PROTOCOL NUMBER BP28752

TITLE	**Phase Ib, open-label, multicenter, dose-escalation study followed by an extension phase to evaluate the safety, pharmacokinetics and activity of RO5479599, a glycoengineered antibody against HER3, administered in combination with pertuzumab and paclitaxel in patients with metastatic breast cancer expressing HER3 & HER2 protein.**
PROTOCOL NUMBER:	BP28752
VERSION NUMBER:	E
EUDRACT NUMBER:	2013-000090-67
IND NUMBER:	To be determined
TEST PRODUCT:	RO5479599
CLINICAL PHASE	Phase Ib
SPONSOR	F. Hoffmann-La Roche, Ltd.
INDICATION	Metastatic breast cancer expressing HER3 & HER2 protein
OBJECTIVES	**Primary:**<br>• To describe the safety profile and PK of selected doses of RO5479599 in combination with pertuzumab and paclitaxel in patients with MBC expressing HER2 and HER3 protein.<br>• To determine the recommended phase II dose(s) (RPTD) and schedule(s) for RO5479599 in combination with pertuzumab and paclitaxel.<br>**Secondary:**<br>• To describe the preliminary anti-tumor activity of RO5479599 in combination with pertuzumab and paclitaxel by assessing objective response rate (ORR, including patients with confirmed complete response [CR] or confirmed partial response [PR]), disease control rate (DCR, including patients with an objective response or stable disease [SD]), and PFS.<br>• To describe the pharmacodynamic (PD) effects of RO5479599 in combination with pertuzumab and paclitaxel.<br>**Exploratory:**<br>• To describe the relationship between PK and PD results.<br>• To describe the relationship between PK and clinical outcomes.<br>• To explore the association between response prediction biomarkers and clinical outcome.
STUDY DESIGN	**Description of Study**<br>This is an open-label, multicenter, phase Ib clinical study to investigate the safety profile and PK profile of selected doses of RO5479599 in combination with pertuzumab and paclitaxel in patients with MBC expressing HER3 and HER2 protein.<br><br>RO5479599 will be administered on a q3w schedule (i.e., on day 1 of 3-weekly cycles). Pertuzumab will be administered at a loading dose of 840 mg during cycle 1 followed by 420 mg for the remaining cycles in a q3w schedule. Paclitaxel will be administered at 80 mg/m^2 in a qw schedule.

The study will have a dose-escalation part which includes a dose escalation with 6 patients per dose cohort and an extension phase with up to 14 additional patients recruited per cohort. If the first 6 evaluable patients enrolled in the 1000 mg cohort of RO5479599 fulfill the safety criteria then escalation to the 2000 mg cohort of RO5479599 in combination with pertuzumab and paclitaxel will be initiated. Specifically, if 0 or 1 out of the first 6 evaluable patients treated at the 1000 mg dose level experiences a DLT, then the dose of RO5479599 will be escalated to 2000 mg. If 2 out of 6 patients experience a DLT, the decision to escalate to 2000 mg will be taken after the sponsor and investigators have reviewed and concluded that the severity and the type of the AEs are medically acceptable. If 3 out of 6 patients experience a DLT then enrollment will not be started for the 2000 mg dose cohort and stopped for the 1000 mg dose cohort. If 0 or 1 out of the first 6 evaluable patients treated at the 2000 mg dose level experience a DLT, then the recruitment of up to a total of 20 patients in this cohort will be initiated (depending on the drop-out rate). If 2 out of 6 patients treated at the 2000 mg dose level experience a DLT, the decision to continue recruitment in this cohort will be taken after the sponsor and investigators have reviewed and concluded that the severity and the type of the AEs are medically acceptable. If 3 out of 6 patients experience a DLT then enrollment is stopped.

Depending on the toxicities observed there is the option to expand also the 1000 mg cohort to a total of 12 to 20 patients in this cohort. If the drop-out rate is approximately 10% or above (≥ 2 out of 20 patients not completing week 12 of treatment) then patients may be replaced.

In each dose escalation cohort of RO5479599 in combination with pertuzumab and paclitaxel, 6 patients will be enrolled in parallel except for the first two patients where there will be a one-week window between the patients.

Should the 1000 mg cohort not fulfill the safety criteria defined above the respective cohort will not be expanded and the higher dose level will not be initiated. Instead lower dose levels (below 1000 mg of RO5479599) of RO5479599 may be evaluated following the same rules as described above with a 6 patient dose-escalation phase followed by an expansion phase.

After determination of a safe dose of RO5479599 in combination with pertuzumab and paclitaxel, another extension phase with approximately 20 MBC patients will be recruited. Only patients without prior chemotherapy for metastatic disease and/or who have received a maximum of one prior chemotherapy regimen in the adjuvant or neoadjuvant setting will be selected.

In order to explore possible improvements of the safety profile of the combination treatment (especially regarding diarrhea) the Extension Phase Cohort 2 will commence at a reduced dose of pertuzumab, i.e. 420 mg for Cycle 1 followed by 420 mg for subsequent cycles. The first 6 patients will be observed for the incidence and intensity of diarrhea for 2 cycles before expanding the cohort to 20 patients. If 0 or 1 out of the first 6 evaluable patients experiences a Grade ≥3 diarrhea (as defined for DLTs below), then further recruitment in this cohort will be initiated to a total of 20 patients. If 2 out of 6 patients experience a Grade ≥3 diarrhea, recruitment will only be continued after the sponsor and investigators have reviewed and concluded that the severity and the type of the diarrhea is medically acceptable. If 3 or more out of 6 patients experience

a Grade ≥3 diarrhea then enrollment is stopped at this pertuzumab and RO5479599 dose. Dependent on the outcome of this safety cohort a further dose modification of pertuzumab and/or RO5479599 based on safety and exposure data generated may then be implemented and evaluated in an additional safety cohort of n=6 patients plus extension phase cohort following the same decision making criteria as described above.

The sample size for the dose escalation phase considered here is tailored to the following considerations: If the true probability of DLT is 30%, the probability of observing 2 out of 6 DLTs is 32%; if the true probability of DLT is 15%, the probability of observing 2 out of 6 DLTs is 18%. The cohort sample size of the high dose cohort considered here is tailored to the following decision criteria: A dose-level of RO5479599 in combination with pertuzumab and paclitaxel will be defined as safe if, after having observed 20 patients, there is at least a 50% (posterior) probability that toxicity rate is less than or equal to 20%.

A Bayesian beta-binomial model on the probability of DLT will be applied to inform the decision-making (the model can be updated with the observed data at any time-point in the study). Both an informative and uninformative prior will be explored on the probability of toxicity. Prior information on the toxicities of the pertuzumab and paclitaxel combination will be embedded in the informative prior assumptions.

DLTs: For the purpose of this study, a DLT will be defined as any of the following events related to RO5479599 and occurring during the first cycle (1 cycle = 3 weeks; DLT period = 21 days):

- Hematological toxicities:
  - Grade 4 neutropenia (i.e. absolute neutrophil count [ANC] < 0.5×10^9 cells/L for minimal duration of 7 days).
  - Febrile neutropenia (i.e. ANC < 1.0×10^9 cells/L with a single temperature of > 38.3°C or a sustained temperature of ≥ 38°C for more than 1 h).
  - Grade 4 thrombocytopenia (≤ 25.0×10^9 cells/L).
  - Grade 3 thrombocytopenia associated with severe bleeding episodes.
- Grade ≥ 3 non-hematological toxicity with the exception of:
  - Alopecia (any grade).
  - Grade 3 nausea and vomiting and diarrhea that respond to optimal management.
  - Grade 3 diarrhea lasting for ≤ 2 days with no fever or dehydration.
  - Laboratory values of ≥ grade 3 which are judged not clinically significant by the investigator.
  - Infusion-related reactions (IRRs) are not considered DLTs since, based on experience with monoclonal antibodies, IRRs are not dose-related events. Precautions will be taken if IRRs grade ≥ 2 occur. If described precautions are not sufficient, other options will be discussed between sponsor and investigator.

- Failure to recover from any treatment-related toxicity grade ≥ 2 which results in a dose delay of > 14 days of the next scheduled

	administration will be considered a DLT.<br>• Grade 3 neuropathy that causes a dose delay of > 14 days will be considered a DLT.
NUMBER OF PATIENTS	Dose escalation phase: n = 6 evaluable patients per cohort will be enrolled in this part.<br>Extension phase 1: Approximately n = 14 patients in the 2000-mg cohort. Extension phase 2: Approximately n = 20 patients with no prior chemotherapy for MBC and/or a maximum of one prior chemotherapy regimen in the adjuvant or neoadjuvant setting.<br>Overall, approximately n = 46 to 66 patients are anticipated.
TARGET POPULATION	Patients with metastatic breast cancer expressing HER3 & HER2 protein

INCLUSION/EXCLUSION CRITERIA	**Inclusion criteria (dose escalation phase and extension phase 1):**<br>1. Signed informed consent, ability and willingness to comply with all aspects of the protocol, as required by the investigator.<br>2. Age ≥ 18 years.<br>3. ECOG performance status (PS) 0 to 1.<br>4. Histologically confirmed MBC patients eligible for enrollment must be willing to undergo a fresh (pretreatment) tumor/metastases biopsy that will be used to assess the level of HER3 protein expression by IHC and central pathology review. A formalin fixed paraffin embedded tumor (FFPET) block of the core needle biopsy[1] must be submitted for central IHC staining and review. Fine needle aspiration (FNA), biopsy of bone lesions, or archived primary tumor tissue is not acceptable to determine eligibility. Core needle biopsy specimens should contain at least 100 viable neoplastic cells. Discernible HER3 membrane positivity in any neoplastic cell will be considered diagnostically positive for HER3 protein expression and eligible for enrollment.<br>5. HER2 status confirmed on same tumor/metastases by a central laboratory: BC tumors and/or metastases must be HER2 IHC 1+/ISH- or HER2 IHC 2+/ISH- as assessed by parallel testing of protein and gene amplification using an FDA-approved test.<br>6. Taxane-naïve patients or patients who have received taxanes as part of an adjuvant/neoadjuvant treatment regimen with a disease-free interval of at least 1 year. Patients who have received a docetaxel-containing regimen in the metastatic setting may be eligible. Patients who have received paclitaxel/nab-paclitaxel in the metastatic setting but have discontinued paclitaxel/nab-paclitaxel for a reason other than disease progression and have had a taxane-free interval of at least 6 months may be eligible unless otherwise contraindicated at the investigator's discretion.<br>7. Radiologically measurable or clinically evaluable disease according to RECIST criteria.<br>8. Last dose of systemic anti-neoplastic therapy > 21 days prior to first study treatment infusion. Palliative radiotherapy is allowed up to 2 weeks before the first study treatment infusion.<br>9. All acute toxic effects of any prior radiotherapy, chemotherapy or surgical procedure must have resolved to Grade ≤ 1, except alopecia (any grade).<br>10. Adequate hematological function: neutrophil count of ≥ 1.5 x 109 cells/L, platelet count of ≥ 100,000/µL, hemoglobin (Hb) ≥ 10 g/dL (6.2 mmol/L).<br>11. Adequate liver function: Bilirubin ≤ 1.5 x ULN (excluding Gilbert's Syndrome, see below), aspartate aminotransferase (AST) and/or alanine aminotransferase (ALT) ≤ 2.5 x ULN (in case of liver metastases or patients with hepatocellular

[1] For superficial lesions a punch biopsy can be performed instead if an adequately deep specimen can be warranted and including a representative amount of tumor cells. Therefore, the punch biopsy should have a diameter of ≥ 2 mm at least.

carcinoma: ≤ 5 x ULN).

12. Adequate renal function: serum creatinine ≤ 1.5 ULN or creatinine clearance (by Cockcroft Gault formula [see Appendix 4] ≥ 50 mL/min for patients in whom, in the investigator's judgment, serum creatinine levels do not adequately reflect renal function.
13. Baseline left ventricular ejection fraction (LVEF) of ≥ 50% (measured by echocardiography).
14. Negative serum pregnancy test within 7 days prior to start of study treatment in premenopausal women and women ≤ 2 years after menopause (menopause is defined as amenorrhea for > 2 years).
15. Female patients must be postmenopausal, surgically sterile, or they must agree to use a highly effective form of contraception by the patient and/or partner. Contraception use must continue for the duration of the study and for at least 6 months after the last dose of study medication.
16. Male patients must agree to use a barrier method (condom) during the study and for at least 6 months after last dose of study medication.

Gilbert's Syndrome

Patients with Gilbert's Syndrome will be eligible for the study. The diagnosis of Gilbert's Syndrome is suspected in people who have persistent, slightly elevated levels of unconjugated bilirubin without any other apparent cause. A diagnosis of Gilbert's syndrome will be based on the exclusion of other diseases based on the following criteria:

- Unconjugated hyperbilirubinemia noted on several occasions
- No evidence of hemolysis (normal hemoglobin, reticulocyte count and lactate dehydrogenase [LDH])
- Normal liver function tests
- Absence of other diseases associated with unconjugated hyperbilirubinemia

Extension phase 2:

All of the above except inclusion criterion #6. In addition:

Patients with no prior chemotherapy for MBC and/or a maximum of only one prior chemotherapy regimen in adjuvant or neoadjuvant setting.

Taxane-naïve patients or patients who have received taxanes as part of an adjuvant/neoadjuvant treatment regimen with a disease-free interval of at least 1 year.

Exclusion criteria (all patients):

1. Concurrent therapy with any other investigational drug (defined as treatment for which there is currently no regulatory authority approved indication).
2. History or clinical evidence of central nervous system (CNS) primary tumors or metastases including leptomeningeal metastases unless they have been previously treated, are asymptomatic and have had no requirement for steroids or

	enzyme-inducing anti-convulsants in the last 14 days.<br>3. Evidence of significant, uncontrolled concomitant diseases which could affect compliance with the protocol or interpretation of results, including uncontrolled diabetes mellitus.<br>4. Active or uncontrolled infections.<br>5. Known human immunodeficiency virus (HIV) or known active hepatitis B virus (HBV) or hepatitis C virus (HCV) infection.<br>6. Any other diseases, metabolic dysfunction, physical examination finding or clinical laboratory finding giving reasonable suspicion of a disease or condition that would contraindicate the use of an investigational drug.<br>7. Major surgery or significant traumatic injury < 28 days prior to the 1st study treatment infusion (excluding biopsies) or anticipation of the need for major surgery during study treatment.<br>8. Dementia or altered mental status that would prohibit informed consent.<br>9. Pregnant or breast-feeding women.<br>10. Known hypersensitivity to any of the components of RO5479599, pertuzumab or paclitaxel.<br>11. Patients with contraindications for paclitaxel therapy according to the SmPC (see http://www.actavis.ie/NR/rdonlyres/6FEC3CFF-8F21-4FEE-B7BB-4198577A99E0/18005/3SPC_PA1380591_230712_C2106797.pdf).<br>12. Therapy with an antibody or immunotherapy (i.e., interferon-α, interferon-β, interleukin-2, etanercept, infliximab, tacrolimus, cyclosporine, mycophenolic acid, alefacept or efalizumab) concurrently or within a period of time where drug exposure is still considered biologically active (usually < 5x $t_{1/2}$) prior to first dose of study treatment.<br>13. Regular immunosuppressive therapy (i.e., for organ transplantation, chronic rheumatologic disease).<br>14. Concurrent high doses of systemic corticosteroids. High dose is considered as > 20 mg of dexamethasone a day (or equivalent) for > 7 consecutive days.<br>15. Baseline QTc interval of > 470 ms, patients with baseline resting bradycardia < 45 beats per minute or baseline resting tachycardia > 100 beats per minute.<br>16. Uncontrolled hypertension (systolic > 150 mmHg and/or diastolic > 100 mmHg), unstable angina, congestive heart failure (CHF) of any New York Heart Association (NYHA) classification, serious cardiac arrhythmia requiring treatment (exceptions: atrial fibrillation, paroxysmal supraventricular tachycardia), history of myocardial infarction within 6 months of enrollment or symptomatic LVEF dysfunction.<br>17. A history of grade ≥ 3 peripheral neuropathy of any etiology.
LENGTH OF STUDY	Patients will be treated until disease progression, unacceptable toxicities, withdrawal from treatment for other reasons or death. The screening period will be 28 days, and the follow-up period will be 28

	(±3) days after the last dose of study treatment or up to 28 (±3) days after disease progression whichever comes first. A further PK/human anti-human antibody (HAHA) analysis will be conducted at visit on Day 42 to 45 for patients who are not entering another clinical study or receiving further anti-tumor treatment.<br>**End of Study**<br>The end of the study is defined as the time at which the last patient who started therapy within this study has progressed or has withdrawn consent or when the study is terminated by the Sponsor, whichever occurs first. After the post-treatment monitoring visit 1, patients will continue to be monitored for survival for up to 2 years.
**ASSESSMENTS**	
SAFETY OUTCOME MEASURES	The NCI CTCAE version 4.03 (http://evs.nci.nih.gov/ftp1/CTCAE/CTCAE_4.03_2010-06-14_QuickReference_8.5x11.pdf) will be used to evaluate the clinical safety of the treatment in this study. Patients will be assessed for AEs at each clinical visit and as necessary throughout the study.<br>Safety will be determined by the spontaneous reporting of AEs; by the assessments of routine laboratory values (hematology testing, serum chemistries, urinalysis); findings on physical examinations; electrocardiograms (ECGs); echocardiograms; vital signs; by carefully observing patients for IRRs; by the determination of HAHAs in those applicable cohorts.<br>**Laboratory Tests**<br>Hematology, blood chemistry and urinalysis, as listed below, will be collected at the timepoints indicated in the Schedule of Assessments.<br>1. Hematology: hemoglobin, hematocrit, erythrocytes (red blood cells [RBC]), platelets, leukocytes, white blood cell (WBC) differential (counts): neutrophils, eosinophils, basophils, monocytes and lymphocytes.<br>2. Coagulation: prothrombin time (PT)/international normalized ratio (INR) and partial thromboplastin time (PTT).<br>3. Blood chemistry: Aspartate aminotransferase (AST), alanine aminotransferase (ALT), γ-glutamyl transferase (GGT), total and conjugated bilirubin, alkaline phosphatase (ALP), lactate dehydrogenase (LDH), albumin, creatinine, creatinine clearance (by Cockcroft-Gault formula), total protein, glucose, sodium, chloride, calcium, phosphate, potassium, magnesium, urea.<br>4. Urinalysis: Dipstick for pH, glucose, blood, protein, bilirubin and ketones. If there is a clinically significant positive result (i.e. confirmed by a positive repeated sample), urine will be sent to the laboratory for microscopy and culture. If there is an explanation for the positive dipstick result, e.g. menses, it should be recorded, and there is no need to perform laboratory for microscopy and culture.<br>5. Serum pregnancy test in women of child-bearing potential.<br>Unscheduled hematology, biochemistries, urinalysis and coagulation may be obtained in patients who develop IRRs.<br>**Additional safety laboratory assessments**<br>• HAHA (for RO5479599 and pertuzumab)<br>• Leukocyte immunophenotyping<br>• IgE and tryptase

	• Organ biopsy (optional)
PHARMACOKINETIC OUTCOME MEASURES	Serum concentrations of RO5479599, pertuzumab and paclitaxel will be measured by a specific and validated LC-MS/MS method. PK parameters will be estimated using standard non-compartmental methods.<br>• Maximum serum concentration (C_{max})<br>• Trough serum concentration (C_t) at the beginning of Cycle 2 and at the beginning of every subsequent cycle where PK data are available<br>• Time to reach maximum serum concentration (t_{max})<br>• Area under the concentration-time curve (AUC)<br>• Total clearance (CL)<br>• Volume of distribution (V)<br>• Accumulation ratio<br>• Elimination half-life ($t_{1/2}$)<br>• Serum concentration at the time of tumor progression (C_{prog})<br>• Serum concentration at the time of tumor response (CR/PR)<br>• Serum concentration at the time of DLT<br>• Serum concentration at the time of tumor and skin biopsy (C_b)<br>• Serum concentration at time of IRR<br>PK of pertuzumab and paclitaxel will only be assessed for Extension Phase Cohort 2 patients.
EFFICACY OUTCOME MEASURES	Baseline total tumor burden will be assessed within 14 days before first dose of study treatment. Subsequent tumor evaluations will be performed every 9 weeks (±7 days) after the first dose of RO5479599.<br>Tumor response will be evaluated based on RECIST, version 1.1. Efficacy analysis will include ORR, DCR, duration of response and PFS. OS information will be collected during the time on study. Survival follow-up is planned up to 24 months. Confirmation of PRs and CRs will occur at the next scheduled tumor assessment, but at least 6 weeks after the initial response was noted.<br>Optional submission of the latest pre-study CT scan (historical CT scan) is highly encouraged if available. This scan will be compared to those collected during the study to determine tumor growth kinetics.
PHARMACODYNAMIC OUTCOME MEASURES	Specimens for PD and biomarker discovery will be collected from patients participating in this clinical study. In addition, primary archival tumor block will be collected if available. These samples will be collected to promote, facilitate and improve individualized healthcare by better understanding/predicting RO5479599 efficacy, dose responses, safety, RO5479599 mode of action, progression of disease.<br>**<u>Tumor biopsies</u>**<br>As a first priority a formalin fixed, paraffin embedded tumor sample (FFPET) is requested which will undergo analysis for assessing the level of HER3 protein expression and HER2 status based on protein expression and gene amplification. Centrally confirmed HER3 and HER2 positivity on a freshly taken pretreatment biopsy (FFPET) is an eligibility criterion for study entry. As a second priority a fresh frozen (FF, liquid nitrogen) tumor sample from the same

	lesion is requested to allow more quantitative biomarker assessment. Per time point, two to four biopsies will be taken from the tumor. To make sure that all relevant biomarkers related to HER pathways or ADCC can be addressed a third and fourth tumor biopsy per time point might be collected if feasible and safe for the patient. Details on preservation and processing of potential additional tumor biopsies will be given in the Lab Manual.<br>Additional assessments will be performed on the tumor tissues (FFPET and FF) provided the material is sufficient:<br>• Molecules relevant to HER signaling or alternative pathways, e.g. HER3, EGFR, HER2, heregulin or cMET.<br>• ADCC-related markers like CD16, CD3, CD56, CD3.<br>• Molecules describing inflammatory tumor microenvironment, e.g. macrophage subtyping.<br>• Gene expression analysis to investigate signaling molecules which are modulated after HER3-targeted treatment, e.g. HER1, HER2, HER3, HER4.<br>• Assessment of tumor-specific mutations including but not limited to KRAS, PIK3CA, PTEN, EGFR, cMET.<br>A second, optional tumor biopsy may be taken at disease progression/relapse at the discretion of the investigator.<br>**<u>Blood samples</u>**<br>Blood samples will undergo<br>• Immunophenotyping to examine peripheral immune modulation and related parameters: Leukocyte immunophenotyping<br>• NK lymphocyte functional assessment:<br>o NK cell activation measured by CD107a<br>• Tumor markers CEA and CA15-3<br>**<u>Archived primary tumor samples</u>**<br>Submission of primary archival tumor block, fixed in 10% neutral buffered formalin and embedded into paraffin (historical tumor sample), is not a mandatory requirement, but is highly encouraged if available. When submitted to the central laboratory, this tumor sample will be used to cut sections for subsequent biomarker analysis. Comparing molecular pattern from primary archived tumor samples with freshly taken tumor biopsies, representing the respective disease state, might help to better understand how markers like HER3, HER2 or EGFR develop over time and even more under treatment.<br>To protect tissue against oxidation and to allow IHC analysis in large batches an archived tumor block would be preferred. Any left-over tissue blocks will be sent back to the investigator site after completion of the study.
EXPLORATORY OUTCOME MEASURES	The exploratory outcome measures for this study include but are not limited to the relationship between PK and PD data, response prediction biomarkers and efficacy variables.
BIOMARKER/GENOTYPING SAMPLE COLLECTION	**<u>Clinical Genotyping: FcγRIII-158 polymorphism</u>**<br>A single 3 mL whole blood sample will be taken for DNA extraction from every patient prior to the 1st infusion. The DNA will be used to determine if polymorphism at Fcγ receptor affect the efficacy/safety of RO5479599 and/or pertuzumab. Data arising from this study will be subject to the same confidentiality as the rest of the study.

INVESTIGATIONAL MEDICINAL PRODUCTS	RO5479599 is a glycoengineered recombinant humanized monoclonal antibody (mAb) that is directed against the extracellular domain of human epidermal growth factor receptor 3 (HER3). RO5479599 will be administered IV on a q3w schedule (i.e., on day 1 of 3-weekly cycles).<br>Dose level cohort 1: 1000 mg (flat dose)<br>Dose level cohort 2: 2000 mg (flat dose)<br>Pertuzumab will be given as q3w IV infusion at 840 mg for the first infusion followed by 420 mg at the following infusions. **Extension Phase Cohort 2 will commence at a reduced dose of pertuzumab, i.e. 420 mg for Cycle 1 followed by 420 mg for subsequent cycles.**<br>Paclitaxel will be given as weekly IV infusion at 80 mg/m^2. Dose delays and dose modifications for paclitaxel are described in the protocol body.<br>All study treatments will be administered in the consecutive sequence of paclitaxel, pertuzumab and RO5479599. Study treatment should be given on the same day except for Cycle 1 where it is recommended to administer paclitaxel and pertuzumab on Day 1 and RO5479599 on Day 2.
NON-INVESTIGATIONAL MEDICINAL PRODUCTS	Administration of paclitaxel, pertuzumab and RO5479599 will be done in a setting with emergency equipment and staff trained to monitor and respond to emergency situations. Patients must be monitored during each infusion of each study treatment and following the completion of the infusion for any AEs as specified in the protocol<br>All patients must be premedicated prior to paclitaxel and RO5479599 administration to prevent severe hypersensitivity reactions/infusion-related reactions:<br>Premedication for paclitaxel may consist of dexamethasone (or its equivalent), promethazine (or its equivalent) and cimetidine or ranitidine according to institutional guidelines.<br>Patients who experience infusion-related symptoms with pertuzumab may be pre-medicated with paracetamol and antihistamines for subsequent infusions.<br>For RO5479599, 30 min prior to start of the 1st infusion, paracetamol (500 to 1000 mg orally) and diphenhydramine (25 to 50 mg orally or IV; or an alternative anti-histamine at an adequate dose) will be administered. As paclitaxel and pertuzumab will be given prior to RO5479599 the investigator may decide whether repetition of premedication with antihistamines is necessary based on the time interval between paclitaxel, pertuzumab and RO5479599 administration and the half life of the premedication used at the respective institution. If one patient develops an IRR of ≥ grade 3 during the 1st infusion of RO5479599, pre-medication including corticosteroids for subsequent patients may be explored in the course of the study and based on IRR episodes observation.

STATISTICAL METHODS:	
Safety	Safety of RO5479599 in combination with pertuzumab and paclitaxel will be evaluated through the analysis of DLTs (during the dose-escalation), AEs, laboratory tests (hematology and serum chemistry), physical exam, vital signs, ECG, echocardiogram and performance status. All patients who received at least one dose of RO5479599 will be included in the safety analyses. Safety results will be presented in descriptive summary tables and patient listings as appropriate for each part of the study. All summary data will be presented by treatment cohorts, sorted by dosing schedule. Comparison across the dose-levels/schedules investigated will be carried out descriptively, mainly with respect to the AEs that are included in the definition of DLT and are therefore considered the most relevant for the assessment of the combination safety profile. Bayesian estimation of probabilities of toxicities may be carried out and the probability of being above the desired toxicity levels will be tabulated.
Efficacy	Tumor response data will be reported using descriptive statistics. ORR and DCR will be summarized using relative frequencies and 90% confidence limits. Duration of response and PFS will be summarized using time-to-event analyses and Kaplan-Meier curves. OS will be summarized and, if considered matured enough, estimated from Kaplan-Meier curves. Only informal comparisons will be carried out between dose-levels/schedules with respect to efficacy endpoints, as the study sample size would not allow for formal comparisons for efficacy.
Pharmacodynamics:	Descriptive statistics will be used in summarizing peripheral blood PD markers. Graphical techniques will be employed to better understand the relationship of the PD markers with dose and time. Correlations between PK parameters, PD markers and clinical response will be investigated descriptively if sufficient data are available within each dose-level/schedule. Descriptive data tabulations and graphical techniques may be used for this purpose.
Pharmacokinetics	PK parameters will be derived from the serum concentrations and will be presented for each dose using descriptive statistics including but not limited to the number of patients (n), mean, standard deviation (SD), median, minimum (min), maximum (max), geometric mean and coefficient of variation (CV). The parameters will include e.g. maximum serum concentration (C_{max}), area under the concentration curve (AUC), total clearance (CL), volume of distribution at steady state (V_{ss}), accumulation ratio and terminal elimination half-life. Estimation of PK parameters may be performed using standard non-compartmental methods or compartmental approaches. Analysis of variance models may be used to investigate dose proportionality and accumulation of the two doses being investigated.<br><br>Exploratory analysis and graphical representations will be performed to assess the possible relationship between the PK of RO5479599 and selected biomarkers, efficacy or safety using NCA and CA approaches as appropriate. Exploratory analysis will be performed to assess the PK of pertuzumab and paclitaxel in combination with RO5479599.

SAMPLE SIZE JUSTIFICATION:

The cohort size of 20 patients is tailored to detect, with a specified confidence, the chance that the toxicity rate is below 20%. The decision criterion used is the following: A dose level of RO5479599 in combination with pertuzumab and paclitaxel is defined as safe if, given the number of toxicity events observed in 20 patients, the posterior probability that the toxicity rate less or equal to 20% is at least 50%. This is considered as sufficient confidence in the evaluation of the decision criteria for the purpose of monitoring the safety of each cohort. These considerations can be applied both for DLTs as well as other safety events of importance. A total of 10000 simulations were carried

out for each scenario and the posterior probability was calculated via a Beta-Binomial Bayesian model of the toxicity events. Informative priors may also be investigated, based on historical data on the toxicity of pertuzumab and paclitaxel.

A total of maximum 60 patients will be enrolled in the study, should only two dose cohorts be investigated. Should toxicity warrant evaluation of a third dose, then the maximum sample size may go up to a total of 66 patients.

OTHER CONSIDERATIONS:

Not applicable.

List of Prohibited Medications

The following treatments are not permitted during the study:

- Cytotoxic chemotherapy agents other than paclitaxel.
- Radiotherapy (as per inclusion criteria).
- Immunosuppressive therapy and chronically administered glucocorticoids (high dose is considered as > 20 mg of dexamethasone a day [or equivalent)] for > 7 consecutive days) as per exclusion criteria.
- Other systemic anti-neoplastic agents and targeted therapies.

If any of these therapies are needed, the patient will be considered to have evidence of progressive neoplastic disease and have experienced treatment failure with the study treatment.

Table of Contents **Page**

1. Background and Rationale 27
1.1 Background on Disease 27
1.2 Background on Study Treatments 27
1.2.1 Background on RO5479599 27
1.2.1.1 Previous Non-Clinical Studies 29
1.2.1.2 Previous Clinical Studies 33
1.2.2 Background on Pertuzumab 35
1.2.3 Background on Paclitaxel 36
1.3 Study Rationale and Benefit-Risk Assessment 37
1.3.1 Study Rationale 37
1.3.2 Rationale for Patient Selection Based on the HER3 & HER2 Protein Expression and HER2 Gene Amplification Status 39
1.3.3 Risk-Benefit Assessment 40
2. Objectives 43
2.1 Primary Objectives 43
2.2 Secondary Objectives 43
2.3 Exploratory Objectives 44
3. Study Design 44
3.1 Description of Study 44
3.1.1 Overview of Study Design 44
3.1.2 End of Study 46
3.2 Rationale for Study Design 46
3.2.1 Rationale for Dosage Selection 46
3.2.2 Dose Escalation Decision Criteria for the Dose Escalation and Extension Phases 48
3.2.2.1 Dose-Limiting Toxicity 49
3.2.2.2 Maximum Tolerated Dose 50
3.2.2.3 Recommended Phase Two Dose and Regimen 51
3.2.3 Rationale for Study Population 51
3.2.4 Rationale for Control Group 52
3.2.5 Rationale for Biomarker Assessments 52
3.3 Outcome Measures 52

3.3.1 Safety Outcome Measures 52
3.3.2 Pharmacokinetic and Pharmacodynamic Outcome Measures 53
3.3.2.1 Pharmacokinetic Outcome Measures 53
3.3.2.2 Pharmacodynamic Outcome Measures 53
3.3.3 Efficacy Outcome Measures 54
3.3.4 Patient-Reported Outcome Measures 54
3.3.5 Exploratory Outcome Measures 54
4. Materials and Methods 54
4.1 Center 54
4.2 Study Population 54
4.2.1 Recruitment and Replacement Procedures 54
4.2.2 Procedures for Screening Patients for HER3 Expression in Tumor Tissue and Assessment of HER2 Status 55
4.2.3 Inclusion Criteria 56
4.2.4 Exclusion Criteria (All Patients) 58
4.3 Method of Treatment Assignment 59
4.4 Study Treatment 60
4.4.1 Formulation, Packaging and Handling 60
4.4.1.1 RO5479599 60
4.4.1.2 Pertuzumab 61
4.4.1.3 Paclitaxel 61
4.4.2 Dosage and Administration 61
4.4.2.1 Paclitaxel 62
4.4.2.2 Pertuzumab 62
4.4.2.3 RO5479599 63
4.4.2.4 Schedule of Administration of Paclitaxel, Pertuzumab and RO5479599 66
4.4.2.5 Toxicity Assessments, Dose Delays and Dose Modifications for Toxicity 67
4.4.2.6 Management of Toxicities 70
4.4.3 Additional Required Medication 72
4.4.4 Investigational Medicinal Product Accountability 72
4.4.5 Assessment of Compliance 73

4.4.6 Post-Trial Access to RO5479599, Pertuzumab and Paclitaxel.....74
4.5 Prohibited Therapy.....74
4.6 Study Assessments.....74
4.6.1 Description of Study Assessments.....74
4.6.1.1 Medical History and Demographic Data.....74
4.6.1.2 Physical Examinations.....74
4.6.1.3 Vital Signs.....75
4.6.1.4 ECOG Performance Status.....75
4.6.1.5 Electrocardiograms.....76
4.6.1.6 Echocardiography.....77
4.6.1.7 Laboratory Assessments.....77
4.6.1.8 Special Safety Assessments.....78
4.6.1.9 Pharmacokinetic Assessments.....79
4.6.1.10 Exploratory Pharmacodynamic and Response Prediction Biomarker Assessments.....80
4.6.1.11 Disease-Specific Assessments.....84
4.6.2 Timing of Study Assessments.....85
4.6.2.1 Screening and Pre-Treatment Assessments.....85
4.6.2.2 Assessments during Treatment.....86
4.6.2.3 Assessments at Study Completion/Early Termination Visit/Follow-Up Assessments.....87
4.6.2.4 Assessments at Unscheduled Visits.....88
4.7 Patient, Study, and Site Discontinuation.....88
4.7.1 Patient Discontinuation.....88
4.7.1.1 Discontinuation from Study Treatment.....89
4.7.1.2 Withdrawal from Study.....89
4.7.2 Study and Site Discontinuation.....90
5. Assessment of Safety.....90
5.1 Safety Parameters and Definitions.....90
5.1.1 Adverse Events.....90
5.1.2 Serious Adverse Events (Immediately Reportable to the Sponsor).....91
5.1.3 Non-Serious Adverse Events of Special Interest (Immediately Reportable to the Sponsor).....91

5.1.4 Pregnancy and Contraception 92
5.2 Safety Plan 92
5.2.1 Management of Specific Adverse Events 92
5.3 Methods and Timing for Capturing and Assessing Safety Parameters 92
5.3.1 Adverse Event Reporting Period 92
5.3.2 Eliciting Adverse Event Information 93
5.3.3 Assessment of Severity of Adverse Events 93
5.3.4 Assessment of Causality of Adverse Events 94
5.3.5 Procedures for Recording Adverse Events 94
5.3.5.1 Diagnosis versus Signs and Symptoms 94
5.3.5.2 Adverse Events Occurring Secondary to Other Events 95
5.3.5.3 Persistent or Recurrent Adverse Events 96
5.3.5.4 Abnormal Laboratory Values 96
5.3.5.5 Abnormal Vital Sign Values 97
5.3.5.6 Abnormal Liver Function Tests 97
5.3.5.7 Deaths 97
5.3.5.8 Pre-Existing Medical Conditions 98
5.3.5.9 Lack of Efficacy or Worsening of Malignant Disease 98
5.3.5.10 Hospitalization or Prolonged Hospitalization 98
5.3.5.11 Overdoses 99
5.3.5.12 Immediate Reporting Requirements from Investigator to Sponsor 99
5.3.6 Emergency Medical Contacts 100
5.3.7 Reporting Requirements for Serious Adverse Events and Non-Serious Adverse Events of Special Interest 100
5.3.8 Reporting Requirements for Pregnancies 100
5.3.8.1 Pregnancies in Female Patients 100
5.3.8.2 Pregnancies in Female Partners of Male Patient 100
5.3.8.3 Abortions 101
5.3.8.4 Congenital Anomalies/Birth Defects 101
5.4 Follow-Up of Patients after Adverse Events 101
5.4.1 Investigator Follow-Up 101
5.4.2 Sponsor Follow-Up 101
5.5 Post-Study Adverse Events 101

5.6 Expedited Reporting to Health Authorities, Investigators, Institutional Review Boards, and Ethics Committees..........................102
6. Statistical Considerations and Analysis Plan 102
6.1 Determination of Sample Size 102
6.2 Summaries of Conduct of Study...104
6.3 Analysis Populations 104
6.3.1 Safety Analysis Population 104
6.3.2 Pharmacokinetic Analysis Population 104
6.3.3 Efficacy Analysis Population 104
6.4 Summaries of Treatment Group Comparability..................................104
6.5 Safety Analyses..104
6.5.1 Adverse Events 105
6.5.2 Clinical Laboratory Test Results...105
6.5.2.1 Standard Reference Ranges and Transformation of Data 105
6.5.2.2 Definition of Laboratory Abnormalities 106
6.5.3 Vital Signs 106
6.5.4 ECG Data Analysis..106
6.5.5 Echocardiogram Analysis...106
6.5.6 Concomitant Medications 106
6.6 Efficacy Analyses 106
6.6.1 Primary Efficacy Endpoint 107
6.7 Pharmacodynamic Analyses 107
6.8 Pharmacokinetic Analyses 108
6.9 Exploratory Analyses..109
6.10 Interim Analyses...109
7. Data Collection and Management 109
7.1 Data Quality Assurance..109
7.2 Electronic Case Report Forms 109
7.3 Electronic Patient-Reported Outcome Data 110
7.4 Source Data Documentation 110
7.5 Use of Computerized Systems..111
7.6 Retention of Records...111
8. Ethical Considerations 111
8.1 Compliance with Laws and Regulations...111

8.2 Informed Consent 111
8.3 Institutional Review Board or Ethics Committee 112
8.4 Confidentiality 112
8.5 Financial Disclosure 113
9. Study Documentation, Monitoring, and Administration 113
9.1 Study Documentation 113
9.2 Site Inspections 114
9.3 Publication of Data and Protection of Trade Secrets 114
9.4 Protocol Amendments 114
10. References 115

List of Tables Page

Table 1 Phase I Study BP27771: GeoMean (%CV) RO5479599 PK Parameters during Cycle 1 in HER3+ Advanced/Metastatic Solid Tumor Patients following Ascending Doses from 100 mg to 2000 mg q2w 35

Table 2 Projected Target Saturation in Humans following RO5479599 IV Doses q3w for Two Consecutive Cycles (Cycle 1 and 2) 48

Table 3 Pre-Medication for RO5479599 65

Table 4 Dose Delays and Dose Modifications of Paclitaxel 68

Table 5 Dose Delays and Dose Modifications of Pertuzumab, RO5479599 and Paclitaxel in Case of Diarrhea 69

Table 6 Adverse Event Severity Grading Scale 93

Table 7 Decision Criteria for Safety Based on Posterior Probabilities 103

Table 8 Probability of Observing DLTs in the Dose Escalation` Phase 103

List of Figures **Page**

Figure 1 Study Design 44

Figure 2 Simulated Pharmacokinetic Profiles of RO5479599 following 1000 mg and 2000 mg q3w for Four Consecutive Cycles with 50% Confidence Bounds (25th to 75th Percentile)... 47

Figure 3 Asymptotic Decline in LVEF: Algorithm for Continuation and Discontinuation of Pertuzumab Based on LVEF Assessments 72

List of Appendices

Page

Appendix 1 Schedule of Assessments 121
Appendix 2 Specific Schedule of Assessments for PK and HAHA.................125
Appendix 3 NYHA Classification..130
Appendix 4 Cockcroft-Gault Formula for Calculation of Creatinine Clearance 131
Appendix 5 Eastern Cooperative Oncology Group Performance Status Scale 132
Appendix 6 ICH Guidelines for Clinical Safety Data Management, Definitions and Standards for Expedited Reporting, Topic E2 133

LIST OF ABBREVIATIONS AND DEFINITIONS OF TERMS

Abbreviation	Definition
**ADA**	Anti-Drug Antibody
**ADCC**	Antibody Dependent Cellular Cytotoxicity
**AE**	Adverse Events
**ALT**	Alanine aminotransferase
**aPTT**	Activated partial thromboplastin time
**AST**	Aspartate aminotransferase
**AUC**	Area under the curve
**BC**	Breast cancer
**BEML**	Biomarkers and Experimental Medicine Leader
**BP**	Blood Pressure
**CA**	Competent Authority or Compartmental Analysis
**CEA**	Carcinoembryonic antigen
**CHF**	Congestive Heart Failure
**CL**	Clearance
C_{max}	Maximum serum concentration
C_t	Trough serum concentration
**CNS**	Central Nervous System
**CRO**	Contract research organization
**CT**	Computed Tomography
**CTCAE**	Common Terminology Criteria for Adverse Events
**%CV**	Coefficient of variation
**DCR**	Disease Control Rate
**DLT**	Dose-limiting Toxicities
**DNA**	Deoxyribonucleic acid
**DOR**	Duration of Response
**EC**	Ethics Committee
**ECG**	Electrocardiograms
**eCRF**	Electronic Case Report Form
**ECD**	Extracellular Domain
**EDC**	Electronic data capture
**EEA**	European Economic Area
**ESF**	Eligibility Screening Form
**EU**	European Commission
**FDA**	Food and Drug Administration

Abbreviation	Definition
**[18F]-FDG PET**	Fluoro Deoxy Glucose Positron Emission Tomography
**FFPE**	Formaldehyde fixed-paraffin-embedded
**HAHA**	Human anti-human antibodies
**HBV**	Hepatitis B
**HCV**	Hepatitis C
**HIV**	Human immunodeficiency virus
**IB**	Investigator's Brochure
**ICH**	International Conference on Harmonisation
**IEC**	Independent Ethics Committee
**IgA**	Immunoglobulin A
**IgE**	Immunoglobulin E
**IgG**	Immunoglobulin G
**IgM**	Immunoglobulin M
**IHC**	Immunohistochemistry
**IMP**	Investigational medicinal product
**IND**	Investigational New Drug (application)
**INR**	International normalized ratio
**IRB**	Institutional Review Board
**IRF**	Independent review facility
**IRC**	Independent Review Committee
**IRR**	Immune related response
**ISH**	In-situ hybridization
**IV**	Intravenous
**LDH**	Lactate dehydrogenase
**LPLV**	Last patient, last visit
**LSLO**	Last study subject, last observation
**LVEF**	Left ventricular ejection fraction
**MAD**	Multiple Ascending Doses
**MBC**	Metastatic breast cancer
**MD**	Multiple doses
**MRI**	Magnetic resonance imaging
**MTD**	Maximal tolerated dose
**NCA**	Non-compartmental analysis
**NCI**	National Cancer Institute
**NK**	Natural Killer
**NOAEL**	No observed adverse effect level

Abbreviation	Definition
**ORR**	Overall Response Rate
**OS**	Overall Survival
**PD**	Pharmacodynamic
**PET**	Positron Emission Tomography
**PFS**	Progression-free survival
**PK**	Pharmacokinetic
**PR**	Partial response
**PS**	Performance status
**PT**	Prothrombin time
**PTT**	Partial thromboplastin time
**QRS**	QRS Complex
**QT**	QT Interval
**q3w**	once every 3 weeks
**RBC**	Red Blood Cell
**RCR**	Roche Clinical Repository
**RECIST**	Response Evaluation Criteria in Solid Tumors
**RNA**	Ribonucleic acid
**RR**	RR Interval
**SAE**	Serious Adverse Event
**SD**	Standard Deviation
**SoA**	Schedule of Assessments
**SUSAR**	Suspected unexpected serious adverse reactions
$t_{½}$	Elimination half-life
**TQT**	Thorough QT
**TTE**	Transthoracic Echocardiogram
**ULN**	Upper limit of normal
**US**	United States
V_{ss}	Volume of distribution at steady state
**WBC**	White Blood Cell

1. BACKGROUND AND RATIONALE

1.1 BACKGROUND ON DISEASE

Breast cancer (BC) is the most common cancer in women, with a global prevalence of more than 1 million patients and an annual mortality rate of approximately 450,000 deaths (American Cancer Society). While improved early detection and advances in systemic therapy for early stage disease have resulted in a small decline in BC mortality since 1989, metastatic breast cancer (MBC) remains largely incurable with a median survival of approximately 24 months (National Cancer Institute, www.seer.cancer.gov). Treatment selection for MBC is guided by multiple factors, most importantly hormone receptor (HR) or human epidermal growth factor receptor 2 (HER2) expression, treatment history and prognostic factors such as short disease-free interval, presence of visceral metastases, performance status, and degree of symptoms.

1.2 BACKGROUND ON STUDY TREATMENTS

1.2.1 Background on RO5479599

Members of the erythroblastoma type B oncogene (ErbB) receptor tyrosine kinase family (also called human epidermal growth factor receptor [HER] family) have been shown to play a critical role in tumor growth, proliferation and progression in numerous epithelial malignancies. HER family receptors, while inactive as monomers, are known to form an interdependent, multilayered signaling network. Activation occurs following ligand binding leading to receptor dimerization. The resulting downstream signaling in tumor cells initiates diverse cellular pathways such as the Ras/Raf/mitogen-activated protein kinase (MAPK) and the phosphatidylinositol 3-kinase (PI3K)/v-akt murine thymoma viral oncogene homolog (Akt) pathways [1]. These signals regulate gene expression, cell survival and proliferation, angiogenesis and neoplastic growth. Targeted therapies for epidermal growth factor receptor (EGFR, also called HER1) such as erlotinib, gefitinib, cetuximab or panitumumab have demonstrated significant improvement of clinical outcome in epithelial malignancies expressing HER1 such as colorectal cancer (CRC) [2], squamous cell cancer of the head and neck (SCCHN) [3], carcinomas of the pancreas [4] and non-small-cell lung cancer (NSCLC) [5, 6].

In addition, targeting HER2, another family member of the HER family, with the HER2-specific monoclonal antibodies trastuzumab, pertuzumab or TDM-1 in HER2 overexpressing BC (defined as: IHC 3+ by immunohistochemistry [IHC] or positive defined by in-situ hybridization [ISH]) have demonstrated significant prolongation of overall survival (OS). HER2-targeted agents have been approved in the frontline and relapsed settings of HER2-positive disease [7, 8, 9, 10]. HER2 has no known ligand and, in a state of overexpression, can form active homodimers and initiate tyrosine kinase signaling without ligand stimulation. Additionally, as HER2 concentrations increase, the incidence of HER2 interactions with other receptors is also increased resulting in a broad recruitment of a number of intracellular signaling proteins [11]. Recent data obtained using micro-array technology suggest that the HER2 receptor can bind to more than 17

different proteins and may recruit proteins that other HER receptors cannot recruit. These activities highlight the promiscuity of HER2 in its ability to bind to other HER receptors and initiate tyrosine kinase signaling through several mechanisms [11].

HER3 is a key dimerization partner of HER family members and activates oncogenic signaling pathways. HER3 expression has been shown in many tumor types and overexpression of HER3 has been associated with poor prognosis [12] in BC [13, 14) as well as resistance to HER1- and HER2-targeted therapy [15, 16, 17]. HER3's kinase domain is inactive and thus it needs a dimerization partner like HER2 or HER1 for phosphorylation of its C-terminal tyrosine residues. Genetic dysregulation of HER3 is currently unknown. However, HER2-HER3 and HER1-HER3 heterodimers form potent dimeric HER signaling complexes [1]. Activation of the PI3K/Akt pathway is driven predominantly through phosphorylation of HER3 upon dimerization with HER1 or HER2, because six phosphorylation sites on HER3 can directly recruit p85, the regulatory subunit of PI3-kinase. Recent studies of HER3-expressing cancers indicate that this type of feedback and cross-talk between HER receptors contributes to the development of tumors in the lung, head and neck, breast, ovary, pancreas and stomach cancer among others [18]. High HER3 activity was found to be associated with resistance to anti-HER1/2 agents in lung cancer and BC cell lines. There are known mechanisms that allow HER3 phosphorylation to bypass anti-HER1/2 therapy, i.e. cross-signaling between HER3 and the tyrosine kinase receptor MET [16, 19, 15].

RO5479599 is a humanized, glycoengineered IgG1 antibody selectively binding with high affinity to the extracellular subdomain I of HER3. It is being developed by F. Hoffmann La Roche Ltd for the treatment of patients with malignant diseases that express the HER3 protein on the cell surface.

There are two distinct modes of action that are expected to contribute to the anti-tumor properties of RO5479599. The first is the inhibition of HER3-mediated signaling by blocking HER3 dimerization/phosphorylation and by inducing HER3 down-regulation from the cell surface. In addition, the Fc region of RO5479599 was glycoengineered to preferentially contain bisected non-fucosylated glycosylation variants. Such modified antibodies bind with higher affinity to low- and high-affinity variants of the FcγRIIIA receptors expressed on human immune effector cells such as natural killer (NK) cells, monocytes and macrophages. Increased binding affinity is expected to translate into higher antibody-dependent cellular cytotoxicity (ADCC) potency, i.e. more efficient killing of the antibody-targeted tumor cells by the immune effectors.

ADCC relies on effective interactions between the target cell (e.g. the tumor cell), the antibody and the effector cells (i.e. largely NK cells, monocytes/macrophages and polymorphonuclear leukocytes). NK cells mainly kill their target cells via releasing cytotoxic granules (i.e. perforin, granulysin and granzymes). The effective interaction between these three components requires receptors for the Fc portion of the IgG (FcγRs), which are encoded by eight genes on Chromosome 1. Three classes of FcγRs

(FcγRI, FcγRII, FcγRIII) have been identified and are differentially expressed on immune effector cells [20, 21]. NK cells express isoform A of FcγRIII (CD16a), while macrophages express FcγRI, IIA (activating), IIB (inhibitory) and IIIA. Genomic polymorphisms in these genes have been described. A specific polymorphism of the gene encoding FcγRIIIA resulting in the expression of either a valine (V) or a phenylalanine (F) at amino acid 158 has been reported to have an effect on clinical outcome of patients treated with antibody-based therapies. FcγRIIIA (V158) binds with greater affinity to the Fc part of the antibody than FcγRIIIA (F158). The higher binding affinity to the FcγRs on the surface of immune effector cells translates into increased ADCC activity [22]. In addition to ADCC, other antibody-dependent immune activation mechanisms mediated by NK cells and macrophages, e.g. antibody-dependent cellular phagocytosis and cytokine and chemokine secretion, may be enhanced by increasing the affinity of the Fc-FcγR interaction. Various studies have associated clinical efficacy with the FcγRIIIA-V158F variants, e.g. in follicular non-Hodgkin's lymphoma for rituximab (MabThera®) [20] and in BC for trastuzumab (Herceptin®) [21, 23].

See the RO5479599 Investigator's Brochure for details on non-clinical and clinical studies.

1.2.1.1 Previous Non-Clinical Studies

1.2.1.1.1 Non-Clinical Pharmacology

RO5479599 was shown to bind selectively and with high affinity (dissociation constant in the range of 1 nM) to the extracellular domain (ECD) of human and cynomolgus monkey HER3. In vitro binding of RO5479599 to HER3 inhibited (a) binding of the HER3 ligand heregulin (HRG), (b) HER3/HER1, HER3/HER2, and HER3/HER4 heterodimerization, (c) HER3 phosphorylation, (d) receptor downstream signaling via AKT and (e) cell proliferation, leading to cell death. In addition, RO5479599 binding to tumor cells in vitro resulted in down-modulation of the HER3 protein from the cell membrane.

Tumor growth inhibition and improved survival following treatment with RO5479599 were demonstrated in vivo in xenograft mouse models (including human lung, SCCHN, BC and pancreatic tumors). In vivo efficacy of RO5479599 was observed, EGFR and PI3K mutated but not in c-MET amplified xenograft models.

Treatment with RO5479599 as monotherapy resulted in significant tumor growth inhibition in multiple non-clinical models. Moreover, the combination of RO5479599 with HER1-targeted therapies such as erlotinib, cetuximab and GA201 (RO5083945) resulted in superior tumor growth inhibition in entities such as lung, SCCHN, CRC and pancreatic tumors and also tumor regression, in some cases up to complete tumor remission. In addition, the combination of RO5479599 plus pertuzumab has shown enhanced tumor growth inhibition and tumor regression in subcutaneous xenograft models of BC expressing HER3 and HER2 (HER2 amplified and non-amplified), ER+ and triple-negative BC models.

The effect of glycoengineering on the ADCC potency of the antibody was investigated both in vitro and in vivo.

RO5479599 showed enhanced induction of effector cell-mediated ADCC in cell-based assays compared to the non-glycoengineered variant of the antibody. The ability of RO5479599 to induce killing of target cells in vitro was shown to correlate with the level of HER3 expression on the cell surface.

In an orthotopic lung cancer xenograft model, mice treated with RO5479599 survived longer than those treated with vehicle or with the non-glycoengineered antibody.

For more detailed information, please refer to Non-Clinical Pharmacology Section of the RO5479599 Investigator's Brochure.

1.2.1.1.2 Non-Clinical Pharmacokinetics

After single intravenous (IV) administration in monkeys, RO5479599 showed a relatively low serum clearance (CL) and a volume of distribution at steady-state (V_{ss}) slightly lower than the plasma volume. Within the dose range tested (0.3-100 mg/kg), non-linear pharmacokinetics (PK) were observed with an extended half-life ($t_{1/2}$) at higher doses (mean $t_{1/2}$ approximately 4 days at 100 mg/kg compared to 1 day at 0.3 mg/kg). Following weekly IV administrations of 10, 25 or 50 mg/kg for 13 weeks, a slight accumulation (up to ~2-fold) of RO5479599 was observed in monkeys. Several monkeys across dose groups had reduced exposure following repeated dosing, probably due to the formation of anti-drug antibodies (ADAs). The presence of free ADAs was confirmed using a standard ADA assay in 1 out of 10 monkeys in the vehicle group and 9 out of 10, 7 out of 10 and 2 out of 10 in the 10, 25 and 50 mg/kg/week groups, respectively. An exploratory assay was developed to detect immune complexes of RO5479599 and ADAs. In this assay, all monkeys treated with RO5479599 were positive for immune complexes, with the exception of one high-dose male. The results of PK and PK/pharmacodynamic (PD) modeling and simulation support the starting dose in humans. For detailed information refer to Non-Clinical Pharmacokinetic Section in the RO5479599 Investigator's Brochure.

1.2.1.1.3 In Vitro Risk Assessment for Cytokine Release

RO5479599-induced cytokine secretion from human blood cells was investigated in vitro. In these experiments, whole blood samples from 25 healthy volunteers were incubated for 24 h together with RO5479599 at concentrations of 0.1 to 100 µg/mL. At concentrations of 100 µg/mL RO5479599, 72%, 60%, 32% and 20% of all donors were positive for cytokines IL6, IL8, TNFα and IFNγ, respectively. The median cytokine levels were up to 11.6-fold higher in the presence of RO5479599 than in the presence of cetuximab. RO5479599 had no cytotoxic effect on human blood leukocytes. The in vitro results suggest that the risk for the release of pro-inflammatory cytokines upon first administration of RO5479599 to humans is high. However, past experience with monoclonal antibodies that demonstrated a comparable risk in the whole blood assay

has shown that this risk could be effectively managed in the clinic with appropriate risk minimization measures. In the on-going phase 1 clinical Study BP27771 the incidence rate of IRRs upon first administration of RO5479599 was low and not accompanied by significant blood cytokine level increase and the severity of IRR signs and symptoms were mainly of grade 1 intensity (see Section 1.2.1.2.1).

1.2.1.1.4 Toxicology and Safety Pharmacology

A 13-week toxicity and toxicokinetic study with a 12-week recovery phase was conducted in cynomolgus monkeys. The main objective of this study was to provide non-clinical safety information to support long-term clinical studies in advanced cancer patients. The dose levels tested were 10, 25 and 50 mg/kg RO5479599, or vehicle, administered once weekly as IV bolus or short infusion. Safety pharmacology evaluations were performed as part of the 13-week study. There were no abnormal electrocardiogram (ECG), neurological or respiratory findings attributable to IV administration of RO5479599.

Starting between Week 2 and 4 of dosing, a large decrease of RO5479599 exposure was seen in 5 out of 10, 2 out of 10 and 1 out of 10 monkeys in the 10, 25 and 50 mg/kg dose group, respectively. This suggested marked ADA development in these monkeys in response to the humanized IgG antibody RO5479599. Significant decreases in RO5479599 exposure coincided with the detection of ADAs/RO5479599-ADA complexes and transient increases in IL6 and MCP1 post dose on Days 22 and 85. Since there was no direct cytokine release effect of RO5479599 in an in vitro monkey whole blood assay, the observed IL6 and MCP1 increases were considered to be due to immune complex-mediated complement activation.

Results from a C3a assay support the hypothesis that the observed hypersensitivity reactions in monkeys were the result of immune complex-mediated complement activation. C3a levels were measured in plasma samples. Increases of C3a levels (up to 227-fold from pre-dose values) were measured 30 min post-dose on day 36 of the 13-week study, but there was a lack of complement activation in an in vitro study indicating that RO5479599 did not directly activate the complement system. The increases of C3a levels in vivo were most likely the result of immune complex-mediated complement activation.

Adverse clinical signs, some coinciding with decreases in RO5479599 exposure levels, were first observed after the fourth weekly infusion, and their incidence and severity were highest in Weeks 4 and 5. Most adverse clinical signs had resolved by Week 8. The clinical signs in the animals (4 out of 10, 2 out of 10 and 1 out of 10 in the 10, 25 and 50 mg/kg dose group, respectively) were consistent with acute hypersensitivity reactions (e.g. emesis, dilated pupils, decreased consciousness, pale mucous membranes) that occurred within 5 to 10 min following drug administration on Days 22, 29, 36, 43 and/or 50. In addition, 2-h post-dose observations included one or more of the following (mostly in animals dosed at 25 and 50 mg/kg): disuse of hands or feet (finger

and toe curling), poor coordination, tremors, watery feces, emesis, decreased activity, and/or pale mucous membranes. These symptoms generally resolved within 24 h. Two of these monkeys had to be euthanized after the fourth infusion due to deteriorating physical condition following initial hypersensitivity reactions. These two monkeys showed marked decreases in platelet counts, minimally prolonged activated partial thromboplastin time (aPTT), and a lower erythrocyte mass associated with petechial hemorrhages on legs and arms and bleeding from gums and nasal mucosa. The histopathological exam showed fibrin and fibrin thrombi in multiple organs. Markedly decreased albumin levels resulting in moderate decreases in total protein levels and albumin/globulin ratio and minimally increased fibrinogen levels were suggestive of an acute phase reaction. Additional findings in these monkeys may have been the result of cytokine stimulation; a relatively high number of immature myeloids in the bone marrow, accumulation of immature myeloid and erythroid precursor cells in the vasculature and parenchyma in multiple organs, and changes in neutrophils that suggested premature transit of these cells from the bone marrow.

Both the acute and 2-h post-dose clinical signs observed in monkeys were considered immune complex-mediated hypersensitivity reactions. This hypothesis is supported by the following observations in these monkeys:

- High response rate of ADAs and/or immune complexes
- Increased C3a levels in some monkeys
- Lack of exposure-effect relationship
- Lack of increases in incidence or severity of the reactions over time
- Lack of a correlation with known pharmacological effects of HER3 signaling inhibition or ADCC of RO5479599

Appropriate risk-minimization measures for potential immunogenicity-related toxicities as well as for potential infusion-related reactions (IRRs) will be implemented in this study as described in Section 1.3.3 and Section 4.4.2.6.

Apart from the aforementioned immune complex-mediated toxicities, no adverse findings could be found that could be attributed to HER3 signaling inhibition or ADCC of RO5479599. The development of ADAs to RO5479599, a humanized IgG antibody, is an expected immune response in monkeys, however, is unlikely to occur in humans (ICH S6 Note for guidance on non-clinical safety evaluation of biotechnology-derived pharmaceuticals [CHMP/ICH/302/95], November 2009; ICH S6 (R1): Addendum to ICH S6, May 2011).

In the monkeys that remained in the study until the scheduled necropsy, the only observed changes were transient decreases in albumin and albumin/globulin ratio, and increases in globulin observed in a few individual monkeys in all dose groups on Days 28 and 34. Globulin levels returned to their pre-study level at the end of the 13 week

treatment phase (Day 91). There were no changes in hematology parameters, gross or microscopic findings associated with administration of RO5479599.

As part of the study, an exploratory time course evaluation of circulating NK cells in relation to the RO5479599 infusion was performed by flow cytometry. Significant decreases in levels of circulating NK cells were observed 4 h post-dose compared to pre-dose values. These changes were seen in all groups, including the vehicle control group. The values returned to pre dose levels within 24 h post-dose. These transient decreases of circulating NK cells were not considered toxicologically meaningful.

1.2.1.2 Previous Clinical Studies

1.2.1.2.1 Clinical Safety and Tolerability

At the time of protocol writing, the clinical experience with RO5479599 is limited to phase I data as monotherapy and in combination with EGFR-targeted therapies, i.e. erlotinib and cetuximab from Study BP27771. As of 8th of February 2013, 25 patients have been enrolled in 6 cohorts and treated with a q2w regimen with RO5479599 monotherapy from 100 to 2000 mg doses. No DLTs were observed. Patients had a median (range) of 3 (2 to 6) prior chemotherapy lines.

In total, there were 208 AEs reported in 25 patients with all patients in the study reporting at least one AE. Among the 208 AEs, 14% (28/208) AEs were reported in 3 out of 3 patients in Cohort 1 (100 mg), 11% (22/208) AEs in 3 out of 3 patients in Cohort 2 (200 mg), 11% (23/207) AEs in 3 out of 3 patients in Cohort 3 (400 mg), 27% (57/207) AEs in 7 out of 7 patients in Cohort 4 (800 mg), 15% (30/207) AEs in 5 out of 5 patients in Cohort 5 (1600 mg) and 23% (48/207) AEs in 4 out of 4 patients in Cohort 6 (2000 mg). Most of the AEs were grade 1 (65%) and grade 2 (26%) in intensity. The most frequently reported adverse events (in more than 5 patients) are diarrhea in 48% of patients (12/25 patients), fatigue in 44% of patients (11/25 patients), decreased appetite and IRR in 32% of patients (8/25 patients), constipation in 24% of patients (6/25 patients), and nausea, pyrexia and stomatitis in 24% of patients (6/25 patients). Thirteen out of the 25 patient enrolled in the study (52%) experienced 19 grade 3 events out of 207 events (9% of all AEs). These events were anemia, ascites, chest pain, dyspnea, fatigue (2x), infectious peritonitis, hypertension, laryngeal obstruction, pyrexia, anal abscess, IRR, blood AST potassium and GGT (2x) increased, blood phosphorous and potassium decreased. Only 1 patient (4%) experienced a life-threatening event (general physical health deterioration) in Cohort 1. The number of AEs and the associated severity did not appear to be dose-dependent, however, the number of patients is limited until the time of data cut-off. Ten IRRs of grade 1 to 3 occurred in 8 patients. Three study drug-related AEs grade 3 were reported, 1 IRR, 1 GGT increased and 1 neutropenia. Two patients in Cohort 4 (800 mg) were withdrawn from the study due to re-occurrence of infusion-mediated signs and symptoms (IRR of grade 2 and 3, respectively) after rechallenge with RO5479599 infusion. Only 1 patient tested positive for HAHA so far, who was withdrawn from the study due to IRR. There were 4 deaths during the study period, none of which was considered related to study treatment.

Eleven and 9 patients have been treated with RO5479599 plus cetuximab and RO5479599 plus erlotinib, respectively. There was 1 DLT in an 81 year-old CRC patient treated with RO5479599 plus cetuximab due to grade 3 diarrhea and dehydration leading to hospitalization. Overall, the AEs in the EGFR-targeted combination arm are mostly due to EGFR inhibitor-related toxicities and there is currently no evidence that the combined inhibition of HER3 and HER1 would exaggerate EGFR inhibitor-related toxicities such as skin rash (64% of patients in the RO5479599 plus cetuximab group and 60% of patients in the RO5479599 plus erlotinib group), diarrhea (50% of patients in the RO5479599 plus cetuximab group and 70% of patients in the RO5479599 plus erlotinib group) or hypomagnesemia (29% of patients in the RO5479599 plus cetuximab group and 10% of patients in the RO5479599 plus erlotinib group).

Overall, RO5479599 was well tolerated as monotherapy up to 2000 mg and in the ongoing dose escalation phase in combination with EGFR-inhibition therapy and demonstrated preliminary signs of clinical activity.

1.2.1.2.2 Clinical Pharmacokinetics and Pharmacodynamics

In the monotherapy phase I dose escalation part of Study BP27771, RO5479599 doses from 100 mg to 2000 mg were investigated. Based upon a preliminary PK analysis of the phase I monotherapy dose escalation (n = 25 patients), the PK of RO5479599 was shown to be non-linear from 100 mg up to 400 mg. Both C_{max} and AUC showed a greater than dose proportional increase, accompanied by a decline in clearance over the same dose range indicating that the elimination of RO5479599 across this dose range is predominantly target-mediated. For doses above 400 mg exposure increased approximately dose-proportionally and little change in total clearance was seen. Preliminary geomean (%CV) PK parameters of RO5479599 at Cycles 1 following q2w administration are presented in Table 1.

Table 1 Phase I Study BP27771: GeoMean (%CV) RO5479599 PK Parameters During Cycle 1 in HER3+ Advanced/Metastatic Solid Tumor Patients Following Ascending Doses from 100 mg to 2000 mg q2w

Dose [mg] (No. of patients)	C_{max} (µg/mL)	AUC_{last} (µg*day/mL)	AUC_{7d} (µg*day/mL)	Total CL (mL/day)
	GeoMean (%CV)			
**100 (n=3)**	25.4 (26.8)	89.2 (27.4)	75.6 (12.9)	1030 (19.5)
**200 (n=3)**	85.2 (22.0)	310 (17.9)	231 (17.4)	579 (19.1)
**400 (n=3)**	208 (32.8)	1080 (18.0)	717 (20.8)	242 (16.2)
**800 (n=7)**	426 (27.7)	1620 (52.2)	1200 (46.3)	378 (45.4)
**1600 (n=5)**	648 (20.8)	3450 (32.7)	2150 (32.1)	359 (23.1)
**2000 (n=4)**	655 (43.1)	3570 (42.1)	2720 (32.5)	324 (34.9)

HER3 membraneous protein downregulation was observed in skin from the 100 mg dose onwards. On-treatment tumor biopsy was only implemented starting from the 200 mg dose cohort and down-regulation of HER3 membraneous protein was seen from the 200 mg dose onwards. Five patients (1 NSCLC, 2 CRC, 1 SCCHN, 1 BC) had a best response of stable disease (SD). Confirmed SD (>16 weeks) was observed in 2 patients. One BC patient achieved a PR in ^{18}FDG-PET and significant shrinkage of non-target tumor lesions in CT scan.

1.2.2 Background on Pertuzumab

Pertuzumab is a fully humanized monoclonal antibody based on the human IgG1(κ) framework sequences and consisting of two heavy chains (449 residues) and two light chains (214 residues). Similar to trastuzumab, pertuzumab is directed against the extracellular domain of HER2. However, it differs from trastuzumab in the epitope-binding regions of the light chain (12 amino acid differences) and heavy chain (29 amino acid differences). As a result, pertuzumab binds to an epitope within what is known as sub-domain 2 of HER2, while the epitope for trastuzumab is localized to sub-domain 4 [24, 25].

Pertuzumab acts by blocking the dimerization of HER2 with other HER family members, including EGFR, HER3 and HER4. As a result, pertuzumab inhibits ligand-initiated intracellular signaling through two major signal pathways, MAP kinase and PI3 kinase. Inhibition of these signaling pathways can result in growth arrest and apoptosis, respectively [26, 27]. Data from a clinical trial could show that MBC patients who

progressed under trastuzumab therapy clinically responded again when treated with a combination of trastuzumab and pertuzumab [26, 27, 28]. The results obtained suggest that a more comprehensive blockade of HER2 through interruption of heterodimerization may provide clinical benefit.

Due to the different binding sites of pertuzumab and trastuzumab, ligand-activated downstream signaling is blocked by pertuzumab, but not by trastuzumab. Due to their complementary modes of action, there is a potential role for the combination of pertuzumab and trastuzumab in HER2-overexpressing diseases.

The phase III, randomized, double-blind, placebo-controlled study “Clinical Evaluation of Pertuzumab and Trastuzumab” (CLEOPATRA) assessed the efficacy and safety of the combination of pertuzumab and trastuzumab with docetaxel as first-line treatment for patients with HER2 IHC3+ or ISH+ MBC. The study established that targeting HER2-positive tumors with two anti-HER2 monoclonal antibodies that have complementary mechanisms of action along with chemotherapy, as compared with placebo plus trastuzumab plus docetaxel, resulted in a significant reduction in the risk of progression or death (hazard ratio: 0.62) and an increase of 6.1 months in median progression-free survival (PFS). The combination therapy with pertuzumab did not increase the rates of symptomatic or asymptomatic cardiac dysfunction. AEs (any grade) of diarrhea, rash, mucosal inflammation, febrile neutropenia and dry skin were reported more frequently in the pertuzumab group than in the control group [30].

Pertuzumab will be administered at a loading dose of 840 mg during Cycle 1 followed by 420 mg for the remaining cycles in a q3w schedule. This dose and schedule has been evaluated in monotherapy and in combination with a variety of chemotherapy agents and a variety of tumor entities and has been demonstrated to be safe. Clinical efficacy of this dose and schedule of pertuzumab has been demonstrated in HER2-overexpressing BC when combined with taxanes and trastuzumab [9, 29, 30].

See the Pertuzumab SmPC for details on non-clinical and clinical studies.

1.2.3 Background on Paclitaxel

Taxanes are anti-neoplastic agents that bind to free tubulin within the cell and promote the assembly of tubulin into stable microtubules while simultaneously inhibiting their disassembly. This mode of action leads to the production of microtubule bundles without normal function and to the stabilization of microtubules, blocking cells in the M-phase of the cell cycle and leading to cell death. Extensive phase II and III data have led to regulatory approvals for its use either in combination or as monotherapy for the treatment of BC.

Early clinical studies of conventional paclitaxel showed that it is associated with significant toxicity, including peripheral neuropathy, mucositis, arthralgia/myalgia and myelosuppression (primarily neutropenia) which is dose limiting [31]. Hypersensitivity

reactions [31] against polyoxyethylated castor oil (Cremophor EL) which is used as a solvent in the formulation of paclitaxel [32] led to the prophylactic use of antihistamines and corticosteroids, and longer infusion times to limit these toxicities. Paclitaxel administered initially at 175 mg/m^2 as 3-h IV infusion once every three weeks (q3w) with pre-medication was associated with significant clinical benefits in women with MBC [33] and became the widely adopted monotherapy regimen. However, the evaluation of weekly dosing regimens of paclitaxel has demonstrated that qw dosing of paclitaxel is superior to q3w dosing in MBC and early BC [34, 35, 36, 37, 38] and these regimens have also been included in treatment guidelines [39, 40, 41].

The most common grade 3 toxicities to paclitaxel qw therapy have been neuropathy (~20%), IRRs (3%), neutropenia (4%), febrile neutropenia (2%), infection (3%), alopecia (~40%) and fatigue (~6%) [37, 43, 36].

See the local prescribing information for paclitaxel for details on non-clinical and clinical studies.

1.3 STUDY RATIONALE AND BENEFIT–RISK ASSESSMENT

1.3.1 Study Rationale

Insight into molecular cancer biology has enabled significant advances in cancer diagnostics and therapy. However, for the vast majority of patients suffering from advanced and/or metastatic solid cancer the disease remains incurable. Although the treatment intent is palliative in the metastatic setting there is a huge need for improvement of OS.

The advent of HER2 testing and HER2-targeted agents for tumors overexpressing HER2 (i.e. IHC 3+) or having HER2 gene amplification assessed by ISH (ISH+) has been associated with a transformational improvement of clinical outcome in these patients. However, the majority of BC tumors (approximately 75%) do not show overexpression of the HER2 protein (IHC3+) or HER2 gene amplification (ISH+). For these patients in need of systemic therapy for MBC the options are limited. Anthracycline- or taxane-based chemotherapy is indicated as initial therapy for patients with HR-negative disease and following failure of hormonal therapies in HR-positive disease. Targeted therapies are currently not established as standard of care therapy for these patients emphasizing the significant need for new agents with novel mechanisms of action which can be combined with established treatment. In randomized, controlled, phase III trials in patients with predominantly HER2-negative metastatic or locally recurrent BC, the addition of bevacizumab to paclitaxel or capecitabine prolonged PFS relative to paclitaxel or capecitabine alone. It also significantly increased the objective response rate (ORR), but not OS. However, in confirmatory trials the PFS benefit of bevacizumab was rather small [43]. Therefore, bevacizumab approval in MBC was withdrawn in the U.S. by the FDA. The addition of bevacizumab to taxane-based chemotherapy in MBC is therefore not considered standard of care but may be an option for selected patients [41, 40].

The combination of RO5479599 plus pertuzumab has shown additive tumor growth inhibition and tumor regression in subcutaneous xenograft models of BC expressing HER3 and HER2 (HER2-amplified and non-amplified), ER+ and triple-negative BC models. The anti-tumor efficacy of RO5479599 plus pertuzumab was superior to the single agents and superior to the antitumor efficacy of RO5479599 plus trastuzumab supporting the concept that comprehensive inhibition of HER family heterodimers confers additive antitumor efficacy. This concept is further supported by the clinical efficacy of pertuzumab that is highly significant in HER2 overexpressing BC when combined with trastuzumab [9, 29, 30] but modest when used as monotherapy in HER2 non-overexpressing BC [44].

In contrast to previous studies testing the combination of trastuzumab and pertuzumab, the present study will be performed in patients whose BC does not overexpress HER2. Growth of such tumors expressing “normal” levels of HER2 may not be driven by HER2 homodimers but rather different heterodimers may be formed among HER family receptors such as HER2:HER3, HER2:HER1 and HER1:HER3. Combination treatment with pertuzumab and RO5479599 is expected to inhibit all of these heterodimers and thereby provide a more comprehensive blockade of HER family signaling. Furthermore, enhancement of ADCC by glycoengineering of RO5479599 may particularly contribute immune-mediated therapeutic effects on tumors with normal levels of HER2 expression.

Preliminary efficacy of RO5479599 was observed by achieving a partial metabolic response and tumor shrinkage at 1600 mg of monotherapy in a patient with triple-negative MBC expressing HER3 protein.

Paclitaxel (qw) will be included as chemotherapy backbone as it is considered an efficacious and well tolerated standard of care chemotherapy regimen for the treatment of MBC and should, therefore, provide clinical benefit independent of pertuzumab and RO5479599.

As the combination of RO5479599 has not been clinically tested in combination with paclitaxel and pertuzumab the study will be designed with a dose escalation phase followed by an extension phase for the highest dose reached. In an additional extension phase cohort the safety, tolerability and preliminary efficacy in patients with no prior chemotherapy for metastatic disease and/or a maximum of one prior chemotherapy regimen in the adjuvant or neoadjuvant setting will be assessed in order to support a potential future development of this combination in early breast cancer. Standard doses of paclitaxel and pertuzumab will be administered with escalating doses of RO5479599. The decision to move into the extension phase will be based on the safety data generated in the dose escalation phase.

1.3.2 Rationale for Patient Selection Based on the HER3 & HER2 Protein Expression and HER2 Gene Amplification Status

Tumor growth inhibition by RO5479599 was demonstrated in vivo in subcutaneous mouse xenograft models representing different tumor entities (e.g. lung, SCCHN, BC and pancreas). Treatment with the anti-HER3 antibody also resulted in inhibition of HER3 phosphorylation and down-modulation of HER3 protein levels in the tissue.

A high ratio of phosphorylated HER3 (pHER3) to HER3 levels strongly correlated to response to the anti-HER3 antibody. Non-clinical tumor models lacking HER3 protein expression consistently did not demonstrate any response to RO5479599 across different tumor entities. In addition, HER3 protein expression is a requirement for RO5479599 to bind to the target with the Fab part on the one side and bind to the immune effector cell with the Fc part to initiate ADCC.

The presence of HER3 protein on the tumor cell surface is a prerequisite to evaluate PD effects after RO5479599 administration. In addition, excluding patients who do not demonstrate HER3 protein expression on their tumors will prevent unnecessary exposure of patients to an ineffective targeted therapy. Prevalence data, which were generated by measuring HER3 protein expression using IHC, demonstrated that HER3 is expressed broadly in several human primary tumors (e.g. NSCLC, HNSCC, gastric cancer or pancreatic cancer).

A robust prototype assay will be provided by Ventana Medical Systems (VMS) which allows screening for patients whose tumor sample is positive for HER3 protein expression by IHC. A representative tumor specimen should contain at least 100 viable neoplastic cells and discernible HER3 membrane positivity in any neoplastic cell will be considered diagnostically positive for HER3 protein expression.

Pertuzumab is registered in the combination with trastuzumab and docetaxel for HER2-positive BC defined as IHC 3+ or ISH+.

HER2-normal expressing tumors are supposed to preferably form HER2-HER3 heterodimers, in contrast to HER2 overexpressing tumors, which can preferably form HER2-homodimers due to high receptor density. It has been described that HER2-HER3 heterodimers can act as very potent and highly regulated signaling transduction partners and thereby promote tumor growth and tumor cell survival.

Hence, only patients will be included whose tumors represent a staining pattern referring to HER2 IHC 1+/ISH- or HER2 IHC 2+/ISH- as described in the respective assay working procedure. For assessing the HER2 status an FDA-approved test will be used for both IHC and ISH.

Diagnostic assessment of HER2 and HER3 has to be conducted in parallel for identifying the target patient population.

1.3.3 Risk-Benefit Assessment

The majority of epithelial cell-derived tumors have a complex genetic background and there is significant compensatory crosstalk between the various HER receptors as well as with other pathways regulating cell growth, proliferation, migration and survival. Rationally developed biologically targeted combinations will likely enhance the benefit of single agents to the treatment of these cancers.

Non-clinical experiments have shown that combined inhibition of HER3 and HER2 by pertuzumab and RO5479599 confers additive anti-tumor efficacy as compared to the single agents in human BC models expressing HER3 protein and “low to intermediate” levels of HER2 protein including ER+ and ER- models. These data support the concept that comprehensive inhibition of HER family dimerization may confer enhanced clinical efficacy.

In addition, pertuzumab and RO5479599 will be combined with paclitaxel, a widely used standard chemotherapy agent in MBC to provide efficacy independent of HER2/HER3 inhibition at manageable side effects.

As mentioned before, there is currently no clinical experience with RO5479599 in combination with pertuzumab and paclitaxel. The combination of monoclonal antibodies and classical chemotherapy agents is usually safe and tolerable. In most case where improved efficacy is associated with increased toxicities of the combined chemo-immuno-therapy agents compared to chemotherapy alone, these toxicities have been manageable. Examples include bevacizumab [43], rituximab [45], cetuximab [3], pertuzumab and trastuzumab [9]. Preliminary reports of alternative anti-HER3 antibodies in combination with paclitaxel are available. Liu et al. reported on 28 patients with gynecological tumors including BC treated with paclitaxel and MM-121, a monoclonal antibody against HER3 [46]. In this study paclitaxel was given weekly at 80 mg/m^2 and MM-121 was given weekly at doses from 10 mg/kg to 40 mg/kg. The combination was regarded safe with toxicities comparable to those expected with weekly paclitaxel alone. An MTD was not reached. The most common ≥ grade 3 events included fatigue (14%), neutropenia (14%), mucosal inflammation (11%), small intestine obstruction (11%), anemia (7%), diarrhea (7%) and neuropathy (7%). Richards et al., reported on 15 patients treated with a bispecific anti-HER2-anti-HER3 antibody (MM-111) in combination with trastuzumab and paclitaxel weekly. Again the combination was found to be well tolerated up to doses of 20 mg/kg MM-111 weekly. The safety profile of MM-111/trastuzumab/paclitaxel appeared to be consistent with that of the trastuzumab/paclitaxel backbone [47].

The combination of pertuzumab plus various chemotherapy agents including paclitaxel or docetaxel but also anthracyclines, carboplatinum and gemcitabine was well tolerated in patients with ovarian cancer [48, 49] and BC [9, 29, 50] and other tumor entities [51, 52].

Based on the safety and efficacy data that have been generated with paclitaxel and pertuzumab and RO5479599 thus far, the biology of HER3 as described above and the non-clinical efficacy of the clinical combination of RO5479599 and pertuzumab plus paclitaxel may confer greater clinical benefit than the therapy with single agents and warrant early clinical testing. Appropriate measures to monitor and prevent toxicities arising from taxanes and/or the combination with HER2- and HER3-targeted therapy have been implemented into this trial.

The expected toxicities of the combination of paclitaxel plus pertuzumab and RO5479599 may include:

Neuropathy ≥ grade 3 has been reported in up to 20% of patients treated with paclitaxel. Appropriate measures for prevention, monitoring and treatment are in place including exclusion criteria, clinical monitoring and examination, dose reductions and delays. The management of dose delays and dose modifications of neuropathy are described in Section 4.4.2.5.

Diarrhea mostly grade 1 and 2 has been reported in 60 to 70% of patients treated with pertuzumab. The incidence of grade 3 diarrhea in patients treated with chemotherapy, trastuzumab and pertuzumab was 6 to 8% (Baselga et al. 2012, Gianni et al. 2012). About 48% of patients had grade 1 diarrhea and 4% of patients grade 2 diarrhea when treated with RO5479599 monotherapy. No patients experienced grade 3 or 4 diarrhea. Appropriate measures for prevention, monitoring and treatment are in place including clinical monitoring and examination, dose reductions and delays and symptomatic treatment (i.e. loperamide and fluids). The management of dose delays and dose modifications of diarrhea are described in Section 4.4.2.5.

Hematological toxicities: Neutropenia ≥ grade 3 has been reported in up to 20% of patients treated with paclitaxel. Febrile neutropenia has been reported in up to 20% of patients treated with paclitaxel. Appropriate measures for prevention, monitoring and treatment are in place including exclusion criteria, clinical monitoring and blood sampling, dose reductions and delays as well as symptomatic therapy (e.g., growth factors). The management of dose delays and dose modifications of hematological toxicities are described in Section 4.4.2.5.

IRRs of grade 3 have been reported in only 1 out of 25 patients treated with RO5479599 monotherapy (4%) in the ongoing phase I Study BP27771. Grade 3 and 4 IRRs have been rarely reported with pertuzumab (<1% of patients). Symptoms usually occur during the first infusion and up to 1 h after the end of infusion, but may occur up to 24 h after the end of the infusion and also with subsequent infusions. Pre-medication for both RO5479599 and pertuzumab include paracetamol and antihistamines. Pre-medication for paclitaxel includes corticosteroids to minimize the risk of hypersensitivity reactions. It is recommended to warn patients of the possibility of a late onset of IRRs and instruct them to contact their physician if symptoms of an IRR occur. Occurrence of a severe

IRR requires immediate interruption of pertuzumab and RO5479599 infusion and may necessitate emergency treatment. Subsequent administration of pertuzumab and RO5479599 will only occur after resolution of symptoms to grade ≤ 1. Special attention is recommended for patients with reduced performance status and pre-existing cardio-pulmonary disease.

Rash: Mild to moderate rash has been observed in 17% of patients treated with pertuzumab in phase II and in 2 out of 25 patients (8%) of patients treated with RO5479599 monotherapy in the currently running phase I Study BP27771. Patients will be monitored closely for rash and will be treated accordingly.

Immunogenicity: Continuous monitoring of immunogenicity will be performed consisting of continuous assessment of platelets, hematuria, HAHA and RO5479599 exposure and clinical signs of hypersensitivity reactions (rash, arthropathies). In patients with clinical signs of hypersensitivity reactions or immune complex-mediated toxicities, treatment will be immediately stopped and appropriate diagnostic measurements will be performed. Patients with confirmed HAHA formation will be removed from the study. For patients in whom HAHA formation could not be confirmed, the decision to remove the patient will be determined between the investigator and the sponsor (see Section 4.7.1). Only 1 out of 25 patients was positive for HAHA with RO5479599 monotherapy in the ongoing phase 1 Study BP27771. This patient in the 800-mg cohort experienced one episode of grade 3 IRR at Cycle 2 and upon rechallenge with RO5479599 on the next day a grade 2 IRR. The patient was consequently withdrawn from the study.

RO5479599 must be administered in a hospital or clinic equipped for IV chemotherapy. Full emergency resuscitation facilities should be immediately available and patients should be under close supervision of the investigator at all times. Adequate treatment of IRRs will be provided according to clinical symptoms (as described in Section 4.4.2.3.2).

Cardiac toxicity: To date there are no signs of cardiac toxicity associated with RO5479599 treatment in Study BP27771 or in animal toxicity studies. HER3 has not been shown to be expressed in the heart. In addition, in CLEOPATRA, the combination of pertuzumab with trastuzumab and docetaxel did not result in an increase in left ventricular systolic dysfunction (LVSD) as compared to the control group. Nonetheless, appropriate measures for prevention and monitoring are in place including exclusion criteria, clinical monitoring, ECG and echocardiography. All patients enrolled in pertuzumab studies undergo regular LVEF monitoring by echocardiography.

Patients with significant cardiac disease or baseline LVEF below 50% are not eligible for this study. Risk factors for pertuzumab-associated cardiac dysfunction are not known at this time, and this risk should be carefully weighed against the potential benefit in patients who have received prior anthracyclines. During the screening/baseline period, complete medical history information will be collected from all patients to explore possible risk factors for treatment (e.g. CHF), including all prior LVEF assessments.

Monitoring of LVEF is required while patients are receiving study treatment and will be analyzed centrally. If symptomatic LVSD develops (NCI-CTCAE version 4.0 grade 3 or 4) with a drop in LVEF consistent with cardiac failure, the patient must discontinue study treatment. LVSD, whether symptomatic or not, should be treated and followed according to standard medical practice.

In summary, patients with BC are in continuous need of improved therapy options. This is especially true for BC that does not overexpress HER2 such as the patient population that will be eligible for this trial. Non-clinical data have demonstrated promising anti-tumor efficacy of RO5479599 in combination with pertuzumab in xenograft models including triple-negative BC models. A number of clinically appropriate strategies to minimize risk to patients have been built into the protocol through the means of inclusion/exclusion criteria, monitoring strategies and management guidelines. Furthermore, safety data from the dose escalation phase will be available before extending the cohorts or initiating a cohort with a higher dose level of RO5479599.

Overall, the potential benefits of combined HER3- and HER2-targeted therapy in combination with an efficacious, well tolerated and widely used chemotherapy regimen of weekly paclitaxel for MBC patients outweigh the risks of RO5479599, pertuzumab and paclitaxel combination treatment proposed for this phase 1b study.

2. OBJECTIVES

2.1 PRIMARY OBJECTIVES

The primary objectives of this study are:

- To describe the safety profile and PK of selected doses of RO5479599 in combination with pertuzumab and paclitaxel in patients with MBC expressing HER2 and HER3 protein.
- To determine the recommended phase II dose(s) (RPTD) and schedule(s) for RO5479599 in combination with pertuzumab and paclitaxel.

2.2 SECONDARY OBJECTIVES

The secondary objectives for this study are:

- To describe the preliminary anti-tumor activity of RO5479599 in combination with pertuzumab and paclitaxel by assessing objective response rate (ORR, including patients with confirmed complete response [CR] or confirmed partial response [PR]), disease control rate (DCR, including patients with an objective response or stable disease [SD]) and PFS.
- To describe the PD effects of RO5479599 in combination with pertuzumab and paclitaxel.

2.3 EXPLORATORY OBJECTIVES

The exploratory objectives for this study are as follows:

- To describe the relationship between PK and PD results.
- To describe the relationship between PK and clinical outcomes.
- To explore the association between response prediction biomarkers and clinical outcome.

3. STUDY DESIGN

3.1 DESCRIPTION OF STUDY

This is an open-label, multicenter, phase Ib clinical study to investigate the safety profile and PK of selected doses of RO5479599 in combination with pertuzumab and paclitaxel in patients with MBC expressing HER3 and HER2 protein. The combination treatment of RO5479599, pertuzumab and paclitaxel will be referred to as study treatment in the following sections of the protocol.

3.1.1 Overview of Study Design

The study design is shown in Figure 1.

Figure 1 Study Design

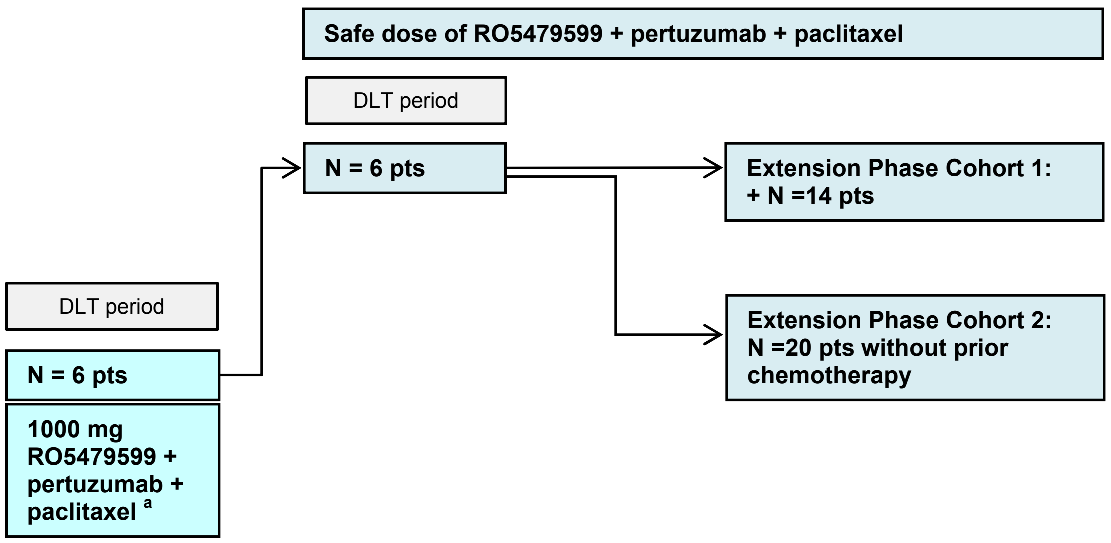

[a] Depending on the toxicity data generated for the 2000-mg cohort there will be the option to also expand the 1000 mg cohort to up to 12 to 20 patients in total.

In the dose escalation phase of RO5479599 in combination with pertuzumab and paclitaxel, 6 patients will be enrolled in each of the selected RO5479599 dose cohorts (1000 and 2000 mg) with a one-week window between the first two patients. At first, the 1000 mg dose will be tested for safety. If the first 6 evaluable patients enrolled in the 1000 mg cohort of RO5479599 fulfill the safety criteria as defined in Section 3.2.2 escalation to the 2000 mg cohort of RO5479599 in combination with pertuzumab and paclitaxel will be initiated. If the first 6 evaluable patients enrolled in the 2000 mg cohort of RO5479599 fulfill the safety criteria as defined in Section 3.2.2 the 2000 mg cohort will be expanded to a total of 20 patients (Extension Phase Cohort 1). Depending on the toxicity data generated in the 2000 mg cohort or should additional patients need to be enrolled in the 1000 mg cohort to allow a safety comparison between cohorts there is the option to also expand the 1000 mg cohort to a total of 12 to 20 patients. Should the 2000 mg cohort not fulfill the safety criteria as defined in Section 3.2.2 the respective cohort will not be completed. Should the 1000 mg cohort not fulfill the safety criteria as defined in Section 3.2.2 the respective cohort will not be expanded and the 2000 mg dose level will not be initiated. Instead lower dose level**s** of RO5479599 may be evaluated following the same rules as described in Section 3.2.2.

The patients in the dose escalation phase for doses of 1000 and 2000 mg will be followed up for 21 days after commencement of treatment with RO5479599 in combination with pertuzumab and paclitaxel. Only at the end of this DLT period i.e., after the first 6 evaluable patients have completed one cycle, more patients can be recruited in each cohort.

After determination of a safe dose of RO5479599 in combination with pertuzumab and paclitaxel, another extension phase cohort with approximately 20 MBC patients will be recruited (Extension Phase Cohort 2). Only patients with no prior chemotherapy for metastatic disease and/or a maximum of only one prior chemotherapy regimen in adjuvant or neoadjuvant setting will be selected.

In order to explore possible improvements of the safety profile of the combination treatment (especially regarding diarrhea) the Extension Phase Cohort 2 will commence at a reduced dose of pertuzumab, i.e. 420 mg for Cycle 1 followed by 420 mg for subsequent cycles. The first 6 patients will be observed for the incidence and intensity of diarrhea for 2 cycles before expanding the cohort to 20 patients. If 0 or 1 out of the first 6 evaluable patients experiences a Grade ≥3 diarrhea (as defined for DLTs in Section 3.2.2.1), then further recruitment in this cohort will be initiated to a total of 20 patients. If 2 out of 6 patients experience a Grade ≥3 diarrhea, recruitment will only be continued after the sponsor and investigators have reviewed and concluded that the severity and the type of the diarrhea is medically acceptable. If 3 or more out of 6 patients experience a Grade ≥3 diarrhea then enrollment is stopped at this pertuzumab and RO5479599 dose. Dependent on the outcome of this safety cohort a further dose modification of pertuzumab and/or RO5479599 based on safety and exposure data generated may

then be implemented and evaluated in an additional safety cohort of n=6 patients plus extension phase cohort following the same decision making criteria as described above.

3.1.2 End of Study

All patients will attend a safety follow-up visit 28 days after receiving the last infusion of study treatment. Another PK/HAHA visit on Day 42 to 45 will be conducted for patients who are not entering another clinical trial or receiving further anti-tumor treatment. In the event of early discontinuation from the treatment, patients will also undergo a safety follow-up visit 28 days after receiving the last infusion of study treatment. This follow-up visit after early discontinuation can be brought forward to an earlier time point at the discretion of the investigator. The patient's survival status will be followed up by regular phone calls for up to 2 years after discontinuation from the study. Patients will be treated until disease progression, unacceptable toxicities, consent withdrawal or death. The end of the trial is defined as the date of the last visit of the last patient enrolled in the trial. For analysis time points please refer to Section 6.10 on Interim Analysis.

3.2 RATIONALE FOR STUDY DESIGN

This open-label, multicenter, phase Ib design of RO5479599 in combination with pertuzumab and paclitaxel addresses the primary objective of evaluating the safety and tolerability profile of RO5479599 across the dose levels explored. The design is not to be considered a full dose-escalation, as it leverages the evidence collected in the currently on-going phase I on the dose levels chosen for investigation. Similar designs have been proposed (see Morschauser et al. 2010 [53]). Approximately two cohorts of approximately 20 patients were considered sufficient to estimate the incidence of DLTs and other AEs, given that evidence already exists in the monotherapy and combination setting of RO5479599 from the ongoing Phase I Study BP27771.

3.2.1 Rationale for Dosage Selection

The starting dose of RO5479599 in this Phase Ib study will be 1000 mg q3w in 6 patients initially to assess the safety and tolerability followed by 2000 mg dose q3w in additional 20 patients. The RO5479599 doses and schedule (q3w) has been selected taking into consideration the safety, PK and PD data from the phase I dose escalation monotherapy Study BP27771 (see Section 1.2.1.2) and also based on preliminary modeling and simulation to investigate the predicted PK and target saturation in a q3w regimen. The MTD was not reached during the dose escalation of RO5479599 from 100 mg up to 2000 mg. PK and PD analysis were also utilized to guide dose and schedule recommendations for this Phase Ib study and for future studies.

Based upon preliminary PK analysis from the phase I monotherapy dose escalation Study BP27771 (100 mg to 2000 mg q2w; n = 25 patients) (see Section 1.2.1.2.2), the PK of RO5479599 was shown to be non-linear from 100 mg up to 400 mg. Both C_{max} and AUC showed a greater than dose proportional increase, accompanied by a decline in total clearance over the same dose range indicating that the elimination of

RO5479599 is predominantly target-mediated at exposures achieved with these doses (Table 1).Dose proportionality was observed from 800 mg up to 2000 mg, indicating that the proposed doses of 1000 mg and 2000 mg for the Phase Ib study showed linear PK on a q2w regimen based on simulated and observed PK data, respectively. HER3 inhibition was observed from the first dose level onwards with HER3 membranous protein down-regulation in skin and from 200 mg onwards in on-treatment tumor samples.

A 2-compartmental model which includes a linear and non-linear contribution to the elimination of RO5479599 was used to simulate the systemic exposure of RO5479599 over the dosing interval (q3w) during one cycle and 3 subsequent cycles. The projected PK profiles anticipate linear behavior following1000 mg and 2000 mg administration with a q3w dosing interval (Figure 2 and Table 2). The non-linear elimination in the 2-compartmental model was also used to predict the target saturation in humans following IV administration of 1000 mg and 2000 mg of RO5479599 q3w. The proposed doses of 1000 mg and 2000 mg administered q3w were estimated to show linear PK during the q3w interval and to result in approximately >99% (range: >99% to >99%) target saturation during the first cycle of treatment (Table 2).

Figure 2 Simulated Pharmacokinetic Profiles of RO5479599 Following 1000 mg and 2000 mg q3w for Four Consecutive Cycles with 50% Confidence Bounds (25th to 75th Percentile)

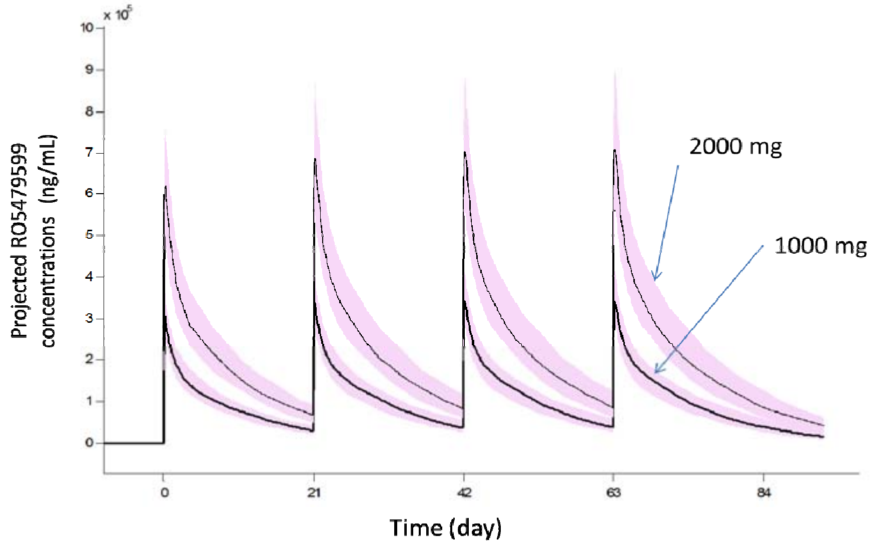

Table 2 Projected Target Saturation in Humans Following RO5479599 IV Doses q3w for Two Consecutive Cycles (Cycle 1 and 2)

Dose (mg)	Cycle No.	HER3 Target Saturation		
		S_{min} (%)	$S_{average}$ (%)	S_{max} (%)
1000	1	96.6	98.7	99.7
	2	97.2	98.7	99.7
2000	1	98.6	99.4	99.9
	2	98.9	99.5	99.9

In Study BP27771, RO5479599 has been well tolerated in the 25 patients receiving RO5479599 as monotherapy with doses from 100 mg to 2000 mg and no DLTs were observed. The dose escalation of RO5479599 in combination with EGFR-inhibitory therapies (i.e. erlotinib or cetuximab) is ongoing. The 25 patients treated so far did not show any unexpected AEs that are not already known and expected with EGFR-inhibitory therapy.

In summary, the current available safety, PK and activity data support 1000 mg RO5479599 as an IV administration q3w as a safe starting dose.

3.2.2 Dose Escalation Decision Criteria for the Dose Escalation and Extension Phases

This is an open-label, multicenter, phase Ib clinical study of RO5479599 in combination with pertuzumab and paclitaxel.

RO5479599 will be administered on a q3w schedule (defined as 1 dose of RO5479599 followed by 20 days without treatment, where 1 cycle is equal to 3 weeks). Pertuzumab will be administered at a loading dose of 840 mg during Cycle 1 followed by 420 mg for the remaining cycles in a q3w schedule. Paclitaxel will be administered at 80 mg/m^2 in a qw schedule.

The study will have a dose escalation phase with 6 patients per dose cohort and additional 6 to 14 patients recruited per cohort for the extension phase. The decision to initiate enrollment at the 2000 mg dose will be based on the safety data from the dose escalation of the 1000 mg cohort corresponding to a 3-week observation period (i.e. 1 cycle of RO5479599) also called DLT period. If 0 or 1 out of the first 6 evaluable patients treated at the 1000 mg dose level experiences a DLT, then the dose of RO5479599 will be escalated to 2000 mg. If 2 out of 6 patients experience a DLT, the decision to initiate enrollment for the 2000 mg cohort will be taken after the sponsor and investigators have reviewed and concluded that the severity and the type of the AEs are medically acceptable. If 3 out of 6 patients experience a DLT then enrollment is stopped.

If 0 or 1 out of the first 6 evaluable patients treated at the 2000 mg dose level experiences a DLT, then further recruitment in this cohort will be initiated to a total of 20 patients in this cohort. If 2 out of 6 patients treated at the 2000 mg dose level experience a DLT, the decision to continue recruitment in this cohort will be taken after the sponsor and investigators have reviewed and concluded that the severity and the type of the AEs are medically acceptable. If 3 out of 6 patients experience a DLT then enrollment is stopped.

Depending on the toxicities observed there will be an option to also expand the 1000 mg cohort to a total of 12 to 20 patients. If the drop-out rate is approximately 10% or above (≥ 2 out of 20 patients not completing week 12 of treatment) then patients may be replaced.

In each dose escalation phase of RO5479599 in combination with pertuzumab and paclitaxel, 6 patients will be enrolled in parallel except for the first two patients where there will be a one-week window between the first two patients.

Should the 1000 mg cohort not fulfill the safety criteria defined above the respective cohort will not be expanded and the higher dose level will not be initiated. Instead lower dose levels of RO5479599 (below 1000 mg of RO5479599) may be evaluated following the same rules as described above, i.e. with a 6 patient dose escalation phase followed by an extension phase as described above. The definition of the lower dose levels will be based on the safety and PK data observed in the trial. An additional extension phase cohort with approximately 20 MBC patients will be included to specifically study the safety and efficacy in patients who did not receive any previous chemotherapy for metastatic disease and/or a maximum of only one prior chemotherapy regimen in adjuvant or neoadjuvant setting.

The decision to extend the cohort and recruit at the higher-dose cohort will be made jointly by the sponsor and the investigator following review of all available safety information collected, including AEs, ECGs, vital signs, clinical laboratory test results, and PK data at the previous dose level(s).

3.2.2.1 Dose-Limiting Toxicity

DLTs are only applicable for the dose escalation phase of the study.

For the purpose of this study, a DLT will be defined as any of the following events attributed to RO5479599 (i.e., related to RO5479599) and occurring during the first cycle (1 cycle = 3 weeks; DLT period = 21 days):

- Hematological toxicities:
    - Grade 4 neutropenia (i.e., absolute neutrophil count [ANC] < 0.5 x 10^9 cells/L) for minimal duration of 7 days.
    - Febrile neutropenia (i.e., ANC < 1.0 x 10^9 cells/L with a single temperature of > 38.3°C or a sustained temperature of ≥ 38°C for more than 1 h).

    - Grade 4 thrombocytopenia (≤ 25.0 x 10^9 cells/L).
    - Grade 3 thrombocytopenia associated with severe bleeding episodes.

- Grade ≥ 3 non-hematological toxicity with the exception of:
    - Alopecia (any grade).
    - Grade 3 nausea and vomiting and diarrhea that respond to optimal management.
    - Grade 3 diarrhea lasting for ≤ 2 days with no fever or dehydration.
    - Laboratory values of ≥ grade 3 which are judged not clinically significant by the investigator.
    - IRRs are not considered DLTs since, based on experience with monoclonal antibodies, IRRs are not dose-related events. Precautions will be taken if IRR grade ≥ 2 occur. If described precautions are not sufficient, other options will be discussed between sponsor and investigator.
- Failure to recover from any treatment-related toxicity grade ≥ 2 which results in a dose delay of > 14 days of the next scheduled administration will be considered a DLT.
- Grade 3 neuropathy that causes a dose delay of > 14 days will be considered a DLT.

There will be frequent and detailed discussions regarding patient eligibility and patient care between the investigators and sponsor. After each patient received RO5479599, the investigator(s) must confirm with the sponsor that the patient was dosed and provide a brief summary of the status of the patient regarding the safety and tolerability of RO5479599 (this will be communicated by email and/or telephone contact). In the event of a DLT, the investigator will contact the sponsor immediately to discuss patient status and action(s) taken/to be taken.

In addition, as outlined above, after each patient cohort has been completed (i.e., last patient in the cohort has reached Day 21), the sponsor will organize a teleconference with the investigators to discuss the safety and tolerability of RO5479599 and to discuss the dose for the next cohort. During these teleconferences, toxicities will be discussed along with the results of the available PK data, in addition to safety laboratory results and any other available data that may assist the dose escalation decision process. Dose escalation will only proceed to the next dose level if the investigators and the sponsor are satisfied with the safety profile of the previous patient cohort and agree to move to the next dose level.

3.2.2.2 Maximum Tolerated Dose

The highest dose level reached in the dose escalation part at which a maximum of 2 out of 6 patients experience a DLT during the dose escalation phase will be considered the MTD. For the purpose of dose escalation, all patients who received one dose of RO5479599 and have been observed for 21 days from 1st dose onwards will be evaluable for DLT assessment, i.e., the treatment interval for determination of the DLT is 21 days (= DLT period). The final evaluation of the toxicity profile of a cohort will be

conducted once recruitment into that cohort is completed, which could occur during the dose escalation or during the extension of the cohort.

3.2.2.3 Recommended Phase Two Dose and Regimen

The decision regarding the RPTD and regimen will take the following into consideration:

- MTD, if reached,
- PK and PD profiles and associated variability
- Preliminary efficacy
- Toxicity information

3.2.3 Rationale for Study Population

The rationale to include HER3-positive tumors is based on non-clinical models demonstrating promising anti-tumor efficacy and lack of anti-tumor efficacy in HER3 negative tumors. Thus, HER3 protein expression is considered as a primary clinical response predictor for RO5479599. Molecular pathology data demonstrated high prevalence of HER3 protein in BC and the crosstalk and compensatory mechanisms of the various HER receptors have been intensively investigated in these tumors.

Non-clinical experiments in BC xenograft mouse models have demonstrated additive anti-tumor efficacy with the combination of RO5479599 and pertuzumab supporting the concept that comprehensive inhibition of HER family heterodimers may confer clinical benefit in these patients. The decision to focus on tumors that do not overexpress HER2 protein at a score of 3+ by IHC or do not harbor HER2 gene amplification by ISH is due to the high unmet medical need in these patients. In contrast, for patients with BC overexpressing HER2 (IHC 3+ and/or ISH+) there are multiple approved targeted treatment options for the front line as well as relapsed situation. It is discussed that tumors with HER2-normal expression tend to form ligand-dependent HER2-HER3 heterodimers, which act as potent and highly regulated signaling transducers. Activated HER2-HER3 signaling promotes tumor cell growth and tumor cell survival via the PI3 kinase/Akt pathway. HER2 IHC 0 tumors will be excluded as no HER2-HER3 heterodimers are expected to be formed.

Dose escalation phase and extension phase 1:

Patients with any number of previous treatment lines for metastatic disease may be enrolled into this study.

Extension phase 2:

In order to assess safety and tolerability as well as preliminary efficacy for a future development of this combination in early breast cancer such as the neoadjuvant setting the combination of paclitaxel, pertuzumab and RO5479599 will be tested in an additional

homogeneous extension phase cohort of patients with no prior chemotherapy for metastatic disease and/or a maximum of only one prior chemotherapy regimen in adjuvant or neoadjuvant setting.

3.2.4 Rationale for Control Group

Not applicable.

3.2.5 Rationale for Biomarker Assessments

Understanding the molecular differences underlying disease subtypes may allow tailoring of drug therapies for improved effectiveness of treatment. Therefore, specimens for analyzing dynamic changes of molecular markers (non-inherited) and genetic biomarkers (inherited) will be collected from all patients participating in the trial.

These specimens will be used for exploratory research purposes to identify biomarkers that are predictive of response to RO5479599 treatment (in terms of dose, safety and tolerability) and will help to better understand the pathogenesis, course and outcome of cancer and related diseases (see Section 4.6.1.10).

As a homogenous patient population will be included in this study and biologically active doses will be administered, the plan is also to do exploratory correlation with efficacy and response prediction biomarkers.

Non-clinical studies have indicated that co-expression or up-regulation of other HER-family members and cMET can impact the response to RO5479599 treatment. Furthermore, these non-clinical studies demonstrated that certain mutations (e.g. kRas or HER4) more often occurred in models which did not respond to HER3-targeted treatment.

3.3 OUTCOME MEASURES

3.3.1 Safety Outcome Measures

The safety outcome measures for this study are as follows:

- Incidence of DLTs
- Incidence and severity of AEs
- Incidence of laboratory abnormalities, based on hematology and clinical chemistry test results
- Incidence of anti-RO5479599 antibodies (human anti-human antibodies [HAHAs])
- Findings on physical examination
- Careful observation of patients for IRRs
- ECGs
- Echocardiography
- Vital signs

The NCI CTC-AE version 4.03 (NCI NIH CTCAE v.4 data files) will be used to evaluate the clinical safety of the treatment in this study. Patients will be assessed for AEs at each clinical visit and as necessary throughout the study.

Please refer to Schedule of Assessments (Appendix 1) for details on collection time of the assessments outlined below.

3.3.2 Pharmacokinetic and Pharmacodynamic Outcome Measures

3.3.2.1 Pharmacokinetic Outcome Measures

The PK outcome measures for this study include the PK profile and parameters derived from the serum concentration-time profile following administration of RO5479599, including those parameters listed below:

- Maximum serum concentration (C_{max})
- Trough serum concentration (C_t) at the beginning of Cycle 2 and at the beginning of every subsequent cycle where PK data are available.
- Time to reach maximum serum concentration (t_{max})
- Area under the concentration-time curve (AUC)
- Clearance (CL)
- Volume of distribution (V)
- Accumulation ratio
- Elimination half-life ($t_{1/2}$)
- Serum concentration at the time of tumor progression (C_{prog})
- Serum concentration at the time of tumor response (CR/PR)
- Serum concentration at the time of DLT
- Serum concentration at the time of tumor and skin biopsy (C_b)
- Serum concentration at time of IRR

PK profiles and parameters derived from the serum concentration-time profile following administration of paclitaxel and pertuzumab will also be determined in Extension Phase Cohort 2.

3.3.2.2 Pharmacodynamic Outcome Measures

The PD outcome measures for this study are based on blood and serum markers and are as follows:

- Leukocyte immunophenotyping to examine peripheral immune modulation and related parameters
- NK lymphocyte functional assessment (NK cell activation measured by CD107a)

3.3.3 Efficacy Outcome Measures

The efficacy/activity outcome measures for this study are as follows:

- ORR
- DCR
- Duration of response
- PFS
- OS

Please see Section 6.6 for a definition of these terms.

3.3.4 Patient-Reported Outcome Measures

Not applicable.

3.3.5 Exploratory Outcome Measures

The exploratory outcome measures for this study include but are not limited to the relationship between PK and PD data, response prediction biomarkers and efficacy variables.

4. MATERIALS AND METHODS

4.1 CENTER

This is a multi-center study to be conducted in Europe. Additional countries and sites may be included for back-up purposes and may be activated if needed.

4.2 STUDY POPULATION

Patients with MBC expressing HER3 and HER2 protein will be included into the study.

4.2.1 Recruitment and Replacement Procedures

Patients will be identified for potential recruitment using pre-screening enrollment logs, IEC/IRB approved newspaper/radio advertisements and mailing lists, (if applicable) prior to consenting to take part in this study.

If a patient withdraws consent prior to treatment administration, the patient will be replaced. During the dose escalation phase, patients who withdraw before the end of the DLT period (i.e., within 21 days of the first dose) for reasons other than DLTs will be replaced to ensure that all patients in each cohort have been assessed for a full DLT period prior to moving to the next dose level.

In Extension Phase Cohort 2, 6 patients have to be evaluable for safety over a period of 2 cycles (i.e. 6 weeks).

If the drop-out rate is approximately 10% or above (≥ 2 out of 20 patients not completing Week 12 of treatment) then patients may be replaced.

Additionally patients in the extension phase who discontinue treatment before evaluation of response will be replaced.

A center may be replaced for the following administrative reasons:

- Excessively slow recruitment.
- Poor protocol adherence.

4.2.2 Procedures for Screening Patients for HER3 Expression in Tumor Tissue and Assessment of HER2 status

RO5479599 has been shown to bind selectively and with high affinity (dissociation constant in the range of 1 nM) to the extracellular domain (ECD) of human and cynomolgus monkey HER3.

The expression of HER3 in non-clinical tumor models strongly correlated to response to RO5479599. Non-clinical tumor models lacking HER3 expression consistently did not demonstrate any response to RO5479599.

Thus, it is anticipated that patients will only derive therapeutic benefit from the therapy if their tumor expresses HER3 protein.

The HER2 status and the expression status of HER3 will be determined centrally during screening. To this end it is mandated that a fresh pre-treatment tumor biopsy, fixed in 10% neutral buffered formalin and embedded into paraffin, will be obtained. To ensure high quality testing, the tumor biopsy must consist of a representative area of the tumor containing at least 100 neoplastic cells. Upon receipt, the central lab will subject the tumor biopsy sample to a quality control assessment by histopathological evaluation (e.g., tumor cell content and tissue integrity). If quality criteria are met the tumor biopsy sample will be assessed for HER2 and stained for HER3 protein expression. For HER2, parallel testing of protein expression and gene amplification is required, using an FDA-approved test. HER2 status will be defined as described in the respective assay working procedure. HER3 testing will be based on protein expression only using a robust prototype assay which will be provided by Ventana Medical Systems (VMS).

If sample quality criteria cannot be met for the provided tumor biopsy sample a replacement of the tumor biopsy might be requested if possible. For detailed procedures on biopsy taking, handling and shipping as well as diagnostic procedure and scoring please refer to the laboratory manual.

4.2.3 Inclusion Criteria

Dose escalation phase and extension phase 1:

Patients who meet all of the following criteria will be included into the study:

1. Signed informed consent, ability and willingness to comply with all aspects of the protocol, as required by the investigator.
2. Age ≥ 18 years.
3. ECOG performance status (PS) 0 to 1.
4. Histologically confirmed MBC patients eligible for enrollment must be willing to undergo a fresh (pretreatment) tumor/metastases biopsy that will be used to assess the level of HER3 protein expression by IHC and central pathology review. A formalin fixed paraffin embedded tumor (FFPET) block of the core needle biopsy[1] must be submitted for central IHC staining and review. Fine needle aspiration (FNA), biopsy of bone lesions, or archived primary tumor tissue is not acceptable to determine eligibility. Core needle biopsy specimens should contain at least 100 viable neoplastic cells. Discernible HER3 membrane positivity in any neoplastic cell will be considered diagnostically positive for HER3 protein expression and eligible for enrollment.
5. HER2 status confirmed on same tumor/metastases by a central laboratory: BC tumors and/or metastases must be HER2 IHC 1+/ISH- or HER2 IHC 2+/ISH- as assessed by parallel testing of protein and gene amplification using an FDA-approved test.
6. Taxane-naïve patients or patients who have received taxanes as part of an adjuvant/neoadjuvant treatment regimen with a disease-free interval of at least 1 year. **Patients who have received a docetaxel-containing regimen in the metastatic setting may be eligible. Patients who have received paclitaxel/nab-paclitaxel in the metastatic setting but have discontinued paclitaxel/nab-paclitaxel for a reason other than disease progression and have had a taxane-free interval of at least 6 months may be eligible unless otherwise contraindicated at the investigator's discretion.**
7. Radiologically measurable or clinically evaluable disease according to RECIST criteria.
8. Last dose of systemic anti-neoplastic therapy > 21 days prior to first study treatment infusion. Palliative radiotherapy is allowed up to 2 weeks before the first study treatment infusion.
9. All acute toxic effects of any prior radiotherapy, chemotherapy or surgical procedure must have resolved to Grade ≤ 1, except alopecia (any grade).

---

[1] For superficial lesions a punch biopsy can be performed instead if an adequately deep specimen can be warranted and including a representative amount of tumor cells. Therefore, the punch biopsy should have a diameter of ≥ 2 mm at least.

10. Adequate hematological function: neutrophil count of ≥ 1.5 x 10^9 cells/L, platelet count of ≥ 100,000/μL, hemoglobin (Hb) ≥ 10 g/dL (6.2 mmol/L).
11. Adequate liver function: bilirubin ≤ 1.5 x ULN (excluding Gilbert's Syndrome, see below), aspartate aminotransferase (AST) and/or alanine aminotransferase (ALT) ≤ 2.5 x ULN (in case of liver metastases or patients with hepatocellular carcinoma: ≤ 5 x ULN).
12. Adequate renal function: serum creatinine ≤ 1.5 ULN or creatinine clearance (by Cockcroft Gault formula [see Appendix 4]) ≥ 50 mL/min for patients in whom, in the investigator's judgment, serum creatinine levels do not adequately reflect renal function.
13. Baseline left ventricular ejection fraction (LVEF) of ≥ 50% (measured by echocardiography).
14. Negative serum pregnancy test within 7 days prior to start of study treatment in premenopausal women and women ≤ 2 years after menopause (menopause is defined as amenorrhea for > 2 years).
15. Female patients must be postmenopausal, surgically sterile, or they must agree to use a highly effective form of contraception by the patient and/or partner. Contraception use must continue for the duration of the study and for at least 6 months after the last dose of study medication.
16. Male patients must agree to use a barrier method (condom) during the study and for at least 6 months after last dose of study medication.

Gilbert's Syndrome

Patients with Gilbert's Syndrome will be eligible for the study. The diagnosis of Gilbert's Syndrome is suspected in people who have persistent, slightly elevated levels of unconjugated bilirubin without any other apparent cause. A diagnosis of Gilbert's syndrome will be based on the exclusion of other diseases based on the following criteria:

- Unconjugated hyperbilirubinemia noted on several occasions
- No evidence of hemolysis (normal hemoglobin, reticulocyte count and lactate dehydrogenase [LDH])
- Normal liver function tests
- Absence of other diseases associated with unconjugated hyperbilirubinemia

Extension phase 2:

All of the above except inclusion criterion #6. In addition:

17. Patients with no prior chemotherapy for MBC and/or a maximum of only one prior chemotherapy regimen in adjuvant or neoadjuvant setting.
18. Taxane-naïve patients or patients who have received taxanes as part of an adjuvant/neoadjuvant treatment regimen with a disease-free interval of at least 1 year.

4.2.4 Exclusion Criteria (All Patients)

Patients who meet any of the following criteria will be excluded from study entry:

1. Concurrent therapy with any other investigational drug (defined as treatment for which there is currently no regulatory authority approved indication).
2. History or clinical evidence of central nervous system (CNS) primary tumors or metastases including leptomeningeal metastases unless they have been previously treated, are asymptomatic and have had no requirement for steroids or enzyme-inducing anti-convulsants in the last 14 days.
3. Evidence of significant, uncontrolled concomitant diseases which could affect compliance with the protocol or interpretation of results, including uncontrolled diabetes mellitus.
4. Active or uncontrolled infections.
5. Known human immunodeficiency virus (HIV) or known active hepatitis B virus (HBV) or hepatitis C virus (HCV) infection.
6. Any other diseases, metabolic dysfunction, physical examination finding or clinical laboratory finding giving reasonable suspicion of a disease or condition that would contraindicate the use of an investigational drug.

7. Major surgery or significant traumatic injury < 28 days prior to the 1st study treatment infusion (excluding biopsies) or anticipation of the need for major surgery during study treatment.
8. Dementia or altered mental status that would prohibit informed consent.
9. Pregnant or breast-feeding women.
10. Known hypersensitivity to any of the components of RO5479599, pertuzumab or paclitaxel.
11. Patients with contraindications for paclitaxel therapy according to the SmPC (see http://www.actavis.ie/NR/rdonlyres/6FEC3CFF-8F21-4FEE-B7BB-4198577A99E0/18005/3SPC_PA1380591_230712_C2106797.pdf).
12. Therapy with an antibody or immunotherapy (i.e., interferon-α, interferon-β, interleukin-2, etanercept, infliximab, tacrolimus, cyclosporine, mycophenolic acid, alefacept or efalizumab) concurrently or within a period of time where drug exposure is still considered biologically active (usually < 5x $t_{1/2}$) prior to first dose of study treatment.
13. Regular immunosuppressive therapy (i.e., for organ transplantation, chronic rheumatologic disease).
14. Concurrent high doses of systemic corticosteroids. High dose is considered as > 20 mg of dexamethasone a day (or equivalent) for > 7 consecutive days.
15. Baseline QTc interval of > 470 ms, patients with baseline resting bradycardia < 45 beats per minute or baseline resting tachycardia > 100 beats per minute.
16. Uncontrolled hypertension (systolic > 150 mmHg and/or diastolic > 100 mmHg), unstable angina, congestive heart failure (CHF) of any New York Heart Association (NYHA) classification, serious cardiac arrhythmia requiring treatment (exceptions: atrial fibrillation, paroxysmal supraventricular tachycardia), history of myocardial infarction within 6 months of enrollment or symptomatic LVEF dysfunction.
17. A history of grade ≥ 3 peripheral neuropathy of any etiology.

4.3 METHOD OF TREATMENT ASSIGNMENT

Patients cannot commence enrollment procedures until all entry criteria have been fulfilled. Where the clinical significance of an abnormal screening test result (lab or any other tests) is considered uncertain, the test may be repeated.

Under no circumstances will patients who enroll in this study and have completed treatment as specified, be permitted to be allocated a new enrollment number and re-enroll in the study. However, patients who started screening and could not complete the entire screening process and receive study treatment for whatever reason will have the opportunity to restart the screening and enroll in the study. The biopsies taken during initial screening will still be used for these patients. All other screening assessments will need to be repeated.

The investigator or designee will use the eCRF with the assigned patient number and enter the corresponding number for allocation to the treatment groups in the appropriate place on each patient's eCRF. Once a patient has successfully fulfilled the entry criteria, they will be sequentially assigned to a set of patient numbers. The assigned dosing cohort and dose level will be communicated to the investigator at the time of individual patient enrollment. Once a cohort is filled, the investigator will be informed. Patients will be assigned in a sequential manner to the dosing group open at the time of the patient's enrollment.

A patient Enrollment and Identification Code List must be maintained by the investigator.

Upon completion of all screening evaluation and verification that patients have met all inclusion criteria, patients will be enrolled at investigator sites based upon competitive recruitment once the dose escalation phase has been completed in the respective dose cohort.

4.4 STUDY TREATMENT

For the purpose of the study, RO5479599, pertuzumab and paclitaxel are considered study treatments.

4.4.1 Formulation, Packaging and Handling

Investigational Medicinal Product (IMP)
200 mg/20 mL RO5479599
420 mg/14 mL Pertuzumab
150 mg Paclitaxel

4.4.1.1 RO5479599

RO5479599 packaging will be overseen by the Roche clinical trial supplies department and bearing a label with the identification required by local law, the protocol number, drug identification and dosage.

The packaging and labeling of RO5479599 will be in accordance with Roche standard and local regulations.

RO5479599 must be stored according to the details on the product label.

Upon arrival of investigational products at the site, site personnel should check them for damage and verify proper identity, quantity and temperature conditions, and report any deviations or product complaints to the monitor upon discovery.

For further details, see the RO5479599 Investigator's Brochure.

4.4.1.2 Pertuzumab

Pertuzumab is provided as a single-use formulation containing 30 mg/mL pertuzumab formulated in 20 mM L-histidine-acetate (pH 6.0), 120 mM sucrose and 0.02% polysorbate 20. Each 20-cm^3 vial (14.0 mL solution per vial) contains approximately 420 mg of pertuzumab.

Pertuzumab packaging will be overseen by the Roche clinical trial supplies department and bearing a label with the identification required by local law, the protocol number, drug identification and dosage.

The packaging and labeling of pertuzumab will be in accordance with Roche standard and local regulations.

Pertuzumab must be stored according to the details on the product label.

Upon arrival of investigational products at the site, site personnel should check them for damage and verify proper identity, quantity and temperature conditions, and report any deviations or product complaints to the monitor upon discovery.

For further details, see the Pertuzumab SmPC.

4.4.1.3 Paclitaxel

Paclitaxel is provided a single use 150 mg formulation.

Paclitaxel packaging will be overseen by the Roche clinical trial supplies department and bearing a label with the identification required by local law, the protocol number, drug identification and dosage.

The packaging and labeling of Paclitaxel will be in accordance with Roche standard and local regulations.

Paclitaxel must be stored according to the details on the product label.

Upon arrival of investigational products at the site, site personnel should check them for damage and verify proper identity, quantity and temperature conditions, and report any deviations or product complaints to the monitor upon discovery.

4.4.2 Dosage and Administration

Study treatments will be administered to the patients by investigational staff in the consecutive sequence of paclitaxel, pertuzumab and RO5479599 on Day 1 of each 3-weekly treatment cycle except for Cycle 1. For Cycle 1, it is recommended to administer paclitaxel and pertuzumab on Day 1 and RO5479599 on Day 2. If administration of the selected doses is not possible on the planned day due to IRRs it is allowed to resume treatment on the following day.

RO5479599 and pertuzumab will be given as an absolute (flat) dose and paclitaxel will be given at standard dose by Body Surface Area (BSA).

4.4.2.1 Paclitaxel

Paclitaxel should only be administered under the supervision of a physician experienced in the use of cancer cytotoxic agents.

4.4.2.1.1 Pre-medication for Patients Receiving Paclitaxel

All patients must be premedicated prior to paclitaxel administration to prevent severe hypersensitivity reactions according to the SmPC (please see http://www.actavis.ie/NR/rdonlyres/6FEC3CFF-8F21-4FEE-B7BB-4198577A99E0/18005/3SPC_PA1380591_230712_C2106797.pdf). Such premedication may consist of dexamethasone, promethazine (or its equivalent) and cimetidine or ranitidine according to Institutional Guidelines. Paclitaxel should be administered through an in-line filter with a microporous membrane not greater than 0.22 µm.

Significant hypersensitivity reactions can occur in patients receiving taxanes, even after receiving adequate pre-medication. In the case of severe hypersensitivity reactions, paclitaxel infusion should be discontinued immediately, symptomatic therapy should be initiated and the patient should not be rechallenged with the taxane. In particular, macrogolglycerol ricinoleate, an excipient in paclitaxel, can cause hypersensitivity reactions.

4.4.2.1.2 Infusion of Paclitaxel

Paclitaxel should be administered as an IV infusion weekly, i.e. on Day 1, 8 and 15 of each 3-weekly treatment cycle (q3w), at a dose of 80 mg/m^2 according to the recognized clinical practice guidelines and/or institutional guidelines. Patients will be observed for a further 60 min from the end of infusion for hypersensitivity-related symptoms such as fever, chills etc.

4.4.2.2 Pertuzumab

4.4.2.2.1 Pre-medication for Patients Receiving Pertuzumab

The investigator must ensure that the intended pre-medication is suitable for patients according to the Pertuzumab SmPC. Pertuzumab must be administered in a setting with emergency equipment and staff trained to monitor and respond to emergency situations. Patients who experience infusion-associated symptoms may be pre-medicated (e.g., antihistamines, paracetamol, corticosteroids) for subsequent infusions.

4.4.2.2.2 Infusion of Pertuzumab

The infusion of pertuzumab will commence 60 min after the end of the paclitaxel infusion according to the recognized clinical practice guidelines and/or institutional guidelines. Pertuzumab will be administered as an IV infusion on Day 1 of 3-weekly cycles (q3w). On Day 1 of Cycle 1, patients will receive a loading dose of 840 mg of pertuzumab, followed by 420 mg on Day 1 of each subsequent cycle. **Patients in Extension Cohort**

2 will commence at a reduced dose of pertuzumab, i.e. 420 mg for Cycle 1 followed by 420 mg for subsequent cycles.

Initial infusions of pertuzumab will be administered over 60 (±10) min and patients observed for a further 60 min from the end of infusion for IRR symptoms such as fever, chills etc. (see Section 4.4.2.6.1 for treatment of IRRs). Interruption or slowing of the infusion may reduce IRR symptoms. If the infusion is well tolerated, subsequent infusions may be administered over 30 to 60 (±10) min, with patients observed for a further 30 min. The subsequent infusion of RO5479599 only proceeds 60 min after the end of the pertuzumab infusion.

Pertuzumab administration may be delayed to assess or treat AEs such as cardiac AEs, myelosuppression or other events (see Section 4.4.2.5).

In the case of surgery during the study, there is no evidence that the HER2 antibodies delay wound healing, but patients should have recovered from surgery and anesthesia (including liver functions) for a minimum of 14 days before antibody treatment.

4.4.2.3 RO5479599

The selected starting dose of RO5479599 is 1000 mg given as an IV infusion on Day 1 of 3-weekly cycles (q3w). However, for Cycle 1, it is recommended to administer RO5479599 on Day 2 (see also Section 4.4.2).

RO5479599 must be administered in a hospital or clinic equipped for IV chemotherapy. Full emergency resuscitation facilities should be immediately available and patients should be under close supervision of the investigator at all times.

RO5479599 must be diluted with NaCl (0.9%) to a final infusion volume of 250 mL and should be given as IV infusion through a dedicated line with an in-line filter and recommended product contact surfaces. IV infusion pumps should be used to control the infusion rate of RO5479599. Do not administer as an IV push or bolus.

Please note: Detailed information about the preparation of the infusion solution and administration of RO5479599 including information about compatible infusion bags, administration sets and in line filters can be found in a separate study manual.

During infusion, vital signs (including, if possible, supine diastolic and systolic blood pressure, pulse rate and temperature) have to be monitored pre-infusion, every 15 min until the end of infusion, and thereafter, every 30 min until the infusion line is removed. After completion of Cycle 5 vital signs have to be monitored only pre and post infusion if RO5479599 infusion has been tolerated well in previous cycles. Vital signs during infusion are not required to be captured in the eCRF unless abnormalities are observed.

After the end of the first infusion, the IV line should remain in place for 2 h. If no infusion-related symptoms occur during this time, the infusion line may be removed. For subsequent infusions and if no IRR has been reported, the IV line should remain in place for 30 min from the end of infusion. If no AEs occur during the 30 min, the infusion line may be removed. If feasible, the line for drawing blood for PK samples (opposite extremity to the one with the infusion line) will remain in place until the 24-h sample is taken if required.

The qualified individual responsible for dispensing the study treatments will prepare the correct dose according to the randomization schedule. This individual will write the date dispensed and patient number and initials on the study treatment vial label and on the Drug Accountability Record. This individual will also record the study treatment batch or lot number received by each patient during the study.

Guidelines for dosage modification and treatment interruption or discontinuation are provided in Section 5.1.

4.4.2.3.1 Pre-medication for Patients Receiving RO5479599

The investigator must ensure that the intended pre-medication is suitable for patients according to national prescribing information.

30 min prior to start of the 1st infusion paracetamol (500 to 1000 mg orally) and diphenhydramine (25 to 50 mg orally or IV; or an alternative anti-histamine at an adequate dose) will be administered. In case pre-medication is given for pertuzumab, as pertuzumab is given prior to RO5479599, the investigator may decide whether repetition of pre-medication is necessary based on the time interval between pertuzumab and RO5479599 administration and the half-life of the pre-medication used at the respective institution.

If one patient in a single dose cohort develops an IRR of ≥ grade 3 during the 1st infusion, pre-medication including corticosteroids for subsequent patients may be explored in the course of the study and based on IRR episodes observation.

For subsequent infusions: If IRRs occur in a patient during the 1st infusion, the infusion time may be prolonged and pre-medication may be given for subsequent infusions in this patient.

Table 3 Pre-medication for RO5479599

Pre-medication	Dose (mg)	Route
Paracetamol	500 to 1000	Orally
Diphenhydramine [a]	25 to 50	orally or IV

[a] or alternative anti-histamine at an adequate dose.

4.4.2.3.2 Infusion of RO5479599

The first infusion of RO5479599 will be started at an infusion rate of 50 mL/h. If well tolerated (i.e. in absence of ≥ grade 2 IRRs) the infusion rate may be increased to 100 mL/h after 30 min. Subsequently increases of infusion rate to 150 mL/h and 200 mL/h may be applied every 30 min. If the first infusion was well tolerated, subsequent infusions can be administered over 90 min. In the event of IRRs ≥ grade 2 during the 1st infusion, the same administration schedule as for the first infusion will apply for the 2nd infusion in the patient. If one ≥ grade 3 IRR occurs during the 1st infusion corticosteroids (e.g. 200 mg hydrocortisone or equivalent doses of prednisolone or dexamethasone) may be used as pre-medication if deemed necessary by investigator and sponsor. If one ≥ grade 3 IRR occurs during the 1st infusion despite intensified pre-medication the infusion rate will be decreased for all subsequent patients by going from a ml/h infusion rate to a mg/h infusion rate. In addition, higher corticosteroid doses (400 mg hydrocortisone or equivalent doses of prednisolone or dexamethasone) may be used as pre-medication if deemed necessary by investigator and sponsor.

In case of a ≥ grade 2 IRR occurring for the first time in a patient at the 2nd or subsequent cycles, the infusion rate will be decreased by going from a mL/h infusion rate to a mg/h infusion rate for the subsequent infusion in this patient. In case the infusion is stopped and re-started on the same day then the need for additional pre-medication will be decided on by the investigator. If the infusion is restarted > 1 day after the IRR episode the patient will receive full pre-medication before re-start of the infusion, including corticosteroids, if judged medically indicated by the investigator and the sponsor. The use of mL/h infusion rate or mg/h infusion rate and pre-medication for all following infusions in this patient will be discussed between the investigator and the sponsor. Detailed guidance (e.g. infusion mg/h rate increment guidance) will be provided to investigators.

If all above specified precautions failed, further risk minimization options will be discussed between sponsor and investigator and put in place, e.g. capping of first dose and escalation of 2nd dose or splitting of 1st dose.

In summary:

- First infusion: will be administered over approximately 2 h.

- Second and subsequent infusions: If the first infusion is well tolerated, defined by an absence of ≥ grade 2 IRRs, the second infusion and subsequent infusions may be administered over 90 min.

Dose, date and time of the infusion commencement, date and time of end of infusion (EOI), interruption or adjustment in infusion rate, and reason will be recorded for each patient. If infusion is interrupted, the date and time of stopping and resuming infusion will be recorded.

4.4.2.4 Schedule of Administration of Paclitaxel, Pertuzumab and RO5479599

Study treatments will be administered in the consecutive sequence of paclitaxel, pertuzumab and RO5479599 on Day 1 of each treatment cycle except for Cycle 1. For Cycle 1, it is recommended to administer paclitaxel and pertuzumab on Day 1 and RO5479599 on Day 2. There will be a 1 h delay between treatments (i.e., between paclitaxel and pertuzumab infusions and between pertuzumab and RO5479599 infusions).

In the event of an IRR during or after study treatment infusion, the infusion of the consecutive study treatment (i.e., pertuzumab or RO5479599) will be proceeded only when all symptoms have completely resolved in the investigator’s opinion. In case of prolonged time between start of one and the other study treatment administration (i.e., ≥ 6 h) the pre-medication will be repeated as to the investigator’s discretion to fulfill the protocol recommendation.

The q3w administration is scheduled as follows:

Cycle 1:

Day1:

- Pre-medication for paclitaxel as described in Section 4.4.2.1.1.
- Paclitaxel 80 mg/m^2 infusion as described in Section4.4.2.1.2.
- Pre-medication for pertuzumab as described in Section 4.4.2.2.1.
- Pertuzumab 840 mg IV infusion**. For Extension Phase Cohort 2, pertuzumab 420 mg IV infusion** as described in Section 4.4.2.2.2.

Day 2:

- Pre-medication for RO5479599 as described in Section 4.4.2.3.1.
- RO5479599 as an IV infusion as described in Section 4.4.2.3.2.

Day 8:

- Pre-medication for paclitaxel as described in Section 4.4.2.1.1.
- Paclitaxel 80 mg/m^2 IV infusion as described in 4.4.2.1.2.

Day 15:

- Pre-medication for paclitaxel as described in Section 4.4.2.1.1.
- Paclitaxel 80 mg/m^2 IV infusion as described in Section 4.4.2.1.2.

From Cycle 2 onwards:

Day 1:

- Pre-medication for paclitaxel, pertuzumab and RO5479599 as described in Section 4.4.2. Pre-medications must be administered with the respective time interval prior to the infusions. The investigator may decide whether repetition of pre-medication with antihistamines is necessary based on the time interval between pertuzumab and RO5479599 administration and the half-life of the pre-medication used at the respective institution.
- Paclitaxel 80 mg/m^2 infusion as described in Section 4.4.2.1.2.
- Pertuzumab 420 mg IV infusion as described in Section 4.4.2.2.2.
- RO5479599 at the determined dose as an IV infusion as described in Section 4.4.2.3.2.

Day 8:

- Pre-medication for paclitaxel as described in Section 4.4.2.1.1.
- Paclitaxel 80 mg/m^2 IV infusion as described in Section 4.4.2.1.2.

Day 15:

- Pre-medication for paclitaxel as described in Section 4.4.2.1.1.
- Paclitaxel 80 mg/m^2 IV infusion as described in Section 4.4.2.1.2.

4.4.2.5 Toxicity Assessments, Dose Delays and Dose Modifications for Toxicity

If one drug needs to be permanently discontinued due to related toxicities such as IRRs the patient may remain on the study and continue with the remaining study drugs if the patient has completed the DLT period (dose escalation phase only) and experienced clinical benefit from the study treatments, i.e. stable disease or partial/complete response.

In case of paclitaxel-related toxicities the dose of paclitaxel may be reduced once to 60 mg/m^2 at the investigator’s discretion. If paclitaxel needs to be permanently discontinued due to paclitaxel-related toxicities such as neuropathy or hematological toxicities the patient may remain on the study and continue with the remaining study treatments if the patient has completed the DLT period (dose escalation phase only) and experienced clinical benefit from the study treatments, i.e. stable disease or partial/complete response.

No dose reductions are foreseen for pertuzumab or RO5479599. Paclitaxel, pertuzumab and RO5479599 administration may be delayed to assess or treat related and unrelated AEs. For related AEs, a dose delay of 21 days (1 cycle) is acceptable otherwise the patient will be discontinued from the study (for exceptions see Table 4 **and Table 5**). For unrelated AEs, the dose delay may go beyond 21 days if the patient benefits from the treatment.

Patients should not be retreated with paclitaxel until recovery has occurred with platelets ≥ 100 x 10^9/L and ANC ≥ 1.5 x 10^9/L. All dose adjustments should be based on the worst preceding toxicity. Particular toxicities are described in Table 4 **and Table 5**.

Table 4 Dose Delays and Dose Modifications of Paclitaxel

**Toxicity**	**Action**
Neutropenia	Neutrophils have to recover to 1500/mm^3 before the start of the next chemotherapy cycle. After one episode of neutrophils < 1500/mm^3: Delay paclitaxel until recovery of neutrophils ≥ 1500/mm^3. Then restart at 80 mg/m^2.<br>2nd/3rd occurrence: Delay paclitaxel until recovery of neutrophils ≥ 1500/mm^3. Then restart paclitaxel at 60 mg/m^2.<br>Any further reoccurrence: Delay paclitaxel until recovery of neutrophils ≥ 1500/mm^3.<br>Growth factors, such as G-CSF should be considered.<br>Permanently discontinue paclitaxel if AE persist > 21 days.
Thrombocytopenia	Platelets have to recover to ≥ 100 x 10^9/L before the start of the next chemotherapy cycle. If this results in a delay of the next treatment application, a full blood count has to be repeated every second day to restart treatment as soon as possible. If platelets have not recovered after a delay of treatment of 21 days, treatment should be discontinued.<br>After one episode of thrombocytopenia grade 4 (< 25000/µL) or a thrombocytopenia grade 3 with severe bleeding episode or a second episode of prolonged recovery (defined as delay of treatment), paclitaxel dose should be reduced to 60 mg/m^2 thereafter, or if the dose was already reduced, paclitaxel should be permanently stopped.
Neuropathy grade 3	Delay treatment until resolution to grade ≤ 1. Restart paclitaxel at 60 mg/m^2 for all subsequent cycles. No further dose reduction of paclitaxel is foreseen.<br>Permanently discontinue paclitaxel if there is no resolution to ≤ grade 1 within 21 days.
Neuropathy grade 4	Permanently discontinue drug. Patient will be discontinued from the study.

Toxicity	Action
Rash ≥ grade 3	Discontinue treatment until resolution to grade ≤ 1 and treat symptomatically.

Table 5 Dose Delays and Dose Modifications of Pertuzumab, RO5479599 and Paclitaxel in Case of Diarrhea

Toxicity	Action
Quality of life-impairing diarrhea grade 2	In case of persistence of diarrhea grade 2 with impaired quality of life for > 14 days consider delaying treatment.<br>If diarrhea resolves to grade ≤ 1 restart treatment either with paclitaxel at 60 mg/m^2/pertuzumab/RO5479599 or discontinue pertuzumab permanently (based on clinical judgment).<br>In case of re-occurrence of persistent grade 2 diarrhea with impaired quality of life discontinue pertuzumab (if not already done at 1st occurrence) or pertuzumab and RO5479599 permanently.
Diarrhea grade ≥ 3	• Delay dosing of paclitaxel/pertuzumab/RO5479599 immediately.<br>• Re-evaluate diarrhea every 2nd day.<br>• If diarrhea grade ≥ 2 with impaired quality of life persists for > 21 days, stop study treatment permanently. In patients demonstrating clinical benefit (i.e. SD, PR or CR as per RECIST criteria or per clinical judgment) after discussion between sponsor and investigator treatment may be resumed after more than 21 days delay.<br>If diarrhea resolves to grade ≤ 1 restart treatment either with paclitaxel at 60 mg/m^2/pertuzumab/RO5479599 or discontinue pertuzumab (based on clinical judgment).<br>Re-occurrence of grade 3 diarrhea discontinue pertuzumab (if not already done at 1st occurrence) or pertuzumab and RO5479599 permanently.

Incomplete Loading Dose **(applicable for the 840-mg loading dose only)**

In case the whole loading dose of pertuzumab cannot be administered due to an IRR or other reason, the following guidelines apply:

The patient should receive at least 50% of the loading dose in the first week. Therefore, if the patient receives less than 50% of the Cycle 1 dose, the patient should receive the remainder before Day 22, preferably within the first week. Thereafter, the patient should receive the usual maintenance dose 3 weeks after the first interrupted dose, as routinely scheduled. For example, if a patient received only approximately 50% of the scheduled

loading dose (i.e., only 420 mg instead of 840 mg of pertuzumab) then the patient should receive the remaining dose (i.e., 420 mg of pertuzumab) preferably in the first week, and then regular maintenance doses (420 mg of pertuzumab) on Day 22, as routinely scheduled. If the patient receives between 50 to 75% of the dose, the patient should receive the remainder before Day 22, preferably within the first two weeks of Cycle 1. For example, if a patient received only approximately 60% of the scheduled loading dose, the patient should receive the remaining 40% within 2 weeks after the interrupted loading dose. Thereafter, the patient should receive the regular maintenance doses on Day 22, as routinely scheduled.

If the patient received ≥ 75% of the loading dose, additional loading is probably not necessary. However, the remainder of the loading dose may be given at the investigator's discretion. In such a case, the remainder may be given at any time before the next scheduled dose or the patient may be given an additional loading dose on Day 22. If, after receiving an incomplete loading dose on Day 1, the patient cannot attend the site until Day 22, the patient should receive a second loading dose on Day 22. However, every effort should be made to give the remainder of the dose prior to Day 22.

4.4.2.6 Management of Toxicities

The NCI-CTCAE version 4.0 will be used to grade toxicity.

Paclitaxel, pertuzumab and RO5479599 will be given as specified in Section 4.4.2

Before starting a new treatment cycle, toxicity must have resolved as specified in the following sections.

4.4.2.6.1 Paclitaxel, Pertuzumab and RO5479599 Toxicities

Toxicities for pertuzumab, paclitaxel and RO5479599 should be managed as per the local prescribing information and/or per institutional practice (see the SmPC of paclitaxel under http://www.actavis.ie/NR/rdonlyres/6FEC3CFF-8F21-4FEE-B7BB-4198577A99E0/18005/3SPC_PA1380591_230712_C2106797.pdf, the SmPC of Pertuzumab and the Investigator's Brochure of RO5479599).

Diarrhea

- **First and early manifestation of diarrhea should be promptly treated with anti-diarrheal treatment (i.e. loperamide), following local institutional guidelines. Patients should be informed of the start and type of treatment and diet to follow. Details of the anti-diarrheal treatment given to patients must be documented in the eCRF.**
- **Delay the combination treatment by up to 1 cycle (i.e. 3 weeks) in patients who experience quality of life-impairing diarrhea of Grade 2 or diarrhea of Grade ≥3 and eventually discontinue pertuzumab or pertuzumab and RO5479599 as described in Section 4.4.2.5.**

- **Weekly monitoring of blood electrolyte levels in patients with Grade ≥2 diarrhea and/or in patients with any electrolyte imbalance. Blood electrolyte analysis should include at least sodium, chloride, calcium, phosphate, potassium, and magnesium.**
- **Implement electrolyte repletion at early signs of electrolyte imbalance as per local institutional guidelines.**

Infusion-Related Reaction

If an IRR develops, the infusion of pertuzumab or RO5479599 should be temporarily slowed down or interrupted. The patient should be monitored until complete resolution of the symptoms and treated as clinically indicated. Treatment or concomitant medication may include acetaminophen/paracetamol, antihistamine, IV saline, oxygen, bronchodilators, corticosteroids and vasopressors depending on the symptoms.

If the infusion is interrupted:

- In the event of a grade 1 IRR, upon resolution of symptoms, the infusion will resume at the same rate (the rate being used at the time that the IRR occurred).
- In the event of a grade 2 or grade 3 IRR, upon resolution of symptoms, the infusion will resume at one-half the previous rate and infusion rate escalation may resume at the increments and intervals described in Section 4.4.2.3.2 for RO5479599.
- In the event of a grade 4 IRR (which may include pulmonary or cardiac events) or an anaphylactoid reaction, the patient should receive aggressive treatment and be withdrawn from the study and should not receive further any study treatment.

Note: Patients with reactions indicating a true allergic reaction, e.g. a reaction occurring with the second or later RO5479599 infusion, should undergo special laboratory assessments including IgE and tryptase. If the results support an allergic origin (i.e. increased tryptase levels and/or increased IgE levels, see also Section 4.6.1.8 Special Safety Assessments), the reaction should be reported as allergic reaction or anaphylactic reaction as appropriate, and patients should not be re-challenged with RO5479599.

Cardiac Safety

All patients must have a baseline LVEF ≥ 50%. LVEF will be monitored regularly according to the Schedule of Assessments (Appendix 1) and will be analyzed centrally. If an investigator is concerned that an AE may be related to cardiac dysfunction, an additional LVEF measurement should be performed. Pertuzumab, paclitaxel and RO5479599 will be discontinued in any patient who develops clinical signs and symptoms suggesting CHF, with the diagnosis confirmed by a suggestive chest X-ray and a drop in LVEF by echocardiography. CHF should be treated and monitored according to standard medical practice.

At present, there are inadequate data available to assess the prognostic significance of asymptomatic drops of LVEF. However, to ensure the safety of patients in the trial,

pertuzumab must be discontinued in all patients, for whom a drop of LVEF to a value lower than 40% is documented and confirmed with a repeat assessment within 3 weeks of the first assessment, using the same assessment method. For patients whose LVEF drops to values ≤ 45% (50% is required for entry into the study), the decision to stop or continue study treatment is based on the algorithm shown in Figure 3.

Figure 3 Asymptotic decline in LVEF: Algorithm for Continuation and Discontinuation of Pertuzumab Based on LVEF Assessments

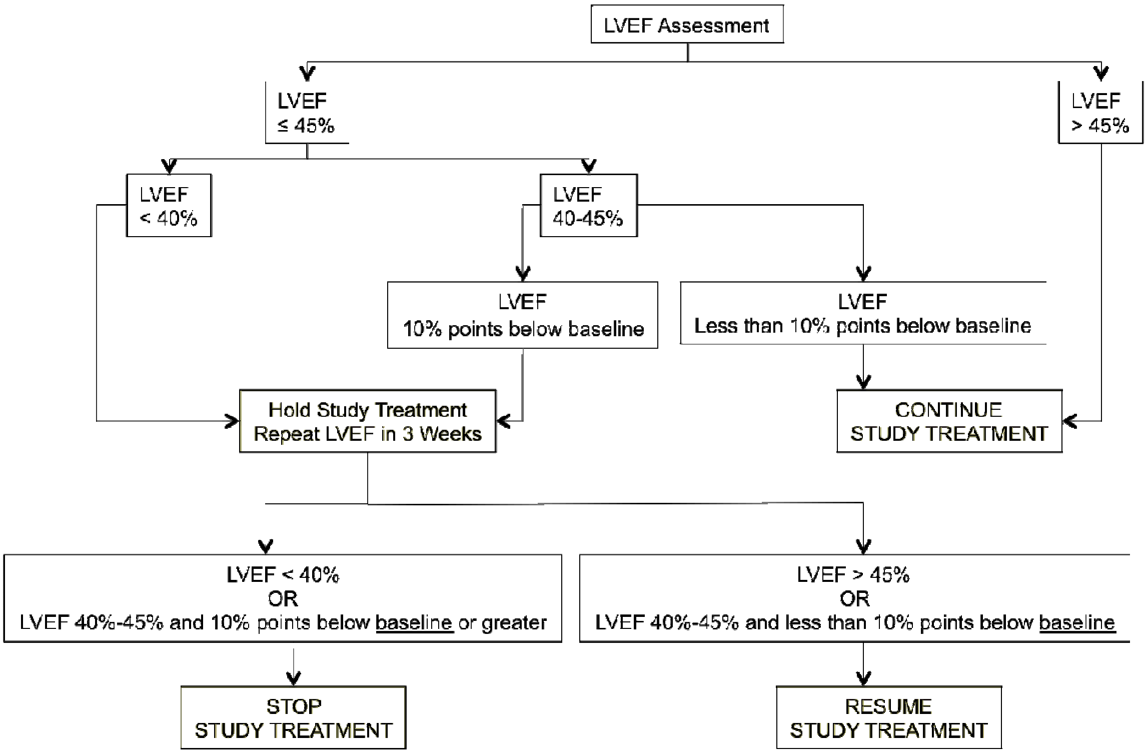

The incidence of CHF will also be recorded throughout the study.

See Appendix 3 for details of the NYHA classification and left ventricular systolic dysfunction NCI-CTCAE version 4.0 grading.

4.4.3 Additional Required Medication

The administration of pre-medication for paclitaxel, pertuzumab and RO5479599 is described in Sections 4.4.2.1.1, 4.4.2.2.1 and 4.4.2.3.1, respectively.

4.4.4 Investigational Medicinal Product Accountability

All investigational medicinal products (IMPs) required for completion of this study (RO5479599, pertuzumab, paclitaxel) will be provided by the sponsor. The investigational site will acknowledge receipt of IMPs, to confirm the shipment condition and content. Any damaged shipments will be replaced.

The investigator is responsible for the control of drugs under investigation. Adequate records of the receipt (e.g., Drug Receipt Record) and disposition (e.g. Drug Dispensing Log) of the study treatment must be maintained. The Drug Dispensing Log must be kept current and should contain the following information:

- The identification of the patient to whom the study treatment was dispensed (for example eCRF number, patient initials and date of birth).
- All records and drug supplies must be available for inspection by the Roche Monitor (at every monitoring visit).

IMPs will either be disposed of at the study site according to the study site's institutional standard operating procedure or returned to the sponsor with the appropriate documentation. The site's method of IMP destruction must be agreed upon by the sponsor. Local or institutional regulations may require immediate destruction of used IMP for safety reasons. In these cases, it may be acceptable for investigational study site staff to destroy dispensed IMP before a monitoring inspection provided that source document verification is performed on the remaining inventory and reconciled against the documentation of quantity shipped, dispensed, returned, destroyed and provided that adequate storage and integrity of drug has been confirmed. Written authorization must be obtained from the sponsor at study start up before destruction.

The site must obtain written authorization from the sponsor before any IMP is destroyed, and IMP destruction must be documented on the appropriate form.

Written documentation of destruction must contain the following:

- Identity (batch numbers) of investigational product(s) destroyed
- Quantity of investigational product(s) destroyed
- Date of destruction
- Method of destruction
- Name and signature of responsible person (or company) who destroyed investigational products(s)

Accurate records of all IMPs received at, dispensed from, returned to and disposed of by the study site should be recorded on the Drug Inventory Log.

4.4.5 Assessment of Compliance

Accountability and patient compliance will be assessed by maintaining adequate study treatment dispensing records. The investigator is responsible for ensuring that dosing is administered in compliance with the protocol. Delegation of this task must be clearly documented and approved by the investigator.

Since the administration of paclitaxel, pertuzumab and RO5479599 will occur under medical supervision, compliance with dosing is not expected to present a problem.

4.4.6 Post-Trial Access to RO5479599, Pertuzumab and Paclitaxel

Patients will be treated until disease progression, unacceptable toxicities, consent withdrawal or death. No post-trial access to study treatment will be available.

4.5 PROHIBITED THERAPY

Patients should be treated for all concomitant medical conditions and AEs according to accepted standards of medical care at the discretion of the investigator. The following treatments are not permitted during the study:

- Cytotoxic chemotherapy agents other than paclitaxel.
- Radiotherapy (as per inclusion criteria).
- Immunosuppressive therapy and chronically administered glucocorticoids (high dose is considered as > 20 mg of dexamethasone a day [or equivalent)] for > 7 consecutive days) as per exclusion criteria.
- Other systemic anti-neoplastic agents and targeted therapies.

If any of these therapies are needed, the patient will be considered to have evidence of progressive neoplastic disease and have experienced treatment failure with study treatment.

All concomitant treatments must be documented in the eCRF.

4.6 STUDY ASSESSMENTS

4.6.1 Description of Study Assessments

All examinations listed below will be performed according to the Schedule of Assessments outlined in Appendix 1.

4.6.1.1 Medical History and Demographic Data

Medical history, previous and concomitant medication and demographic data will be measured as specified in the Schedule of Assessments (Appendix 1).

Medical history describes clinically significant diseases, cancer history (including prior cancer therapies and procedures) and all medications (e.g., prescription drugs, over-the-counter drugs, herbal or homeopathic remedies, nutritional supplements) used by the patient within 6 months previous to informed consent or any ongoing clinically significant diseases.

Demographic data will include age, sex, and self-reported race. To explore any potential influence of race on PK and PD, information on race will be collected in the eCRF.

4.6.1.2 Physical Examinations

Physical examination data will be measured as specified in the Schedule of Assessments (Appendix 1).

A complete physical examination should include an evaluation of the head, eyes, ears, nose, and throat, and the cardiovascular, dermatological, musculoskeletal, respiratory, gastrointestinal, genitourinary and neurological systems.

Any abnormality identified at baseline should be recorded on the General Medical History and Baseline Conditions eCRF.

At subsequent visits (or as clinically indicated), limited, symptom-directed physical examinations should be performed. Changes from baseline abnormalities should be recorded in patient's notes. New or worsened clinically significant abnormalities should be recorded as AEs on the Adverse Event eCRF.

4.6.1.3 Vital Signs

Blood pressure, pulse rate and body temperature will be recorded at the time points specified in the Schedule of Assessments (Appendix 1).

Blood pressure and pulse rate should be obtained in a quiet room at a comfortable temperature, with the patient's arm unconstrained by clothing or other material. All measurements will be obtained from the same arm and, with the same cuff size, using an automatic instrument with a digital readout, throughout the study (the "ideal" cuff should have a bladder length that is 80% and a width that is at least 40% of arm circumference [a length-to-width ratio of 2:1]). The automatic cuff should be placed on the designated arm prior to dosing. The patient should be asked to remove all clothing that covers the location of cuff placement. The individual should be comfortably seated, with the legs uncrossed, and the back and arm supported, such that the middle of the cuff on the upper arm is at the level of the right atrium (the mid-point of the sternum). After the patient has been resting in a supine position for at least 5 minutes, blood pressure and pulse rate will be obtained.

For vital signs only the screening, pre-dose and Fup visit/early discontinuation visit assessments and any abnormalities will be recorded in the eCRF.

Medically significant findings that are present prior to the start of study treatment must be recorded in the Medical History eCRF with "ongoing" ticked. Medically significant findings made after start of study treatment or worsening of those reported at baseline and which meet the definition of an AE (see Section 5.1.1) must be recorded as an AE in the eCRF.

4.6.1.4 ECOG Performance Status

ECOG performance status (PS) will be classified as specified in Appendix 5 and measured at the timepoints specified in the Schedule of Assessments (Appendix 1).

PS will be measured using the ECOG Performance Status Scale. PS will be assessed with each physical exam. It is recommended, where possible, that a patient's PS will be assessed by the same person throughout the study.

4.6.1.5 Electrocardiograms

Triplicate ECGs (i.e. three useful ECGs without artifacts) will be recorded at screening, prior to infusion and at the end of the infusion on Day 2 of Cycle 1 and Day 1 of Cycle 3 and every 9 weeks (e.g. Cycle 3 Day 21) and at the final visit for all patients as specified in the Schedule of Assessments (Appendix 1).

Whenever possible, the same brand/model of a standard high-quality, high-fidelity electrocardiograph machine equipped with computer-based interval measurements should be used for each patient. The conditions should be as close as possible to pre-dose time points; this includes but is not limited to food intake, activity level, stressors and room temperature.

To minimize variability, it is important that patients be in a supine position for ≥ 10 min prior to each ECG evaluation. Body position should be consistently maintained for each ECG evaluation to prevent changes in heart rate. Environmental distractions (e.g., television, radio, conversation) should be avoided during the pre-ECG resting period and during ECG recording. ECGs should be performed prior to any scheduled vital sign measurements and blood draws. In some cases, it may be appropriate to repeat abnormal ECGs to rule out improper lead placement as contributing to the ECG abnormality.

For safety monitoring purposes, the investigator or designee must review, sign, and date all ECG tracings. Paper or electronic copies will be kept as part of the patient's permanent study file at the site. If considered appropriate by Roche, ECGs may be analyzed retrospectively at a central laboratory.

The following parameters will be obtained and recorded in the eCRF: PQ (PR), QRS, QT, and heart rate. RR, and QTcF (Fridericia's correction) will be calculated internally by the sponsor. T- and U-waves will be assessed by the investigator over all ECGs recorded at a given time point. T-waves should be captured as normal or abnormal and U-waves should be captured as absent/normal or abnormal.

Abnormal findings will be noted for clinical significance. A copy of each ECG should be printed at the bedside and retained at the site.

QTc will be calculated and reported using the Fridericia correction formula ($QTcF = QT_i / [RR_i^{1/3}]$, the QT interval divided by the cube root of the heart rate) by Roche. QTcF interval while on treatment should be evaluated for change from screening assessment. The average of the 3 screening ECG QT intervals (using QTcF) should be used to determine eligibility. If an increase of ≥ 60 ms in QTcF and/or QTcF ≥ 501 ms for males

or ≥ 521 ms for females is noted at ANY time point, further participation of this patient in this trial will be discussed between investigators and sponsor, and the decision will be made whether this patient should be withdrawn from this study and should be referred to a cardiologist for further evaluation and management.

Additional ECGs are ONLY REQUIRED as clinically indicated, e.g. there is evidence of QT prolongation on previous ECG assessments.

4.6.1.6 Echocardiography

LVEF assessments will be performed within 28 days prior to the first dose of study treatment and every 3 treatment cycles (9 weeks) by means of echocardiogram. Transthoracic echocardiogram (TTE) will be performed according to the standard practice of the investigational site at the time points indicated in the Schedule of Assessments (Appendix 1). This may be further repeated at the investigator's discretion if there are signs or symptoms of cardio-toxicity. TTE will be used to monitor the cardiac parameters of function, i.e. LVEF. The same method should be applied in the individual patient throughout the study. Preferably the same cardiologist should read and report the outcome to minimize variability in results. Copies of all echocardiograms performed on patients who experience a ≥ 20% decrease in LVEF from baseline and whose cardiac ejection fraction is below 50% may require additional assessments (see Figure 3). The institution's lower limit of normal may additionally be required by the sponsor for review and thus be kept as part of the patient's permanent study file at the site.

All echocardiograms must be recorded on videotape. All videotapes recorded at baseline and during the study and any unscheduled LVEF assessments performed to confirm observed LVEF drops, or in patients with signs and symptoms of cardiac dysfunction, will be forwarded for independent review.

4.6.1.7 Laboratory Assessments

Reference ranges for the study laboratory parameters must be supplied to the sponsor before the study starts. Laboratory safety tests shall be collected at time points specified in the Schedule of Assessments (Appendix 1).

Additional blood samples may be taken at the discretion of the investigator if the results of any test fall outside the reference ranges, or clinical symptoms necessitate additional testing to monitor patient safety. Where the clinical significance of abnormal laboratory values is considered uncertain, screening laboratory safety tests may be repeated to confirm eligibility.

Hematology, biochemistry and urinalysis will be done prior to study treatment infusion as part of regular safety assessments at time points as indicated in Schedule of Assessment (Appendix 1). Unscheduled hematology, biochemistry, urinalysis and coagulation may be obtained in patients who develop IRRs **or diarrhea or electrolyte**

imbalances and captured in the eCRF. Blood samples will be collected for the following clinical laboratory tests:

- Hematology (leucocytes, erythrocytes, hemoglobin, hematocrit, platelets, differential count [neutrophils, eosinophils, basophils, monocytes, lymphocytes, other cells such as non-hematological or atypical cells if present].
- Serum chemistry (aspartate aminotransferase [AST], alanine aminotransferase [ALT], GGT, bilirubin, alkaline phosphatase [ALP], LDH, albumin, creatinine, creatinine clearance [by Cockroft-Gault formula, see Appendix 4], total protein, sodium, chloride, calcium, phosphate, potassium, magnesium, glucose, urea, total and direct bilirubin).
- Dipstick for pH, glucose, blood, protein, ketones and bilirubin. If there is a clinically significant positive result (i.e. confirmed by a positive repeated sample), urine will be sent to the laboratory for microscopy and culture. If there is an explanation for the positive dipstick result, e.g. menses, it should be recorded, and there is no need to perform laboratory for microscopy and culture.
- Coagulation (PT/INR, PTT)
  In case of IRR or suspected dissiminated intravascular coagulation additional coagulation parameters, i.e. antithrombin III, fibrinogen, fibrin degradation products, prothrombin time, D-dimer may be assessed according to clinical judgment.
- Pregnancy test:
  All women of childbearing potential (including those who have had a tubal ligation) will have a serum pregnancy test at screening and at the follow-up visit.

4.6.1.8 Special Safety Assessments

Human anti-human antibodies: Sera for RO5479599 HAHA will be obtained prior to each infusion of RO5479599 and sera for pertuzumab HAHA will be obtained prior to each infusion of pertuzumab throughout all treatment cycles and on day 28 Follow-up (Fup) visit/early discontinuation visit (Appendix 2). A further HAHA analysis will be conducted at Fup visit on Day 42 to 45 for patients who are not entering another clinical trial or receiving further anti-tumor treatment.

HAHA samples are derived from splitting the PK samples into 2 aliquots (one aliquot each for PK and HAHA) of approximately equal volumes at these time points.

An additional sample for HAHA assessment will be taken in patients with clinical signs of immune complex-mediated toxicities.

For sampling procedures, storage conditions and shipment instructions, see the Sample Handling and Logistics Manual.

Leukocyte immunophenotyping: Blood for the assessment of leukocyte immunophenotyping includes the assessment of T cells (CD3, CD4, CD8), B cells (CD19), NK cells (CD3, CD16/56) and monocytes/macrophages (CD14, CD45) and will

be carried out throughout the treatment cycles. For collection time points please see the Schedule of Assessments (Appendix 1).

IgE and tryptase: For patients who experience an IRR ≥ grade 2 for the first time with the 2nd or subsequent RO5479599 infusion tryptase and IgE will be analyzed from an additional blood sample.

Organ biopsy: If a biopsy e.g. from colon or kidney is taken from a patient for a routine clinical assessment this sample could be sent on to Roche for further safety and toxicity analyses if the patient consented.

4.6.1.9 Pharmacokinetic Assessments

All PK samples will be collected from the arm (limb) which is not being used to administer the infusion.

PK assessments are performed during each cycle (see Specific Schedule of Assessments for PK and HAHA, Appendix 2). At each scheduled time point, approximately 2 mL of venous blood will be collected in a serum blood collection tube.

The date and time of each sample collection will be recorded. Additional unscheduled PK samples (up to 5 per patient) may be obtained if additional PK information is required to better characterize safety and PK. If multiple samples are drawn at a given time point, PK sample should take precedence (RO5479599, followed by pertuzumab, followed by paclitaxel).

Based on real patient data, the sample collection may be stopped or the sample schedule (timepoints) may be modified for improved data collection without an increase in number of samples.

If a patient discontinues/withdraws from the study before the subsequent cycle, then the PK sampling schedule for the subsequent cycle should be applied where feasible at the time of study discontinuation/withdrawal.

In case of dose delay due to quality of life-impairing diarrhea of Grade 2 or diarrhea of Grade ≥3 additional samples should be taken if possible at any clinical visit throughout the dose delay (e.g. together with the additional weekly biochemistry assessment) to measure RO5479599, pertuzumab and paclitaxel concentrations. If feasible, a PK sample should also be taken as close to the time of onset of the diarrhea as possible and at the time the diarrhea has resolved. If the patient resumes dosing then the PK sampling schedule for the subsequent cycle should be applied.

The samples will be stored for up to 6 months from the date of the finalization of the Bioanalytical Reports, after which time the Sponsor will confirm to the bioanalytical laboratory that the samples can be destroyed.

4.6.1.10 Exploratory Pharmacodynamic and Response Prediction Biomarker Assessments

These specimens will be used for research purposes to identify biomarkers useful for predicting and monitoring response to [drug] treatment, identifying biomarkers useful for predicting and monitoring [drug] safety, assessing PD effects of [drug] treatment, and investigating mechanisms of therapy resistance. This will include the assessment of markers associated with the PD activity of study treatment by analyzing NK lymphocyte functional assessment via NK cell activation, doing leukocyte immunophenotyping (see Schedule of Assessments [Appendix 1] for timepoints). Additional markers may be measured in case a strong scientific rationale for these analyses develops.

Data from non-clinical experiments further suggest that co-expression of certain molecules (e.g. cMET, heregulin, IGF1R) affect the responsiveness to HER3-targeted treatments in a way that they by-pass the inhibitory effect resulting in constant tumor proliferation. In addition, the inflammatory tumor microenvironment of BC could impact the response to drug therapy and therefore might be investigated with regards to potential response prediction markers (e.g. macrophage, NK cell or T-cell infiltration). Depending on the tissue availability and based on the emerging data on translational research in the field of BC, additional markers related to HER3 and BC may be included.

Biomarker samples will be stored for up to 2 years, for the protocol assessments defined above, unless otherwise indicated. Samples will be destroyed no later than 2 years after the date of final closure of the clinical database, unless regulatory authorities require specimens to be maintained for a longer time period. Archival tumor blocks will be returned.

Based on continuous analysis of the data in this study and other studies, any sample type not considered to be critical for safety may be stopped at any time if the data from the samples collected does not produce useful information.

4.6.1.10.1 Tumor Biopsies

Mandatory tumor biopsies (formalin fixed and fresh frozen) will be taken prior to the first study treatment infusion to assess the eligibility of the patient based on HER3 protein expression and HER2 status assessment analyzing protein expression and gene amplification in parallel as mentioned in Section 4.2.2. Centrally confirmed HER3 protein expression and HER2 status assessment on a freshly taken pretreatment biopsy (FFPET) is an eligibility criterion for study entry.

A second, optional tumor biopsy may be taken at disease progression/relapse at the discretion of the investigator.

Collection of tumor biopsies will be guided by ultrasound or CT scan using an 18 gauge needle to provide cores of at least 20 mm in length. At least 2 core biopsies will be obtained. The first core biopsy will be fixed in formalin and embedded into paraffin

(FFPET) and the second biopsy will be fresh frozen in liquid nitrogen (FF). For superficial lesions a punch biopsy can be performed instead if an adequately deep specimen can be warranted and including a representative amount of tumor cells. Therefore, the punch biopsy should have a diameter of ≥ 2 mm at least. Fine needle aspirates (FNAs) are not acceptable for this trial. To ensure that all relevant biomarkers related to the mode of action of study treatment (i.e., HER pathways and ADCC) can be addressed a third and fourth tumor biopsy per time point might be collected if feasible and safe for the patient. Details on preservation and processing of potential additional tumor biopsies will be given in the Lab Manual.

Biopsy instructions, sample handling and shipping will be provided in detail in the Laboratory Manual. In case of low quantity of biopsy material a list of a prioritized marker panel will be provided to the analysis laboratory.

FFPET biopsy samples and/or FF biopsy samples will be analyzed for:

- Molecules relevant to HER signaling or alternative pathways, e.g. HER3, EGFR, HER2, heregulin or cMET.
- ADCC-related markers like CD16, CD3, CD56, CD3.
- Molecules describing inflammatory tumor microenvironment, e.g. macrophage subtyping.
- Gene expression analysis to investigate signaling molecules which are modulated after HER3-targeted treatment, e.g., HER1, HER2, HER3, HER4.
- Assessment of tumor-specific mutations including but not limited to KRAS, PIK3CA, PTEN, EGFR, HER4, cMET.

Depending on emerging markers in BC research or related to HER-signaling, which might be identified during the course of the study, the biomarker approach might be adapted.

Biopsies should be performed according to institutional guidelines, e.g., ensuring adequate coagulation status and platelet count.

Residual tissue material (slides, extracts, on-study blocks, etc.) will be destroyed within 2 years after the date of final closure of the clinical database.

4.6.1.10.2 Archived primary tumor samples

Submission of primary archival tumor block, fixed in 10% neutral buffered formalin and embedded into paraffin (historical tumor sample), is not a mandatory requirement, but is highly encouraged if available. When submitted to the central laboratory, this tumor sample will be used to cut sections for subsequent biomarker analysis as described under Section 4.6.1.10.1. Comparing molecular patterns from primary archived tumor samples with freshly taken tumor biopsies, representing the respective disease state,

might help to better understand how markers like HER3, HER2 or EGFR and immune cell infiltration develop over time and may change under treatment.

To protect tissue against oxidation and to allow IHC analysis in large batches an archived tumor block would be preferred. Any left-over tissue blocks will be sent back to the investigator site after completion of the study.

For sampling procedures, storage conditions and shipment instructions, see the Sample Handling and Logistics Manual.

4.6.1.10.3 Exploratory Tissue Biomarker Assessments

Exploratory biomarker assessments include, but are not limited to:

HER3, HER2, EGFR and heregulin: Activation of HER3 can be induced via heterodimerization with other HER-family members (EGFR, HER2 or HER4) after ligand (heregulin) binding which results in receptor phosphorylation or via activation of downstream signaling preferred via the PI3K/AKT signaling cascade.

Gene expression profiling: Gene expression analysis is done by quantitatively assessing the RNA amount related to the patient's genes and is indicative of the activity of these genes. Gene expression analysis can be used to quantitatively assess gene transcripts related to pathways of anticipated biological relevance (HER3 signaling and other pathways that could bypass the HER3 pathway such as the EGFR and cMET pathway), which can lead to the discovery of novel interactions, information dependencies and regulatory relationships of markers of interest.

cMET: cMET has been considered as an escape mechanism for EGFR-targeting small molecule inhibitors in lung tumors [16, 54]. It is speculated that cMET gene amplification and/or cMET overexpression lead to a MET-dependent activation of HER3 via phosphorylation, which results in a sustained PI3K and Akt pathway activation and thus by-passing EGFR inhibition. Generally speaking it might be expected that cMET interaction with other receptor tyrosine kinases, e.g. HER-family members, can be important in several pathological settings [55].Therefore, monitoring the co-expression of HER3 and cMET in certain tumor entities might help to better understand the response to targeted therapies.

Immune cell infiltration into tumor tissue/inflammatory tumor microenvironment: RO5479599 exhibits increased binding affinity for both low and high affinity variants of FcγRIIIa that are expressed on NK cells, macrophages and monocytes. RO5479599 demonstrates significantly improved cell killing over a non-glycoengineered anti-HER3 antibody in ADCC-based assays. In an orthotopic lung cancer xenograft model, mice treated with RO5479599 survived longer than those treated with vehicle or with the non-glycoengineered antibody. As such, RO5479599 has the potential to show improved ADCC in humans with clinical activity in patients whose tumors express HER3 protein by recruiting NK cells or macrophages to HER3 positive tumors. Especially with regards to

immunosuppression there is recent evidence that tumor-associated macrophages may control other immune cell infiltrates e.g., tumor-infiltrating T cells.

Mutation analyses: Molecular studies of BC have focused on whole exome sequencing recently, identifying known and unknown somatic mutations, point mutations or insertions/deletions (indels). In addition to previously describe mutations (e.g., PIK3CA, PTEN, AKT1, TP53) a number of novel significantly mutated genes were identified, among them protein tyrosine phosphatases and transcription factors [56]. As HER3-HER2 dimer mediated signaling involves a variety of downstream protein phosphatases and kinases, mutation analyses doing whole exome sequencing might reveal markers, that can be correlated to HER3- and HER2-targeted treatment in the population being analyzed in this study.

Depending on the tissue availability and based on the emerging data on translational research in the field of BC, additional markers related to HER2 and HER3, as well as BC may be included. In addition, new diagnostic assays or technologies for sample analyses might be implemented, if they help to address correlation to drug response.

4.6.1.10.4 Blood Sampling

Blood samples for exploratory analysis will be obtained. Please see PD assessment schedule for PD and exploratory biomarker time points (Appendix 1) for PD and exploratory biomarkers.

Blood as surrogate tissue in the dose escalation and extension phase will be collected to determine the PD effects of RO5479599 and assess potential response predictors.

Exploratory biomarker assessments will include but is not limited to:

- Assessment of immune effector profiling (leukocytes immunophenotyping using FACS and monocyte/macrophage profile [for monocyte macrophage these markers include, but are not limited to CD14, CD16, CD45]) (see Appendix 1 for collection time points) to assess PD changes due to FC-gamma related immune mechanisms pre and post treatment with RO5479599.
- NK lymphocyte functional assessment via NK cell activation measured by CD107a to assess PD changes due to FC-gamma related immune mechanisms pre and post treatment with RO5479599.
- FcγR polymorphisms (clinical genotyping): As the RO5479599 molecule is glyco-engineered it is expected to have a higher binding affinity to FcγRIIIA receptor variants expressed on human immune effector cells such as NK cells, monocytes and macrophages. Two functional FCGR gene polymorphisms have been identified that may affect the killing function of immune effector cells. Several studies with rituximab or trastuzumab have correlated these polymorphisms with clinical efficacy. Cartron et al. reported that FcγRIIIA-V158F polymorphism was associated with tumor response in follicular non-Hodgkin's lymphoma patients with first-line rituximab treatment [20] and Musolino et al. reported on 54 clinical samples that

single nucleotide polymorphisms (SNPs) in the FCGR3A and 2A genes seem to have an impact on the clinical efficacy of trastuzumab in patients with HER2-positive MBC [21].Therefore, a single 3 mL whole blood sample will be taken at baseline from all patients for DNA extraction and assessment of FcγRIII and FcγRII polymorphisms. Blood samples for clinical genotyping may be analyzed during or at the end of the study and will be destroyed after completion of all analysis. Data arising from this study will be subject to the same confidentiality as the rest of the data for this study. For sampling procedures, storage conditions and shipment instructions see Sample Handling and Logistics Manual.

- Carcinoembryonic antigen (CEA) and Cancer Antigen 15-3 (CA15-3) are frequently used tumor markers in BC. These tumor markers will be assessed on an exploratory basis at the site. The same assay/platform should be used to assess CEA and CA 15-3 throughout the treatment cycles.

4.6.1.10.5 Total Blood Loss

Blood loss over screening, Cycles 1 to 8 and the 28-day follow-up visit totals up to approximately 480 mL. Up to approximately 150 mL of blood will be drawn from each patient during screening and Cycle 1, with the remainder, up to approximately 330 mL of blood (assuming 8 Cycles) will be drawn from every patient during the following cycles to assess clinical laboratory measurements, special safety assessments, determine serum/plasma concentrations and assess PD and exploratory biomarkers.

4.6.1.11 Disease-specific Assessments

Tumor and Response Evaluations

Tumor status will be evaluated according to the RECIST criteria (Version 1.1 [59]) for patients with solid tumors. Ultrasound and X-rays are not acceptable for monitoring target lesions. For each patient, the same method of assessment and the same technique must be used to evaluate each lesion throughout the entire study. If more than one method is used, select the most accurate method according to RECIST when recording data.

The extent of neoplastic disease will be determined by reproducible radiographic techniques, preferably MRI or CT scan. All known and suspected areas of neoplastic disease will be evaluated as deemed appropriate by the investigator. Consistency of consecutive CT scans or MRIs should be ensured during all assessments for each patient, with the same technique being used for evaluating lesions throughout the treatment period (use of spiral CT or MRI is required for baseline lesions < 20 mm and must be documented in medical records and used consistently throughout the study). The use of oral and IV contrast should, as long as it is clinically possible, be kept consistent. Tumor measurements should be made by the same investigator/radiologist for each patient during the study to the extent that this is feasible. In case of clinically measurable superficial (such as skin) lesions, repeated photographs should be used to document tumor response. These photos must include a ruler for documentation purposes. Patients with known or suspected bone metastases should undergo

radionuclide bone scanning.

It has been shown that molecularly targeted agents do not always induce tumor shrinkage, but sometimes only tumor stabilization [61]. However, criteria such as RECIST reduce the information by classifying treatment effect into only four categories (complete response, partial response, stable disease and progressive disease). Therefore, an assessment of tumor growth kinetics will be made by comparing post treatment scans with the last available pre-study scan, if available. By this means a potential benefit for patients irrespective of disease progression according to RECIST could be analyzed.

Schedule of Tumor Assessment

Baseline total tumor burden must be assessed within a maximum of 14 days before first dose of study treatment. Follow-up tumor evaluations will be performed every 9th week (±7 days) for the q3w schedule starting with the first dose of study treatment. All tumor assessments after screening will be done within ±7 days of the scheduled assessment and at the 28-day follow-up visit. Patients with previously documented tumor progression will not require additional tumor reassessment at the follow-up visit. If a patient inadvertently misses a prescribed tumor evaluation or a technical error prevents the evaluation, the patient may continue treatment until the next scheduled assessment, unless signs of clinical progression are present.

In cases where there is suspicion of progression before the next scheduled assessment, an unscheduled assessment is to be performed. It should also be considered that evidence of objective neoplastic progression (i.e., increase in the sum of the longest diameters of target lesions or the development of new lesions) may be preceded by other clinical factors. Therefore, in deciding to withdraw a patient from the study for progressive neoplastic disease, the investigator should consider all factors that may indicate progression, including, but not limited to, development of non-measurable evidence of new neoplastic disease, such as ascites or pleural effusion, deterioration in performance status, new symptoms, worsening of previously present symptoms such as pain, deterioration of appetite or weight, in order to not expose a progressing patient to unnecessary study treatment.

4.6.2 Timing of Study Assessments

4.6.2.1 Screening and Pre-treatment Assessments

Written informed consent for participation in the study must be obtained before performing any study-specific screening tests or evaluations. Informed Consent Forms for enrolled patients and for patients who are not subsequently enrolled will be maintained at the study site.

All screening and pre-treatment assessments must be completed and reviewed to confirm that patients meet all eligibility criteria. The investigator will maintain a screening log to record details of all patients screened and to confirm eligibility or record reasons for screening failure.

An Eligibility Screening Form (ESF) documenting the investigator's assessment of each screened patient with regard to the protocol's inclusion and exclusion criteria is to be completed by the investigator and kept at the investigational site.

Screening/baseline, study and follow-up assessments will be performed according to the Schedule of Assessments outlined in Appendix 1. This table provides the minimum requirements for the protocol. Additional clinical, radiological and laboratory assessments may be performed as clinically indicated, according to the investigator's clinical judgment.

Screening will be up to 28 days from signed informed consent until first administration of study treatment. The following will be assessed during screening:

- Inclusion/exclusion criteria
- Tumor assessment
- Tumor biopsies
- Clinical genotyping
- Medical history, including demographics
- Concomitant medication
- Protocol-mandated SAEs
- Complete physical examination and ECOG performance status (PS)
- Vital signs
- ECG (12-lead; see Section 4.6.1.5 for parameters)
- Laboratory evaluation to be performed by local laboratories including:
    - Hematology
    - Biochemistry
    - Urinalysis
    - Coagulation
    - Serum pregnancy test (in women with child bearing potential)
- Primary archival tumor block (if available)

4.6.2.2 Assessments during Treatment

Under no circumstances will patients who enroll in this study and have completed treatment as specified, be permitted to be allocated a new patient number and re-enroll in the study.

All assessments must be performed as per Schedule of Assessments (see Appendix 1). Assessments scheduled on the day of study treatment administration should be performed prior to administration of study treatment, unless otherwise noted in the Schedule of Assessments:

- Physical exam and ECOG performance status (EPS)
- Vital signs

- Triplicate 12-lead ECG
- 12-lead ECG
- Echocardiography
- Hematology and biochemistries (urinalysis only at the discretion of the investigator)
- Coagulation
- Tumor markers CEA and CA15-3
- Tumor assessment
- Serum for HAHA
- PK serum samples
- Leukocyte-immunophenotyping
- NK cell activation**. NK cell activation test will not be done for Extension Phase 2 Cohort patients.**
- AEs and concomitant medication

4.6.2.3 Assessments at Study Completion/Early Termination Visit/Follow-Up Assessments

All patients will attend a safety follow-up visit 28 days after receiving the last infusion of study treatment. Another PK/HAHA visit on Day 42 to 45 will be conducted for patients who are not entering another clinical trial or receiving further anti-tumor treatment. In the event of early discontinuation from the treatment, patients will also undergo a safety follow-up visit 28 days after receiving the last infusion of study treatment. This follow-up visit after early discontinuation can be brought forward to an earlier time point at the discretion of the investigator.

After the study completion/early termination visit, AEs should be followed as outlined in Sections 5.4 and 5.5.

All assessments must be performed as per Schedule of Assessments (see Appendix 1). The following will be assessed for the follow-up visit:

- Concomitant medication
- AEs
- Complete physical examination and ECOG performance status (PS)
- Vital signs
- ECG (12-lead; see Section 4.6.1.5 for parameters)
- Echocardiogram
- Tumor assessment
- PK and HAHA

- Laboratory evaluation to be performed by local laboratories including:
    - Hematology
    - Biochemistry
    - Urinalysis
    - Coagulation
    - Serum pregnancy test (in women with child bearing potential)

After study discontinuation, patients' survival status will be followed up for up to 2 years by regular phone calls.

4.6.2.4 Assessments at Unscheduled Visits

The following assessments could be done in case of an unscheduled visit:

- Echocardiography
- Plasma for IgE and tryptase
- Serum for HAHA and PK
- Tumor assessment (in case of suspicion of progression before the next scheduled assessment)
- **Biochemistry in case of electrolyte imbalances or Grade ≥2 diarrhea**

Please see Appendix 1 for assessments that are required to be performed in case of an unscheduled visit.

4.7 PATIENT, STUDY, AND SITE DISCONTINUATION

4.7.1 Patient Discontinuation

The investigator has the right to discontinue a patient from study treatment or withdraw a patient from the study at any time. In addition, patients have the right to voluntarily discontinue study treatment or withdraw from the study at any time for any reason. Reasons for discontinuation of study treatment or withdrawal from the study may include, but are not limited to, the following*:*

- Patient withdrawal of consent at any time.
- Any medical condition that the investigator or sponsor determines may jeopardize the patient's safety if she/he continues in the study.
- Investigator or sponsor determines it is in the best interest of the patient.

In the event that a patient decides to prematurely discontinue from the study, she/he should be asked if she/he can still be contacted for further information. The outcome of that discussion should be documented in both the medical records and in the eCRF.

When applicable, patients should be informed of circumstances under which their participation may be terminated by the investigator without the patient's consent. The investigator may withdraw patients from the study in the event of intercurrent illness, AEs, treatment failure after a prescribed procedure, lack of compliance with the study and/or

study procedures (e.g., dosing instructions, study visits) cure or any other reasons where the investigator feels it is in the best interest of the patient to be terminated from the study. In patients with clinical signs of hypersensitivity reactions or immune complex-mediated toxicities treatment will be immediately delayed and appropriate diagnostic measurements will be performed. Patients in whom HAHA formation is confirmed will be removed from the study. For patients in whom HAHA formation could not be confirmed the decision to remove the patient will be determined between the investigator and the sponsor (see Section 4.7.1) See also any stopping criteria listed under Section 3.2.2.1.

Reasons for withdrawal must be documented and explained to the patient. It is understood by all concerned that an excessive rate of withdrawals can render the study uninterpretable; therefore, unnecessary withdrawal of patients should be avoided. Should a patient decide to withdraw, all efforts should be made to complete and report the observations, particularly the follow-up examinations, as thoroughly as possible.

The investigator should contact the patient or a responsible relative either by telephone (followed by registered mail) or through a personal visit to determine as completely as possible the reason for the withdrawal. A complete final evaluation at the time of the patient's withdrawal should be made with an explanation of why the patient is withdrawing from the study. If the reason for removal of a patient from the study is an AE, the principal specific event must be recorded on the eCRF. The patient should be followed until the AE is resolved, if possible.

4.7.1.1 Discontinuation from Study Treatment

Patients must discontinue study treatment if they experience any of the following:

- Pregnancy
- Clinical signs and symptoms suggesting CHF.
- Symptomatic left ventricular dysfunction (NCI-CTCAE Version 4.0 Grade 3 or 4) with a drop in LVEF consistent with cardiac failure.

Details of discontinuation due to toxicity are given in Section 4.4.2.6.

Patients who discontinue study treatment prematurely will be asked to return to the clinic for a study completion/early termination visit (see Section 4.6.2.3) and may undergo follow‑up assessments (see Section 4.6.2.3). The primary reason for premature study treatment discontinuation should be documented on the appropriate eCRF. Patients who discontinue study treatment prematurely will not be replaced.

4.7.1.2 Withdrawal from Study

Every effort should be made to obtain information on patients who withdraw from the study. The primary reason for withdrawal from the study should be documented on the appropriate eCRF.

Patients will not be followed for any reason after consent has been withdrawn.

Patients who withdraw from the study within the DLT period will be replaced.

4.7.2 Study and Site Discontinuation

The sponsor has the right to terminate this study at any time. Reasons for terminating the study may include, but are not limited to, the following:

- The incidence or severity of AEs in this or other studies indicates a potential health hazard to patients.
- Patient enrollment is unsatisfactory.

The sponsor will notify the investigator if the study is placed on hold, or if the sponsor decides to discontinue the study or development program.

The sponsor has the right to replace a site at any time. Reasons for replacing a site may include, but are not limited to, the following:

- Excessively slow recruitment.
- Poor protocol adherence.
- Inaccurate or incomplete data recording.
- Non-compliance with the International Conference on Harmonisation (ICH) guideline for Good Clinical Practice.

5. ASSESSMENT OF SAFETY

5.1 SAFETY PARAMETERS AND DEFINITIONS

Safety assessments will consist of monitoring and recording AEs, including SAEs and non-serious AEs of special interest; measurement of protocol-specified safety laboratory assessments; measurement of protocol-specified vital signs, ECGs; and other protocol-specified tests that are deemed critical to the safety evaluation of the study.

Certain types of events require immediate reporting to the sponsor, as outlined in Section 5.3.5.12

5.1.1 Adverse Events

According to the ICH guideline for Good Clinical Practice, an AE is any untoward medical occurrence in a clinical investigation subject administered a pharmaceutical product, regardless of causal attribution. An AE can therefore be any of the following:

- Any unfavorable and unintended sign (including an abnormal laboratory finding), symptom, or disease temporally associated with the use of a medicinal product, whether or not considered related to the medicinal product.
- Any new disease or exacerbation of an existing disease (a worsening in the character, frequency, or severity of a known condition), except as described in Section 5.3.5.9.
- Recurrence of an intermittent medical condition (e.g., headache) not present at baseline.

- Any deterioration in a laboratory value or other clinical test (e.g., ECG, X-ray) that is associated with symptoms or leads to a change in study treatment or concomitant treatment or discontinuation from study treatment.
- AEs that are related to a protocol-mandated intervention, including those that occur prior to assignment of study treatment (e.g., screening invasive procedures such as biopsies).

5.1.2 Serious Adverse Events (Immediately Reportable to the Sponsor)

A SAE is any AE that meets any of the following criteria:

- Fatal (i.e., the AE actually causes or leads to death).
- Life threatening (i.e., the AE, in the view of the investigator, places the patient at immediate risk of death).

This does not include any AE that had it occurred in a more severe form or was allowed to continue might have caused death.

- Requires or prolongs inpatient hospitalization (see Section 5.3.5.10).
- Results in persistent or significant disability/incapacity (i.e., the AE results in substantial disruption of the patient's ability to conduct normal life functions).
- Congenital anomaly/birth defect in a neonate/infant born to a mother exposed to study treatment.
- Significant medical event in the investigator's judgment (e.g., may jeopardize the patient or may require medical/surgical intervention to prevent one of the outcomes listed above).

The terms "severe" and "serious" are not synonymous. Severity refers to the intensity of an AE (rated as mild, moderate, or severe, or according to a pre-defined grading criteria (e.g. NCI CTCAE criteria; see Section 5.3.3); the event itself may be of relatively minor medical significance (such as severe headache without any further findings).

Severity and seriousness need to be independently assessed for each AE recorded on the eCRF.

SAEs are required to be reported by the investigator to the sponsor immediately (i.e., no more than 24 h after learning of the event; see Section 5.3.7 for reporting instructions).

5.1.3 Non-Serious Adverse Events of Special Interest (Immediately Reportable to the Sponsor)

Non-serious AEs of special interest are required to be reported by the investigator to the sponsor immediately (i.e., no more than 24 h after learning of the event; see Section 5.3.7 for reporting instructions). AEs of special interest for this study include the following:

- Cases of an elevated ALT or AST in combination with either an elevated bilirubin or clinical jaundice, as defined in Section 5.3.5.6.

5.1.4 Pregnancy and Contraception

Women of childbearing potential (who have undergone surgical sterilization) and men with partners of childbearing potential should agree to use a highly effective form of contraception during the study treatment and for at least 6 months after the last dose of treatment. The Investigator will ensure that the method of contraceptive proposed is compatible with the medical status of the patient (e.g., non-hormonal contraceptive methods for breast cancer patients).

5.2 SAFETY PLAN

5.2.1 Management of Specific Adverse Events

For the treatment of RO5479599-, pertuzumab- and paclitaxel-related toxicities, please see Section 4.4.2.5 and 4.4.2.6.1.

LVEF: If LVEF is <40% or is 40 to 45% with a 10%-points or greater absolute decrease below the pretreatment value, withhold the study treatment and repeat the LVEF assessment within approximately 3 weeks. Discontinue study medication if the LVEF has not improved or has declined further, unless the benefits for the individual patient outweigh the risks. Treatment must be discontinued in all patients for whom a drop of LVEF to a value lower than 40% is documented and confirmed with a repeat assessment within 3 weeks of the first assessment, using the same assessment method (see algorithm Figure 3).

5.3 METHODS AND TIMING FOR CAPTURING AND ASSESSING SAFETY PARAMETERS

The investigator is responsible for ensuring that all AEs (see Section 5.2.1 for definition) are recorded on the Adverse Event eCRF and reported to the sponsor in accordance with instructions provided in this section and in Sections 5.4 to 5.6.

For each AE recorded on the Adverse Event eCRF, the investigator will make an assessment of seriousness (see Section 5.1.2 for seriousness criteria), severity (see Section 5.3.3), and causality (see Section 5.3.4).

5.3.1 Adverse Event Reporting Period

Investigators will seek information on AEs at each patient contact. All AEs, whether reported by the patient or noted by study personnel, will be recorded in the patient's medical record and on the Adverse Event eCRF.

After informed consent has been obtained but prior to initiation of study treatment, only SAEs caused by a protocol-mandated intervention should be reported (e.g., SAEs related to invasive procedures such as biopsies).

After initiation of study treatment, all AEs, regardless of relationship to study treatment, will be reported until up to 28 days after the last dose of study treatment. After this period, investigators should report any deaths, SAEs, or other AEs of concern that are believed to be related to prior treatment with study treatment (see Section 5.5).

5.3.2 Eliciting Adverse Event Information

A consistent methodology of non-directive questioning should be adopted for eliciting AE information at all patient evaluation time-points. Examples of non-directive questions include the following:

> "How have you felt since your last clinic visit?"
>
> "Have you had any new or changed health problems since you were last here?"

5.3.3 Assessment of Severity of Adverse Events

The AE severity grading scale for the NCI CTCAE (v4.0) will be used for assessing AE severity. Table 6 will be used for assessing severity for AEs that are not specifically listed in the NCI CTCAE.

Table 6 Adverse Event Severity Grading Scale

Grade	Severity
1	Mild; asymptomatic or mild symptoms; clinical or diagnostic observations only; or intervention not indicated
2	Moderate; minimal, local, or non-invasive intervention indicated; or limiting age-appropriate instrumental activities of daily living [a]
3	Severe or medically significant, but not immediately life-threatening; hospitalization or prolongation of hospitalization indicated; disabling; or limiting self-care activities of daily living [b,c]
4	Life-threatening consequences or urgent intervention indicated [d]
5	Death related to adverse event [d]

NCI CTCAE = National Cancer Institute Common Terminology Criteria for Adverse Events.

Note: Based on the NCI CTCAE (v4.0), which can be found at: http://evs.nci.nih.gov/ftp1/CTCAE/CTCAE_4.03_2010-06-14_QuickReference_8.5x11.pdf

[a] Instrumental activities of daily living refer to preparing meals, shopping for groceries or clothes, using the telephone, managing money, etc.

[b] Examples of self-care activities of daily living include bathing, dressing and undressing, feeding one's self, using the toilet, and taking medications, as performed by patients who are not bedridden.

[c] If an event is assessed as a "significant medical event" it must be reported as a SAE (see Section 5.3.7 for reporting instructions), per the definition of SAE in Section 5.1.2.

[d] Grade 4 and 5 events must be reported as SAEs (see Section 5.3.7 for reporting instructions), per the definition of SAE in Section 5.1.2.

5.3.4 <u>Assessment of Causality of Adverse Events</u>

Investigators should use their knowledge of the patient, the circumstances surrounding the event, and an evaluation of any potential alternative causes to determine whether or not an AE is considered to be related to the study treatment, indicating "yes" or "no" accordingly. The following guidance should be taken into consideration:

- Temporal relationship of event onset to the initiation of study treatment.
- Course of the event, considering especially the effects of dose reduction, discontinuation of study treatment or reintroduction of study treatment.
- Known association of the event with the study treatment or with similar treatments.
- Known association of the event with the disease under study.
- Presence of risk factors in the patient or use of concomitant medications known to increase the occurrence of the event.
- Presence of non-treatment-related factors that are known to be associated with the occurrence of the event.

For patients receiving combination therapy, causality will be assessed individually for each protocol-mandated therapy.

5.3.5 <u>Procedures for Recording Adverse Events</u>

Investigators should use correct medical terminology/concepts when recording AEs on the Adverse Event eCRF. Avoid colloquialisms and abbreviations.

Only one AE term should be recorded in the event field on the Adverse Event eCRF.

5.3.5.1 Diagnosis versus Signs and Symptoms

Infusion-Related Reactions

Administration of therapeutic antibodies my cause infusion-related reactions (IRRs) characterized by symptoms such as fever, chills, hypotension, shortness of breath, skin rash, headache, nausea, and/or vomiting. Such reactions typically occur during or shortly after an infusion, predominantly the first infusion. The incidence and severity typically decrease with subsequent infusions. Patients may also develop IgE-mediated hypersensitivity reactions to RO5479599. IRRs may be indistinguishable from an anaphylactic reaction.

Reactions to the administration of RO5479599 that are consistent with the description above should be captured as diagnosis and reported as <u>infusion-related reaction</u>. In addition, the individual signs and symptoms as well as the severity should be captured in a dedicated eCRF page.

<u>Note</u>: Patients with reactions indicating a true allergic reaction, e.g. a reaction occurring with the second or later RO5479599 infusion, should undergo special laboratory

assessments including IgE and tryptase. If the results support an allergic origin (i.e. increased tryptase levels and/or increased IgE levels, see also Section 4.6.1.8 Special Safety Assessments), the reaction should be reported as allergic reaction or anaphylactic reaction as appropriate.

Other Adverse Events

For AEs, a diagnosis (if known) should be recorded on the Adverse Event eCRF rather than individual signs and symptoms (e.g., record only liver failure or hepatitis rather than jaundice, asterixis, and elevated transaminases). However, if a constellation of signs and/or symptoms cannot be medically characterized as a single diagnosis or syndrome at the time of reporting, each individual event should be recorded on the Adverse Event eCRF. If a diagnosis is subsequently established, all previously reported AEs based on signs and symptoms should be nullified and replaced by one AE report based on the single diagnosis, with a starting date that corresponds to the starting date of the first symptom of the eventual diagnosis.

5.3.5.2 Adverse Events Occurring Secondary to Other Events

In general, AEs occurring secondary to other events (e.g., cascade events or clinical sequelae) should be identified by their primary cause, with the exception of severe or serious secondary events. However, medically significant AEs occurring secondary to an initiating event that are separated in time should be recorded as independent events on the Adverse Event eCRF. For example:

- If vomiting results in mild dehydration with no additional treatment in a healthy adult, only vomiting should be reported on the eCRF.
- If vomiting results in severe dehydration, both events should be reported separately on the eCRF.
- If a severe gastrointestinal hemorrhage leads to renal failure, both events should be reported separately on the eCRF.
- If dizziness leads to a fall and subsequent fracture, all three events should be reported separately on the eCRF.

All AEs should be recorded separately on the Adverse Event eCRF if it is unclear as to whether the events are associated.

5.3.5.3 Persistent or Recurrent Adverse Events

A persistent AE is one that extends continuously, without resolution, between patient evaluation time‑points. Such events should only be recorded once on the Adverse Event eCRF. The initial severity of the event should be recorded, and the severity should be updated to reflect the most extreme severity any time the event worsens. If the event becomes serious, the Adverse Event eCRF should be updated to reflect this.

A recurrent AE is one that resolves between patient evaluation time‑points and subsequently recurs. Each recurrence of an AE should be recorded separately on the Adverse Event eCRF.

5.3.5.4 Abnormal Laboratory Values

Not every laboratory abnormality qualifies as an AE. A laboratory test result should be reported as an AE if it meets any of the following criteria:

- Accompanied by clinical symptoms.
- Results in a change in study treatment (e.g., dosage modification, treatment interruption or treatment discontinuation).
- Results in a medical intervention (e.g., potassium supplementation for hypokalemia) or a change in concomitant therapy.
- Clinically significant in the investigator’s judgment.

It is the investigator’s responsibility to review all laboratory findings. Medical and scientific judgment should be exercised in deciding whether an isolated laboratory abnormality should be classified as an AE.

If a clinically significant laboratory abnormality is a sign of a disease or syndrome (e.g., alkaline phosphatase and bilirubin 5 times the upper limit of normal [ULN] associated with cholecystitis), only the diagnosis (i.e., cholecystitis) should be recorded on the Adverse Event eCRF.

If a clinically significant laboratory abnormality is not a sign of a disease or syndrome, the abnormality itself should be recorded on the Adverse Event eCRF, along with a descriptor indicating if the test result is above or below the normal range (e.g., “elevated potassium”, as opposed to “abnormal potassium”). If the laboratory abnormality can be characterized by a precise clinical term per standard definitions, the clinical term should be recorded as the AE. For example, an elevated serum potassium level of 7.0 mEq/L should be recorded as “hyperkalemia”.

Observations of the same clinically significant laboratory abnormality from visit to visit should not be repeatedly recorded on the Adverse Event eCRF, unless the etiology changes. The initial severity of the event should be recorded, and the severity or seriousness should be updated any time the event worsens.

5.3.5.5 Abnormal Vital Sign Values

Not every vital sign abnormality qualifies as an AE. A vital sign result should be reported as an AE if it meets any of the following criteria:

- Accompanied by clinical symptoms.
- Results in a change in study treatment (e.g., dosage modification, treatment interruption or treatment discontinuation).
- Results in a medical intervention or a change in concomitant therapy.
- Clinically significant in the investigator's judgment.

It is the investigator's responsibility to review all vital sign findings. Medical and scientific judgment should be exercised in deciding whether an isolated vital sign abnormality should be classified as an AE.

If a clinically significant vital sign abnormality is a sign of a disease or syndrome (e.g., high blood pressure), only the diagnosis (i.e., hypertension) should be recorded on the Adverse Event eCRF.

Observations of the same clinically significant vital sign abnormality from visit to visit should not be repeatedly recorded on the Adverse Event eCRF, unless the etiology changes. The initial severity of the event should be recorded, and the severity or seriousness should be updated any time the event worsens.

5.3.5.6 Abnormal Liver Function Tests

The finding of an elevated ALT or AST in combination with either an elevated total bilirubin or clinical jaundice in the absence of cholestasis or other causes of hyperbilirubinemia is considered to be an indicator of potential severe liver injury. Therefore, investigators must report as an adverse event the occurrence of either of the following:

- Treatment-emergent ALT or AST > 5 x ULN value in combination with total bilirubin > 2 x ULN (of which 35% is direct bilirubin)
- Treatment-emergent ALT or AST > 5 x ULN value in combination with clinical jaundice.

The most appropriate diagnosis or (if a diagnosis cannot be established) the abnormal laboratory values should be recorded on the Adverse Event eCRF (see Section 5.3.5.1) and reported to the sponsor immediately (i.e., no more than 24 h after learning of the event), either as a SAE or a non-serious AE of special interest (see Section 5.3.7).

5.3.5.7 Deaths

For this protocol, mortality is an efficacy endpoint. Deaths that occur during the protocol-specified AE reporting period (see Section 5.3.5.7) that are attributed by the investigator solely to progression of disease should be recorded only on the Study Completion/Early Discontinuation eCRF. All other on-study deaths, regardless of relationship to study treatment, must be recorded on the Adverse Event eCRF and immediately reported to the sponsor (see Section 5.3.5.7).

Death should be considered an outcome and not a distinct event. The event or condition that caused or contributed to the fatal outcome should be recorded as the single medical concept on the Adverse Event eCRF. Generally, only one such event should be reported. The term “sudden death” should only be used for the occurrence of an abrupt and unexpected death due to presumed cardiac causes in a patient with or without preexisting heart disease, within 1 h of the onset of acute symptoms or, in the case of an unwitnessed death, within 24 h after the patient was last seen alive and stable. If the cause of death is unknown and cannot be ascertained at the time of reporting, “unexplained death” should be recorded on the Adverse Event eCRF. If the cause of death later becomes available (e.g., after autopsy), “unexplained death” should be replaced by the established cause of death.

During post-study survival follow-up, deaths attributed to progression of cancer should be recorded only on the Survival Follow-up eCRF.

5.3.5.8 Pre-existing Medical Conditions

A pre-existing medical condition is one that is present at the screening visit for this study. Such conditions should be recorded on the General Medical History and Baseline Conditions eCRF.

A pre-existing medical condition should be recorded as an AE only if the frequency, severity, or character of the condition worsens during the study. When recording such events on the Adverse Event eCRF, it is important to convey the concept that the preexisting condition has changed by including applicable descriptors (e.g., “more frequent headaches”).

5.3.5.9 Lack of Efficacy or Worsening of Malignant Disease

Events that are clearly consistent with the expected pattern of progression of the underlying disease should not be recorded as AEs. These data will be captured as efficacy assessment data only. In most cases, the expected pattern of progression will be based on RECIST criteria. In rare cases, the determination of clinical progression will be based on symptomatic deterioration. However, every effort should be made to document progression using objective criteria. If there is any uncertainty as to whether an event is due to disease progression, it should be reported as an AE.

5.3.5.10 Hospitalization or Prolonged Hospitalization

Any AE that results in hospitalization or prolonged hospitalization should be documented and reported as a SAE (per the definition of SAE in Section 5.1.2), except as outlined below.

The following hospitalization scenarios are not considered to be SAEs:

- Hospitalization for respite care.
- Planned hospitalization required by the protocol (e.g. for study treatment administration or insertion of access device for study treatment administration).

- Hospitalization for a preexisting condition, provided that all of the following criteria are met:
  - The hospitalization was planned prior to the study or was scheduled during the study when elective surgery became necessary because of the expected normal progression of the disease.
  - The patient has not suffered an AE.
- Hospitalization due solely to progression of the underlying cancer.

5.3.5.11 Overdoses

Study treatment overdose is the accidental or intentional use of the drug in an amount higher than the dose being studied. An overdose or incorrect administration of study treatment is not an AE unless it results in untoward medical effects.

Any study treatment overdose or incorrect administration of study treatment should be noted on the Study Treatment Administration eCRF.

All AEs associated with an overdose or incorrect administration of study treatment should be recorded on the Adverse Event eCRF. If the associated AE fulfills serious criteria, the event should be reported to the sponsor immediately (i.e., no more than 24 h after learning of the event; see Section 5.3.7).

5.3.5.12 Immediate Reporting Requirements from Investigator to Sponsor

Certain events require immediate reporting to allow the sponsor to take appropriate measures to address potential new risks in a clinical trial. The investigator must report such events to the sponsor immediately; under no circumstances should reporting take place more than 24 h after the investigator learns of the event. The following is a list of events that the investigator must report to the sponsor within 24 h after learning of the event, regardless of relationship to study treatment:

- SAEs
- Non-serious AEs of special interest (i.e., drug-induced liver injury)
- Pregnancies

The investigator must report new significant follow-up information for these events to the sponsor immediately (i.e., no more than 24 h after becoming aware of the information). New significant information includes the following:

- New relevant signs and symptoms or any change in the diagnosis
- Relevant new diagnostic test results
- Change in causality based on new information
- Change in the event’s outcome, including recovery
- Additional narrative information on the clinical course of the event

Investigators must also comply with local requirements for reporting SAEs to the local health authority and IRB/EC.

5.3.6 Emergency Medical Contacts

To ensure the safety of study patients, an Emergency Medical Call Center Help Desk will access the Roche Medical Emergency List, escalate emergency medical calls, provide medical translation service (if necessary), connect the investigator with a Roche Medical Responsible, and track all calls. The Emergency Medical Call Center Help Desk will be available 24 h per day, 7 days per week. Toll-free numbers for the Help Desk and Medical Responsible contact information will be distributed to all investigators (see “Protocol Administrative and Contact Information & List of Investigators”).

5.3.7 Reporting Requirements for Serious Adverse Events and Non-Serious Adverse Events of Special Interest

For reports of SAEs and non-serious AEs of special interest (see Sections 5.1.2 and 5.1.3), investigators should record all case details that can be gathered on the Serious Adverse Reporting Form and forward this form to the SAE Responsible within 24 h.

5.3.8 Reporting Requirements for Pregnancies

5.3.8.1 Pregnancies in Female Patients

Female patients of childbearing potential will be instructed to immediately inform the investigator if they become pregnant during the study or within 6 months after the last dose of study treatment. A Clinical Trial Pregnancy Reporting Form should be completed by the investigator and submitted to the sponsor within 24 h after learning of the pregnancy. Pregnancy should not be recorded on the Adverse Event eCRF. The investigator should discontinue study treatment and counsel the patient, discussing the risks of the pregnancy and the possible effects on the fetus. Monitoring of the patient should continue until conclusion of the pregnancy.

5.3.8.2 Pregnancies in Female Partners of Male Patient

Male patients will be instructed through the Informed Consent Form to immediately inform the investigator if their partner becomes pregnant during the study or within 6 months after the last dose of study treatment. A Clinical Trial Pregnancy Reporting Form should be completed by the investigator and submitted to the sponsor within 24 h after learning of the pregnancy. Attempts should be made to collect and report details of the course and outcome of any pregnancy in the partner of a male patient exposed to study treatment. The pregnant partner will need to sign an Authorization for Use and Disclosure of Pregnancy Health Information to allow for follow-up on her pregnancy. Once the authorization has been signed, the investigator will update the Clinical Trial Pregnancy Reporting Form with additional information on the course and outcome of the pregnancy. An investigator who is contacted by the male patient or his pregnant partner may provide information on the risks of the pregnancy and the possible effects on the fetus, to support an informed decision in cooperation with the treating physician and/or obstetrician.

5.3.8.3 Abortions

Any spontaneous abortion should be classified as a SAE (as the sponsor considers spontaneous abortions to be medically significant events), recorded on the Adverse Event eCRF, and reported to the sponsor immediately (i.e., no more than 24 h after learning of the event; see Section 5.3.7).

5.3.8.4 Congenital Anomalies/Birth Defects

Any congenital anomaly/birth defect in a child born to a female patient or female partner of a male patient exposed to study treatment should be classified as a SAE, recorded on the Adverse Event eCRF, and reported to the sponsor immediately (i.e., no more than 24 h after learning of the event; see Section 5.3.7).

5.4 FOLLOW-UP OF PATIENTS AFTER ADVERSE EVENTS

5.4.1 Investigator Follow-Up

The investigator should follow each AE until the event has resolved to baseline grade or better, the event is assessed as stable by the investigator, the patient is lost to follow-up, or the patient withdraws consent. Every effort should be made to follow all SAEs considered to be related to study treatment or trial-related procedures until a final outcome can be reported.

During the study period, resolution of AEs (with dates) should be documented on the Adverse Event eCRF and in the patient’s medical record to facilitate source data verification. If, after follow-up, return to baseline status or stabilization cannot be established, an explanation should be recorded on the Adverse Event eCRF.

All pregnancies reported during the study should be followed until pregnancy outcome and reported according to the instructions provided in Section 5.3.8.

5.4.2 Sponsor Follow-Up

For SAEs, non-serious AEs of special interest, and pregnancies, the sponsor or a designee may follow up by telephone, fax, electronic mail, and/or a monitoring visit to obtain additional case details and outcome information (e.g., from hospital discharge summaries, consultant reports, autopsy reports) in order to perform an independent medical assessment of the reported case.

5.5 POST-STUDY ADVERSE EVENTS

At the study completion/early termination visit, the investigator should instruct each patient to report to the investigator any subsequent adverse events that the patient’s personal physician believes could be related to prior study treatment or study procedures.

The investigator is not required to actively monitor patients for AEs after the end of the AE reporting period (defined as 28 days after the last dose of study drug). However, the investigator should notify the sponsor of any death, SAE, or other AE of

concern occurring at any time after a patient has discontinued study participation **regardless of causality**. The sponsor should also be notified if the investigator becomes aware of the development of cancer or a congenital anomaly/birth defect in a subsequently conceived offspring of a patient that participated in this study.

The investigator should report these events to Roche Safety Risk Management on the Adverse Event eCRF. If the Adverse Event eCRF is no longer available, the investigator should report the event directly to Roche Safety Risk Management via telephone (see "Protocol Administrative and Contact Information & List of Investigators").

During post-study survival follow-up, deaths attributed to progression of malignant disease should be recorded only on the Survival Follow-up eCRF.

5.6 EXPEDITED REPORTING TO HEALTH AUTHORITIES, INVESTIGATORS, INSTITUTIONAL REVIEW BOARDS, AND ETHICS COMMITTEES

The sponsor will promptly evaluate all SAEs and non-serious AEs of special interest against cumulative product experience to identify and expeditiously communicate possible new safety findings to investigators, IRBs, ECs, and applicable health authorities based on applicable legislation.

To determine reporting requirements for single AE cases, the sponsor will assess the expectedness of these events using the following reference documents:

- RO5479599 Investigator's Brochure and Pertuzumab SmPC
- Local prescribing information for paclitaxel

The sponsor will compare the severity of each event and the cumulative event frequency reported for the study with the severity and frequency reported in the applicable reference document.

Reporting requirements will also be based on the investigator's assessment of causality and seriousness, with allowance for upgrading by the sponsor as needed.

6. STATISTICAL CONSIDERATIONS AND ANALYSIS PLAN

This open-label, multi-center study will concentrate on the analysis of safety and tolerability of the combination of RO5479599 with pertuzumab and paclitaxel for the dose cohorts investigated.

6.1 DETERMINATION OF SAMPLE SIZE

Approximately two cohorts, each including approximately 20 patients, are considered sufficient to estimate the incidence of DLTs and other AEs, taking into consideration the evidence of RO5479599 monotherapy and RO5479599 combination therapy with erlotinib and cetuximab in the ongoing phase I Study BP27771.

The cohort size of 20 patients is tailored to detect, with a specified confidence, the chance that the toxicity rate is below 20%. To evaluate the cohort size, Table 7 shows the results of simulations carried out with a Beta (0.2, 0.8) prior. The decision criterion used is the following: A dose level of RO5479599 in combination with pertuzumab and paclitaxel is defined as safe if, given the number of toxicity events observed in 20 patients, the posterior probability that the toxicity rate less or equal to 20% is at least 50%. This 50% threshold was chosen based on criteria that have been applied elsewhere in the literature in conjunction with proof-of-concept efficacy criteria [57, 58]. This is considered as sufficient confidence in the evaluation of the decision criteria for the purpose of monitoring the safety of each cohort. These considerations can be applied both for DLTs as well as other safety events of importance. A total of 10000 simulations were carried out for each scenario and the posterior probability was calculated via a Beta-Binomial Bayesian model of the toxicity events. Informative priors may also be investigated, based on historical data on the toxicity of pertuzumab and paclitaxel.

Table 7 Decision Criteria for Safety Based on Posterior Probabilities

True DLT/toxicity rate	Posterior probability (Toxicity rate ≤ 20%) ≥ 50%	Posterior probability (Toxicity rate ≤ 20%) < 50%
10%	96%	4%
15%	83%	17%
20%	64%	36%
30%	24%	76%

The table shows the chance that we would have to declare our dose safe/unsafe, given various underlying true toxicity profiles. In order to declare our dose safe, we need to have at least 50% confidence, given the data we observed, that the true toxicity rate is lower or equal to 20%. These true toxicity profiles can be considered either as true DLT rates or as the true rate of toxicity of a specific AE.

With respect to the dose escalation phase of 6 patients, Table 8 shows the probability of observing at least 1, 2 or 3 DLTs in a sample of 6 patients, given a true toxicity rate between 10% and 30%.

Table 8 Probability of Observing DLTs in the Dose escalation Phase

True DLT probability	Probability that DLT ≥ 1	Probability that DLT ≥ 2	Probability that DLT ≥ 3
10%	47%	11%	2%
15%	62%	22%	5%
20%	74%	34%	10%
25%	82%	47%	17%
30%	88%	58%	26%

A total of 46 patients will be enrolled in the study, should two dose cohorts be investigated, only one dose cohort be expanded to 20 patients and another expansion cohort added for patients with no prior chemotherapy for MBC and/or a maximum of only one prior chemotherapy regimen in adjuvant or neoadjuvant setting. Should toxicity warrant evaluation of additional patients in the other dose cohort, then the maximum sample size may go up to a total of 60 patients. **Should toxicity warrant evaluation of a third dose, then the maximum sample size may go up to a total of 66 patients.**

6.2 SUMMARIES OF CONDUCT OF STUDY

Protocol deviations will be evaluated and tabulated by cohort. Data on treatment administration will also be evaluated to determine deviations from the protocol.

6.3 ANALYSIS POPULATIONS

6.3.1 Safety Analysis Population

All patients who have received at least one dose of the study treatment, whether prematurely withdrawn from the study or not, will be included in the safety analysis.

6.3.2 Pharmacokinetic Analysis Population

Patients will be excluded from the PK analysis population if they significantly violate the inclusion or exclusion criteria, deviate significantly from the protocol or if data are unavailable or incomplete which may influence the PK analysis. Excluded cases will be documented together with the reason for exclusion. All decisions on exclusions from the analysis will be made prior to database closure.

6.3.3 Efficacy Analysis Population

All patients enrolled in the study and who receive at least one dose of study treatment will be included in the efficacy evaluation.

6.4 SUMMARIES OF TREATMENT GROUP COMPARABILITY

Patients will be listed and tabulated by cohort in terms of demographics, baseline characteristics, patient disposition, compliance with treatment visits and medical history/prior therapies. All descriptive statistics will be provided by cohort/dose level.

6.5 SAFETY ANALYSES

All safety analyses will be based on the safety analysis population.

Safety will be determined by AEs, laboratory tests, vital signs, electrocardiogram, echocardiogram, physical examinations and ECOG PS. Descriptive statistics will be used to summarize all safety data by dose cohort.

AE data will be reported in listings and presented in frequency tables by MedDRA terms. The severity of AEs will be graded according to the NCI CTC AE v4.0. Summaries of AE by grade, seriousness and relationship to study treatment will be presented, as well as

summaries of AEs leading to death, premature withdrawal from study treatment. DLTs will be tabulated for each dose's dose escalation phase.

For laboratory data, summary tables of change from baseline over time based on SI (Standard International) units will be displayed. Shifts in NCI-CTC AE grades v4.0 from baseline to the worst grade observed during treatment will be presented for selected laboratory parameters.

Descriptive statistics will be used to summarize ECOG PS. Vital signs, echocardiograms and ECGs will be reported in listings.

Exposure to study medication will be summarized by total duration of study medication, number of cycles started and cumulative dose using descriptive statistics. Dose modifications, interruptions and their reasons will be presented.

6.5.1 Adverse Events

The original terms recorded on the eCRF by the investigator for AEs will be standardized by the sponsor.

AEs will be summarized by mapped term and appropriate thesaurus level.

6.5.2 Clinical Laboratory Test Results

All clinical laboratory data will be stored on the database in the units in which they were reported. Patient listings and summary statistics at each assessment time will be presented using the International System of Units (SI units; Système International d'Unités). Laboratory data not reported in SI units will be converted to SI units before processing.

Laboratory test values will be presented by individual listings with flagging of values outside the normal ranges.

6.5.2.1 Standard Reference Ranges and Transformation of Data

Roche standard reference ranges, rather than the reference ranges of the investigator, will be used for all parameters. For most parameters, the measured laboratory test result will be assessed directly using the Roche standard reference range. Certain laboratory parameters will be transformed to Roche's standard reference ranges.

A transformation will be performed on certain laboratory tests that lack sufficiently common procedures and have a wide range of investigator ranges, e.g., enzyme tests that include AST, ALT, alkaline phosphatase and total bilirubin. Since the standard reference ranges for these parameters have a lower limit of zero, only the upper limits of the ranges will be used in transforming the data.

6.5.2.2 Definition of Laboratory Abnormalities

For all laboratory parameters included, there exists a Roche predefined standard reference range. Laboratory values falling outside this standard reference range will be labeled “H” for high or “L” for low in patient listings of laboratory data.

In addition to the standard reference range, a marked reference range has been predefined by Roche for each laboratory parameter. The marked reference range is broader than the standard reference range. Values falling outside the marked reference range that also represent a defined change from baseline will be considered marked laboratory abnormalities (i.e. potentially clinically relevant). If a baseline value is not available for a patient, the midpoint of the standard reference range will be used as the patient’s baseline value for the purposes of determining marked laboratory abnormalities. Marked laboratory abnormalities will be labeled in the patient listings as “HH” for very high or “LL” for very low.

6.5.3 Vital Signs

Vital signs data will be presented by individual listings with flagging of values outside the normal ranges and flagging of marked abnormalities. In addition, tabular summaries will be used, as appropriate.

6.5.4 ECG Data Analysis

ECG data will be presented by individual listings with flagging of values outside the normal ranges and flagging of marked abnormalities. In addition, tabular summaries will be used, as appropriate.

6.5.5 Echocardiogram Analysis

Echocardiogram data from the local study centers and the central laboratory will be presented by individual listings with flagging of values outside the normal ranges and flagging of marked abnormalities.

6.5.6 Concomitant Medications

The original terms recorded on the patients’ eCRF by the investigator for concomitant medications will be standardized by the sponsor by assigning preferred terms.

Concomitant medications will be presented in summary tables and listings.

6.6 EFFICACY ANALYSES

All patients enrolled in the study and who receive at least one dose of study treatment (RO5479599, pertuzumab or paclitaxel) will be included in the efficacy evaluation.

Tumor response data (including disease control and objective response) will be evaluated according to RECIST 1.1 criteria [59] and will be reported using percentages and accompanying 90% confidence intervals. Duration of response and PFS will be summarized using time to event analyses (including median time to event with 95%

confidence intervals) and Kaplan-Meier curves. Definitions and handling of censoring rules (where applicable) for DCR, ORR, duration of response and PFS are given below. OS may also be evaluated if data are mature enough and, if so, it will be summarized by Kaplan-Meier analysis.

Disease control rate (DCR): A patient is considered to have disease control if their best overall response is either CR or PR or SD. All other patients, including those patients for whom a post baseline tumor assessment was not performed, will be considered as 'non-responders' in this analysis of DCR.

Objective response rate (ORR): Objective response is defined as the occurrence of a confirmed CR or PR. An objective response needs to be confirmed at least 6 weeks after the initial response. Patients who do not meet this criterion, including patients for whom a post baseline tumor assessment was not performed, will be considered 'non-responders' in the analysis of ORR.

Duration of response (DOR): It is calculated only for patients with best overall response of CR/PR and is defined as time from the first confirmed documented objective response (CR/PR, whichever status is recorded first) to the date of first documented disease progression or death. Patients who have not progressed or died at the time of analysis will be censored at the date of their last evaluable tumor assessment.

Progression-free survival (PFS): It is defined as the time from the first dose of study medication to disease progression or death, whichever occurs first. Patients who do not progress or die while being followed will be censored on the date of the last valid tumor assessment. Patients without a post-baseline tumor assessment will conservatively be censored on the date of first study medication, i.e. PFS will be assigned a value of one day and will be censored in the analysis.

Overall survival (OS): It is defined as the time from the first dose of study medication to death. Patients who do not die while being followed up will be censored on the date of last contact.

Tumor growth in a medication-free period could be compared with tumor growth on treatment, if sufficient information on the pre-treatment assessments can be obtained.

6.6.1 Primary Efficacy Endpoint

As this is a Phase Ib design and therefore, all above endpoints will be evaluated, except OS, which will be evaluated only if sufficient/mature data will be available.

6.7 PHARMACODYNAMIC ANALYSES

All PD parameters will be presented by listings and descriptive summary statistics separately by group or cohorts.

PD parameters from whole blood and fresh tumor biopsies include (but are not limited to) immune effector cells (T lymphocytes, NK cells, macrophages etc.), cytokines and HER pathway markers (HER3, phosphorylated HER3, HER2, cMET etc.) and tumor-related

mutations (e.g., kRAS, HER4 etc.). These parameters will be analyzed using listings, descriptive summary statistics and graphical techniques by dose group and patient as appropriate. Absolute and percent change from baseline values will be calculated for each parameter where applicable.

6.8 PHARMACOKINETIC ANALYSES

PK parameters will be derived from serum concentrations and will be summarized using descriptive statistics.

Estimation of PK parameters may be performed using standard non-compartmental analysis (NCA) or compartmental analysis (CA). Actual sampling times will be used to calculate the final PK parameters. Serum concentrations that are below the limit of quantification for the assay (BLQ) will be replaced with zero for the calculation of PK parameters. Missing PK data points will not be replaced.

All serum concentrations and derived PK parameters will be presented in PK data listings for individual patients by dose cohort. Sort order of PK listings will be by dose cohort, patient number, cycle, study day and time point within study day.

PK parameters will be summarized using the following descriptive statistics: number of patients (n), mean, standard deviation (SD), median, minimum (min), maximum (max), and percent coefficient of variation (CV[%]). Graphical depictions of the mean and individual plasma concentration-time curves will be presented.

Exploratory analysis and graphical representations will be performed to assess the possible relationship between the PK of RO5479599 and selected biomarkers, efficacy or safety using NCA and CA approaches as appropriate.

Exploratory analysis will be performed to assess the PK of pertuzumab and paclitaxel in combination with RO5479599.

Non-linear mixed effects modeling (with software NONMEM [60] may also be used to analyze the serum concentration-time data of RO5479599 **and pertuzumab**. Population and individual PK parameters will be estimated and the influence of various covariates on these parameters will be investigated. Data from this study may be pooled with data from future studies.

Exploratory analyses may be conducted to investigate the relationship between RO5479599 exposure and efficacy and safety parameters. If possible, non-linear mixed effects modeling (with software NONMEM) may be used to investigate the relationship between RO5479599 exposure and efficacy and safety parameters.

Details of the mixed-effects modeling and the exploratory analyses will be described in a Modeling and Simulation Analysis Plan and the results of these analyses would be reported in a document separate from the clinical study report.

6.9 EXPLORATORY ANALYSES

Relationships between PD and PK data and efficacy variables will be evaluated in an exploratory manner. No inferential statistical tests will be implemented, given the small sample sizes with respect to any efficacy objective.

Relationships between percent change in PK parameters, PD markers and clinical response will be assessed as appropriate through data tabulations and graphical techniques. Sample size permitting, statistical measures of correlations may also be calculated.

6.10 INTERIM ANALYSES

No formal interim analyses are planned. Safety data, PK and, where available, PD data will be evaluated on a continuous basis and cohort recruitment may be stopped at any time. A safety review committee comprising of investigators and members of the Roche Study Management Team will review the data on an on-going basis. The study foresees no separate Safety Committee, as safety will be monitored on an on-going basis.

7. DATA COLLECTION AND MANAGEMENT

7.1 DATA QUALITY ASSURANCE

The overall procedures for quality assurance of clinical study data, including data collection and management, are described in the Roche Standard Operational Procedures.

Accurate and reliable data collection will be assured by verification and cross-check of the eCRF against the investigator's records by the study monitor (source document verification), and the maintenance of a study treatment-dispensing log by the investigator.

Data for this study will be recorded via an online Electronic Data Capture (EDC) system using eCRFs. The data will be transcribed by the study center from the paper source documents into the eCRF or will be loaded from electronic files (e.g. safety lab data). In no case is the eCRF to be considered as source data for this study.

A comprehensive validation check program will verify the data. Discrepancies will be generated automatically in the system at the point of entry or added manually for resolution by the investigator.

Throughout the study, the study management team will review data according to the Data Handling Manual.

7.2 ELECTRONIC CASE REPORT FORMS

Data for this study will be captured via an on line Electronic Data Capture (EDC) system. The data collected in the source documents is entered onto the study eCRF. An audit trail will maintain a record of initial entries and changes made; reasons for change; time and date of entry; and user name of person authorizing entry or change. For each enrolled, an eCRF must be completed and electronically signed by the principal

investigator or authorized delegate from the study staff. If a patient withdraws from the study, the reason must be noted on the eCRF. If a patient is withdrawn from the study because of a treatment‑limiting AE, thorough efforts should be made to clearly document the outcome.

The investigator should ensure the accuracy, completeness and timeliness of the data reported to the sponsor/CRO in the eCRFs and in all required reports.

eCRFs will be submitted electronically to the sponsor/CRO and should be handled in accordance with instructions from the sponsor/CRO.

At the end of the study, the investigator will receive patient data for his or her site in a readable format on a compact disc that must be kept with the study records. Acknowledgement of receipt of the compact disc is required.

7.3 ELECTRONIC PATIENT-REPORTED OUTCOME DATA

Not applicable.

7.4 SOURCE DATA DOCUMENTATION

Study monitors will perform ongoing source data verification to confirm that critical protocol data (i.e., source data) entered into the eCRFs by authorized site personnel are accurate, complete, and verifiable from source documents.

Source documents (paper or electronic) are those in which patient data are recorded and documented for the first time. They include, but are not limited to, hospital records, clinical and office charts, laboratory notes, memoranda, patient-reported outcomes, evaluation checklists, pharmacy dispensing records, recorded data from automated instruments, copies of transcriptions that are certified after verification as being accurate and complete, microfiche, photographic negatives, microfilm or magnetic media, X‑rays, patient files, and records kept at pharmacies, laboratories, and medico‑technical departments involved in a clinical trial.

Before study initiation, data to be entered directly into the eCRFs (i.e., no prior written or electronic record of the data) and considered source data must be defined in the Trial Monitoring Plan.

Source documents that are required to verify the validity and completeness of data entered into the eCRFs must not be obliterated or destroyed and must be retained per the policy for retention of records described in Section 7.6.

To facilitate source data verification, the investigators and institutions must provide the sponsor direct access to applicable source documents and reports for trial‑related monitoring, sponsor audits, and IRB/EC review. The investigational site must also allow inspection by applicable health authorities.

7.5 USE OF COMPUTERIZED SYSTEMS

When clinical observations are entered directly into an investigational site's computerized medical record system (i.e., in lieu of original hardcopy records), the electronic record can serve as the source document if the system has been validated in accordance with health authority requirements pertaining to computerized systems used in clinical research. An acceptable computerized data collection system allows preservation of the original entry of data. If original data are modified, the system should maintain a viewable audit trail that shows the original data as well as the reason for the change, name of the person making the change, and date of the change.

7.6 RETENTION OF RECORDS

Records and documents pertaining to the conduct of this study and the distribution of IMP, including eCRFs, ePRO data (if applicable), Informed Consent Forms, laboratory test results, and medication inventory records, must be retained by the Principal Investigator for at least 15 years after completion or discontinuation of the study, or for the length of time required by relevant national or local health authorities, whichever is longer. After that period of time, the documents may be destroyed, patient to local regulations. No records may be disposed of without the written approval of the sponsor. Written notification should be provided to the sponsor prior to transferring any records to another party or moving them to another location.

8. ETHICAL CONSIDERATIONS

8.1 COMPLIANCE WITH LAWS AND REGULATIONS

This study will be conducted in full conformance with the ICH E6 guideline for Good Clinical Practice and the principles of the Declaration of Helsinki, or the laws and regulations of the country in which the research is conducted, whichever affords the greater protection to the individual. The study will comply with the requirements of the ICH E2A guideline (Clinical Safety Data Management: Definitions and Standards for Expedited Reporting) see Appendix 6. Studies conducted in the United States or under a U.S. Investigational New Drug (IND) application will comply with U.S. FDA regulations and applicable local, state, and federal laws. Studies conducted in the EU/EEA will comply with the EU Clinical Trial Directive (2001/20/EC).

8.2 INFORMED CONSENT

The sponsor's sample Informed Consent Form (and ancillary sample Informed Consent Forms such as a Child's Assent or Caregiver's Informed Consent Form, if applicable) will be provided to each site. If applicable, it will be provided in a certified translation of the local language. The sponsor or its designee must review and approve any proposed deviations from the sponsor's sample Informed Consent Forms or any alternate consent forms proposed by the site (collectively, the "Consent Forms") before IRB/EC submission. The final IRB/EC‑approved Consent Forms must be provided to the sponsor for health authority submission purposes according to local requirements.

The Consent Forms must be signed and dated by the patient or the patient's legally authorized representative before his or her participation in the study. The case history or clinical records for each patient shall document the informed consent process and that written informed consent was obtained prior to participation in the study.

The Consent Forms should be revised whenever there are changes to study procedures or when new information becomes available that may affect the willingness of the patient to participate. The final revised IRB/EC-approved Consent Forms must be provided to the sponsor for health authority submission purposes.

Patients must be re-consented to the most current version of the Consent Forms (or to a significant new information/findings addendum in accordance with applicable laws and IRB/EC policy) during their participation in the study. For any updated or revised Consent Forms, the case history or clinical records for each patient shall document the informed consent process and that written informed consent was obtained using the updated/revised Consent Forms for continued participation in the study.

A copy of each signed Consent Form must be provided to the patient or the patient's legally authorized representative. All signed and dated Consent Forms must remain in each patient's study file or in the site file and must be available for verification by study monitors at any time.

8.3 INSTITUTIONAL REVIEW BOARD OR ETHICS COMMITTEE

This protocol, the Informed Consent Forms, any information to be given to the patient, and relevant supporting information must be submitted to the IRB/EC by the Principal Investigator and reviewed and approved by the IRB/EC before the study is initiated. In addition, any patient recruitment materials must be approved by the IRB/EC.

The Principal Investigator is responsible for providing written summaries of the status of the study to the IRB/EC annually or more frequently in accordance with the requirements, policies, and procedures established by the IRB/EC. Investigators are also responsible for promptly informing the IRB/EC of any protocol amendments (see Section 9.4).

In addition to the requirements for reporting all AEs to the sponsor, investigators must comply with requirements for reporting SAEs to the local health authority and IRB/EC. Investigators may receive written IND safety reports or other safety‑related communications from the sponsor. Investigators are responsible for ensuring that such reports are reviewed and processed in accordance with health authority requirements and the policies and procedures established by their IRB/EC, and archived in the site's study file.

8.4 CONFIDENTIALITY

The sponsor maintains confidentiality standards by coding each patient enrolled in the study through assignment of a unique patient identification number. This means that patient names are not included in data sets that are transmitted to any sponsor location.

Patient medical information obtained by this study is confidential and may only be disclosed to third parties as permitted by the Informed Consent Form (or separate authorization for use and disclosure of personal health information) signed by the patient, unless permitted or required by law.

Medical information may be given to a patient's personal physician or other appropriate medical personnel responsible for the patient's welfare, for treatment purposes.

Data generated by this study must be available for inspection upon request by representatives of the U.S. FDA and other national and local health authorities, sponsor monitors, representatives, and collaborators, and the IRB/EC for each study site, as appropriate.

8.5 FINANCIAL DISCLOSURE

Investigators will provide the sponsor with sufficient, accurate financial information in accordance with local regulations to allow the sponsor to submit complete and accurate financial certification or disclosure statements to the appropriate health authorities. Investigators are responsible for providing information on financial interests during the course of the study and for one year after completion of the study (i.e. LPLV).

9. STUDY DOCUMENTATION, MONITORING, AND ADMINISTRATION

9.1 STUDY DOCUMENTATION

The investigator must maintain adequate and accurate records to enable the conduct of the study to be fully documented, including but not limited to the protocol, protocol amendments, Informed Consent Forms, and documentation of IRB/EC and governmental approval. In addition, at the end of the study, the investigator will receive the patient data, which includes an audit trail containing a complete record of all changes to data.

Roche shall also submit an Annual Safety Report once a year to the IEC and CAs according to local regulatory requirements and timelines of each country participating in the study.

Sampling for the RCR is contingent on review and approval for the exploratory biomarker assessments and written informed consent by an appropriate regulatory body (depending on the country where the study is performed) and a site's Institutional Review Board (IRB) / Ethics Committee (EC). If a regulatory or site's IRB/ EC does not approve the sampling for the exploratory assessments the section on biomarker sampling will not be applicable.

9.2 SITE INSPECTIONS

Site visits will be conducted by the sponsor or an authorized representative for inspection of study data, patients' medical records, and eCRFs. The investigator will permit national and local health authorities, sponsor monitors, representatives, and collaborators, and the IRBs/ECs to inspect facilities and records relevant to this study.

9.3 PUBLICATION OF DATA AND PROTECTION OF TRADE SECRETS

The results of this study may be published or presented at scientific meetings. If this is foreseen, the investigator agrees to submit all manuscripts or abstracts to the sponsor prior to submission. This allows the sponsor to protect proprietary information and to provide comments based on information from other studies that may not yet be available to the investigator.

The sponsor will comply with the requirements for publication of study results. In accordance with standard editorial and ethical practice, the sponsor will generally support publication of multicenter trials only in their entirety and not as individual center data. In this case, a coordinating investigator will be designated by mutual agreement.

Any formal publication of the study in which contribution of sponsor personnel exceeded that of conventional monitoring will be considered as a joint publication by the investigator and the appropriate sponsor personnel.

Authorship will be determined by mutual agreement and in line with International Committee of Medical Journal Editors authorship requirements.

Any inventions and resulting patents, improvements, and/or know-how originating from the use of data from this study will become and remain the exclusive and unburdened property of the sponsor, except where agreed otherwise.

9.4 PROTOCOL AMENDMENTS

Any protocol amendments will be prepared by the sponsor. Protocol amendments will be submitted to the IRB/EC and to regulatory authorities in accordance with local regulatory requirements.

Approval must be obtained from the IRB/EC and regulatory authorities (as locally required) before implementation of any changes, except for changes necessary to eliminate an immediate hazard to patients or changes that involve logistical or administrative aspects only (e.g., change in Medical Monitor or contact information).

10. REFERENCES

1. Yarden Y, Sliwskowski MX, Untangling the ErbB signaling network. Nature Rev Mol Cell Biol. 2001; 2 :127-37
2. Van Cutsem E, Köhne CH, Hitre E, et al. Cetuximab and chemotherapy as initial treatment for metastatic colorectal cancer. N Engl J Med. 2009 Apr 2; 360(14):1408-17.
3. Vermorken JB, Mesia R, Rivera F, et al. Platinum-based chemotherapy plus cetuximab in head and neck cancer. N Engl J Med. 2008 Sep 11; 359(11):1116-27.
4. Moore MJ, Goldstein D, Hamm J, et al. Erlotinib plus gemcitabine compared with gemcitabine alone in patients with advanced pancreatic cancer: a phase III trial of the National Cancer Institute of Canada Clinical Trials Group. J Clin Oncol. 2007;25:1960-1966
5. Shepherd FA, Rodrigues Pereira J, Ciuleanu T, et al. Erlotinib in previously treated non-small-cell lung cancer. N Engl J Med. 2005 Jul 14; 353(2):123-32.
6. Rosell, R. Gervais, A. Vergnenegre, B. et al. Erlotinib versus chemotherapy (CT) in advanced non-small cell lung cancer (NSCLC) patients (p) with epidermal growth factor receptor (EGFR) mutations: Interim results of the European Erlotinib Versus Chemotherapy (EURTAC) phase III randomized trial. J Clin Oncol 29: 2011 (suppl; abstr 7503)
7. Smith I, Procter M, Gelber RD, et al. 2-year follow-up of trastuzumab after adjuvant chemotherapy in HER2-positive breast cancer: a randomised controlled trial. Lancet. 2007 Jan 6;369(9555):29-36.
8. Blackwell KL, Miles D, Gianni L, et al. Primary results from EMILIA, a phase III study of trastuzumab emtansine (T-DM1) versus capecitabine (X) and lapatinib (L) in HER2-positive locally advanced or metastatic breast cancer (MBC) previously treated with trastuzumab (T) and a taxane. J Clin Oncol 30, 2012 (suppl; abstr LBA1)
9. Baselga J, Cortes J, Kim SB, et al.Pertuzumab plus Trastuzumab plus Docetaxel for Metastatic Breast Cancer. N Engl J Med 2012; 366:109–19
10. Slamon DJ, Leyland-Jones, B, Shak S, et al. Use of chemotherapy plus a monoclonal antibody against HER2 for metastatic breast cancer that overexpresses HER2. N Engl J Med 2001, 344(11):783-792.
11. Jones RB, Gordus A, Krall JA, et al. A quantitative protein interaction network for the ErdB receptors using protein microarrays. Nature 2006; 439:168−74.
12. Xia W, Lau YK, Zhang HZ, et al. Combination of EGFR, HER-2/neu, and HER-3 is a stronger predictor for the outcome of oral squamous cell carcinoma than any individual family members. Clin Cancer Res. 1999 Dec; 5(12):4164-74.

13. Robinson AG, Turbin D, Thomson T, et al. Molecular predictive factors in patients receiving trastuzumab-based chemotherapy for metastatic disease. Clin Breast Cancer. 2006 Aug; 7(3):254-61.

14. Chiu CG, Masoudi H, Leung S, et al. HER-3 overexpression is prognostic of reduced breast cancer survival: a study of 4046 patients. Ann Surg. 2010 Jun; 251(6):1107-16.

15. Sergina NV, Rausch M, Wang D, et al. Escape from HER-family tyrosine kinase inhibitor therapy by the kinase-inactive HER3. Nature. 2007 Jan 25; 445(7126):437-41.

16. Engelman JA, Zejnullahu K, Mitsudomi T, et al. MET amplification leads to gefitinib resistance in lung cancer by activating ERBB3 signaling. Science. 2007 May 18; 316(5827):1039-43.

17. Wheeler DL, Huang S, Kruser TJ, et al. Mechanisms of acquired resistance to cetuximab: role of HER (ErbB) family members. Oncogene. 2008 Jun 26; 27(28):3944-56.

18. Sithanandam G, Anderson LM. The ERBB3 receptor in cancer and cancer gene therapy. Cancer Gene Ther. 2008 Jul; 15(7):413-48.

19. Arteaga CL. HER3 and mutant EGFR meet MET. Nat Med. 2007 Jun; 13(6):675-7.

20. Cartron G, Dacheux L, Salles G, et al. Therapeutic activity of humanized anti-CD20 monoclonal antibody and polymorphism in IgG Fc receptor FcgammaRIIIa gene. Blood. 2002 Feb 1; 99(3):754-8.

21. Musolino A et al. Immunoglobulin G fragment C receptor polymorphisms and response to trastuzumab-based treatment in patients with HER-2/neu-positive metastatic breast cancer [abstract]. In: American Association for Cancer Research Annual Meeting: Proceedings; 2007 Apr 14-18; Los Angeles, CA. Philadelphia (PA): AACR; 2007. Abstract nr 4188

22. Sibéril S, Dutertre CA, Fridman WH, et al. Fcgamma R: The key to optimize therapeutic antibodies? Crit Rev Oncol Hematol. 2007 Apr; 62(1):26-33.

23. Arnould L, Gelly M, Penault-Llorca F, et al. Trastuzumab-based treatment of HER2-positive breast cancer: an antibody-dependent cellular cytotoxicity mechanism? Br J Cancer. 2006 Jan 30; 94(2):259-67.

24. Cho HS, Mason K, Ramyar KX, et al. Structure of the extracellular region of HER2 alone and in complex with the Herceptin Fab. Nature 2003; 421:756-60.

25. Franklin MC, Carey KD, Vajdos FF, et al. Insights into ErbB signaling from the structure of the ErbB2-pertuzumab complex. Cancer Cell 2004; 5(4):317−28.

26. Baselga J, Gelmon KA, Verma S, et al. Phase II trial of pertuzumab and trastuzumab in patients with human epidermal growth factor receptor 2-positive metastatic breast cancer that progressed during prior trastuzumab therapy. J Clin Oncol. 2010 Mar 1;28(7):1138-44.

27. Baselga J. Treatment of HER2-overexpressing breast cancer. Ann Oncol 2010; Suppl 7:vii36-40.

28. Cortés J, Fumoleau P, Bianchi GV, et al. Pertuzumab monotherapy after trastuzumab-based treatment and subsequent reintroduction of trastuzumab: activity and tolerability in patients with advanced human epidermal growth factor receptor 2-positive breast cancer. J Clin Oncol. 2012 May 10;30(14):1594-600.

29. Gianni L, Pienkowski T, Im YH, et al. Efficacy and safety of neoadjuvant pertuzumab and trastuzumab in women with locally advanced, inflammatory, or early HER2-positive breast cancer (NeoSphere): a randomised multicentre, open-label, phase 2 trial. Lancet Oncol. 2012 Jan;13(1):25-32.

30. Baselga J, Cameron D, Miles D, et al. Objective response rate in a Phase II multicenter trial of pertuzumab (P), a HER2 dimerization inhibiting monoclonal antibody, in combination with trastuzumab (T) in patients (pts) with HER2-positive metastatic breast cancer (MBC) which has progressed during treatment with T. J Clin Oncol 2007; ASCO Annual Meeting Proceedings; 25:18S (June 20 Supplement): 1004.

31. Verweij J, Clavel M, Chevalier B. Paclitaxel (Taxol) and docetaxel (Taxotere): not simply two of a kind. Annals of Oncology 1994;5:495–505.

32. ten Tije AJ, Verweij J, Loos WJ, Sparreboom A. Pharmacological effects of formulation vehicles: implications for cancer chemotherapy. Clinical Pharmacokinetics 2003;42:665–85.

33. Nabholtz JM, Gelmon K, Bontenbal M, et al. Multicenter, randomized comparative study of two doses of paclitaxel in patients with metastatic breast cancer. Journal of Clinical Oncology 1996;14:1858–67.

34. Green MC, Buzdar AU, Smith T, et al. Weekly paclitaxel improves pathologic complete remission in operable breast cancer when compared with paclitaxel once every 3 weeks. J Clin Oncol 2005;23:5983–92.

35. Sparano JA, Wang M, Martino S, et al. Weekly paclitaxel in the adjuvant treatment of breast cancer. The New England Journal of Medicine 2008;358:1663–71.

36. Fountzilas G, Dafni U, Dimopoulos MA, et al. A randomized phase III study comparing three anthracycline-free taxane-based regimens, as first line chemotherapy, in metastatic breast cancer: a Hellenic Cooperative Oncology Group study. Breast Cancer Research and Treatment 2009;115:87–99.

37. Seidman AD, Berry D, Cirrincione C, et al. Randomized phase III trial of weekly compared with every-3-weeks paclitaxel for metastatic breast cancer, with trastuzumab for all HER-2 overexpressors and random assignment to trastuzumab or not in HER-2 nonoverexpressors: final results of Cancer and Leukemia Group B protocol 9840. Journal of Clinical Oncology 2008;26:1642–9.

38. Verrill MW, Lee J, Cameron DA, et al. Anglo-Celtic IV: first results of a UK National Cancer Research Network randomised phase 3 phar- macogenetic trial of weekly versus 3 weekly paclitaxel in patients with locally advanced or metastatic breast cancer (ABC). Journal of Clinical Oncology 2007;25 [abstract LBA1005].

39. Beslija S, Bonneterre J, Burstein HJ, et al. Third consensus on medical treatment of metastatic breast cancer. Annals of Oncology 2009;20:1771–85.

40. Cardoso F, Fallowfield L, Costa A, Castiglione M, Senkus E. Locally recurrent or metastatic breast cancer: ESMO Clinical Practice Guidelines for diagnosis, treatment and follow-up. Annals of Oncology 2011;22(Suppl. 6):vi25–30.

41. NCCN clinical practice guidelines in oncology: breast cancer version 1.2013. Available at: http://www.nccn.org/ professionals/physician_gls/pdf/breast.pdf [accessed 12.02.2013].

42. Miller K, Wang M, Gralow J, et al. Paclitaxel plus Bevacizumab versus Paclitaxel Alone for Metastatic Breast Cancer. N Engl J Med. 2007 Dec 27;357(26):2666-76.

43. Miles D, Chan A, Dirix LY, et al. Phase III study of bevacizumab plus docetaxel compared with placebo plus docetaxel for the first-line treatment of human epidermal growth factor receptor 2-negative metastatic breast cancer. JCO 2010 Jul 10;28(20):3239-47.

44. Gianni L, Lladó A, Bianchi G, et al. Open-label, phase II, multicenter, randomized study of the efficacy and safety of two dose levels of Pertuzumab, a human epidermal growth factor receptor 2 dimerization inhibitor, in patients with human epidermal growth factor receptor 2-negative metastatic breast cancer. J Clin Oncol. 2010 Mar 1;28(7):1131-7.

45. Hallek M, Fischer K, Fingerle-Rowson G, et al; International Group of Investigators; German Chronic Lymphocytic Leukaemia Study Group. Addition of rituximab to fludarabine and cyclophosphamide in patients with chronic lymphocytic leukaemia: a randomised, open-label, phase 3 trial. . Lancet. 2010 Oct 2;376(9747):1164-74

46. Liu J, Patel R, Kato G, et al. A Phase 1 Study Of The Anti-ERBB3 Antibody MM-121 In Combination With Weekly Paclitaxel In Patients With Advanced Gynecological And Breast Cancers ESMO 2012, Abstract #974PD

47. Richards D, Braiteh F, Anthony S,et al. A Phase 1 Study Of MM-111; A Bispecific HER2/HER3 Antibody Fusion Protein, Combined With Multiple Treatment Regimens In Patients With Advanced HER2 Positive Solid Tumors. ESMO 2012 Abstract #496P

48. Kaye SB, Poole CJ, Dańska-Bidzińska A, et al. A randomized phase II study evaluating the combination of carboplatin-based chemotherapy with pertuzumab versus carboplatin-based therapy alone in patients with relapsed, platinum-sensitive ovarian cancer. Ann. Oncol 2013 Jan;24(1):145-52.

49. Makhija S, Amler LC, Glenn D, et al. Clinical activity of gemcitabine plus pertuzumab in platinum-resistant ovarian cancer, fallopian tube cancer, or primary peritoneal cancer. J Clin Oncol. 2010 Mar 1;28(7):1215-23.

50. Schneeweiss A, Chia S, Hickish T, et al. Neoadjuvant Pertuzumab and Trastuzumab Concurrent or Sequential with an Anthracycline-Containing or Concurrent with an Anthracycline-Free Standard Regimen: A Randomized Phase II Study (TRYPHAENA). SABCS 2011, Abstract #S5-6

51. Albanell J, Montagut C, Jones ET, et al. A phase I study of the safety and pharmacokinetics of the combination of pertuzumab (rhuMab 2C4) and capecitabine in patients with advanced solid tumors. Clin. Cancer Res. 2008 May 1;14(9):2726-31.

52. Attard G, Kitzen J, Blagden SP, et al. A phase Ib study of pertuzumab, a recombinant humanised antibody to HER2, and docetaxel in patients with advanced solid tumours. Br J Cancer. 2007 Nov 19;97(10):1338-43

53. Morschauser F, Marlton P, Vitolo U, et al. Results of a phase I/II study of ocrelizumab, a fully humanized anti-CD20 mAb, in patients with relapsed/refractory follicular lymphoma. Ann Oncol. 2010 Sep;21(9):1870-6.

54. Bean J, Brennan C, Shih JY, et al. (2007) METamplification occurs with or without T790M mutations in EGFR mutant lung tumors with acquired resistance to gefitinib or erlotinib. Proc Natl Acad Sci U S A 104: 20932 20937.

55. Corso S, Ghiso E, Cepero V, et al. Activation of HER family members in gastric carcinoma cells mediates resistance to MET inhibition. Mol Cancer. 2010 May 26;9:121.

56. Cancer Genome Atlas Network. Comprehensive molecular portraits of human breast tumours. Nature. 2012 Oct 4;490(7418):61-70.

57. Neuenschwander B, Rouyrre N, Hollaender N, et al. A proof of concept phase II non-inferiority criterion. Statist. Med. 2011, 30: 1618-1627

58. Di Scala L, Kerman J, and Neuenschwander B. Collection, synthesis, and interpretation of evidence: a proof-of-concept study in COPD. Statist. Med. 2013 (Article first published online: 10 JAN 2013)

59. Eisenhauer EA, Therasse P, Bogaerts J, et al. New response evaluation criteria in solid tumors: Revised RECIST guideline (version 1.1). Eur J Cancer 2009;45:228-47 (http://www.eortc.be/recist/documents/RECISTGuidelines.pdf)

60. Beal SL, Boeckman AJ and Sheiner LB. NONMEM user's guide, parts I-VIIII. San Francisco: Division of Clinical Pharmacology - University of California, 1992.

61. Le Tourneau C, Servois V, Dieras V, et al. Tumor growth kinetics assessment : added value to RECIST in cancer patients treated with molecularly targeted agents. Brit J Cancer 2012;106:854-857

Appendix 1 Schedule of Assessments

	Screening [s]	Treatment cycle (1 cycle = 21 days)											Subsequent cycles			Fup visit/early-discontinuation visit
Cycle		1				2			3				≥ 4			
Day	-28 to -1 days	1	2	8	15	1	8	15	1	8	15	21	1	8	15	28 (+/-3) days [m] post infusion
Informed consent & medical history	X															
Inclusion/exclusion criteria [r]	X															
Demographics	X															
Physical exam [l]	X[a]	X				X			X				X			X
Vital signs [b]	X		X			X			X				X			X
ECOG PS	X[a]	X				X			X				X			X
Pregnancy test [g]	X															X
Triplicate 12-lead ECG [i]	X		X						X			X				X
Echocardiogram [v]	X											X				X
Tumor biopsy [p]	X															
Primary archival tumor sample (if available) [t]	X															
Tumor assessment [h]	X											X				X
Latest CT scan (optional) [w]	X															
Tumor markers CEA & CA15-3 [f]	X											X				
Hematology [d]	X[a]	X	X			X			X				X			X
Biochemistry [e]	X[a]	X	X	X	X	X			X				X			X
Urinalysis [x]	X															X
Coagulation [c]	X[a]	X	X			X			X				X			
Leukocytes immunophenotyping [k]		X	X			X			X							
NK lymphocyte functional assessment [j]		X	X			X			X							
IgE and tryptase [o]						X			X				X			
Clinical genotyping [n]	X															
RO5479599 administration [u]			X[u]			X			X				X			
Pertuzumab administration [u]		X[u]				X			X				X			
Paclitaxel administration [u]		X[u]		X	X	X	X	X	X	X	X		X	X	X	
PK & HAHA samples [q]	Please see Appendix 2 for PK and HAHA timepoints															
AEs/Conmeds (as per Section 4.5)	←Continuously monitored→															

Appendix 1 Schedule of Assessments (cont.)

a) If screening assessments are performed within 36 h of the first drug administration, the assessments do not have to be repeated on Day 1 of Cycle 1. Otherwise hematology, biochemistry, coagulation, physical exam, and ECOG PS should be performed prior to first dose. Vital signs should always be performed prior to the first dose.

b) Vital signs include body temperature, systolic and diastolic blood pressure and pulse rate. During infusion of RO5479599, vital signs (including, if possible, supine diastolic and systolic blood pressure, pulse rate and temperature) have to be monitored pre-infusion, every 15 min until the end of infusion, and thereafter, every 30 min until the infusion line is removed. After completion of Cycle 5 vital signs have to be monitored only pre and post infusion if RO5479599 infusion has been tolerated well in previous cycles. Only screening, pre-dose, Fup visit/early discontinuation visit and any considered abnormalities for vital signs (as recorded as an unscheduled assessment) will be recorded in the eCRF.

c) Coagulation will be performed at screening, within 24 h pre infusion on Day 1 of each treatment cycle, on Day 2 of Cycle 1 pre infusion of RO5479599 and at 28 (+/-3) day Fup visit/early discontinuation visit including PT/INR and PTT. An additional sample will be taken in case an IRR occurs or in case of suspected disseminated intravascular coagulation to assess additional coagulation parameters, i.e. antithrombin III, fibrinogen, prothrombin time, fibrin degradation products, D-dimer according to clinical judgment.

d) Hematology should be performed at screening, within 24 h pre infusion on Day 1 of each cycle, on Day 2 of Cycle 1 pre infusion of RO5479599 and at 28 (+/-3) day Fup visit/early discontinuation visit. Hematology includes RBC, HGB, HCT, platelets, leucocytes, WBC differential including neutrophils, lymphocytes, monocytes, eosinophils and basophils.

e) Biochemistry should be performed at screening, within 24 h pre infusion on Day 1 of each cycle, on Day 2 of Cycle 1 pre infusion of RO5479599, on Day 8 and 15 of Cycle 1 pre infusion of paclitaxel and at 28 (+/-3) day Fup visit/early discontinuation visit. Biochemistry includes glucose, creatinine and creatinine clearance (by Cockcroft Gault formula [see Appendix 4]), AST, ALT, GGT, LDH, alkaline phosphatase, bilirubin, total protein, albumin, calcium (corrected for albumin level), and electrolytes (sodium, potassium, magnesium, chloride, phosphate). **In case of ≥Grade 2 diarrhea and/or in patients with any electrolyte imbalance an additional weekly blood sample shall be taken to monitor blood electrolyte levels. Blood electrolyte analysis should include at least sodium, chloride, calcium, phosphate, potassium, and magnesium.**

f) Tumor markers CEA and CA15-3 will be performed during screening and at the tumor assessments (every 9 weeks).

g) Serum pregnancy tests should be performed within 7 days prior 1st dosing and at 28 (+/-3) day Fup/early discontinuation visit.

h) Screening RECIST 1.1 tumor assessment can be performed up to 14 days prior to the first study treatment administration. While on study, RECIST 1.1 tumor assessments should be performed every 9 weeks ± 7 days and at the 28 (+/-3) day Fup visit/early discontinuation visit. Patients with previously documented tumor progression will not require additional tumor reassessment at the Fup visit/early discontinuation visit. CT or MRI imaging will form part of the tumor assessment and the same method should be used throughout the study.

Appendix 1 Schedule of Assessments (cont.)

i) Triplicate ECGs will be recorded at screening, prior to infusion and at the end of the infusion (+30 min) on Day 2 of Cycle 1 and on Day 1 of Cycle 3 and every 9 weeks (e.g. Cycle 3 Day 21) and at 28 (+/-3) day Fup visit/early discontinuation visit in all patients. Screening 12-lead single ECG must be performed within 7 days before first dose of study treatment. If able to, patients should be in a supine position for 5 min prior to the ECG. Rate, rhythm, interval durations, interval appearances and axis will be noted for each ECG. The following parameters will be obtained and recorded in the eCRF: PQ (PR), QRS, QT and heart rate. RR and QTcF will be calculated internally by Roche. T- and U-waves will be assessed by the investigator over all ECGs recorded at a given time point. T-waves should be captured as normal or abnormal and U-waves should be captured as absent/normal or abnormal. Recording must be done prior to PK sampling.

j) NK functional test should be performed C1D1: pre infusion; C1D2 pre infusion and 4h and 24 h post infusion of RO5479599; C2 and C3 pre-infusion. An additional blood sample will be taken at the time of relapse. **NK functional test will not be done for Extension Phase 2 Cohort patients.**

k) Leukocyte immunophenotyping should be performed C1D1: pre infusion; C1D2 pre infusion and 4h and 24 h post infusion of RO5479599; C2 and C3 pre-infusion. An additional blood sample will be taken at the time of relapse.

l) Physical exam should include height and weight at screening and weight only at subsequent visits including the Fup visit/early discontinuation visit.

m) The 28-day Fup/early discontinuation visit should be completed 28 days (± 3 days) post last dose of RO5479599. In case of early discontinuation, the visit can be brought forward to an earlier time point at the discretion of the investigator.

n) 3 ml blood sample will be collected to assess FcγRIII-158 polymorphism prior to first dose of study treatment.

o) For the assessment of IgE and tryptase a precautionary base line blood sample will be taken pre dose at each cycle from Cycle 2 onwards. For patients who experience an IRR ≥ grade 2 for the first time with the 2nd or subsequent RO5479599 infusion tryptase and IgE will be analyzed from an additional plasma sample.

p) A fresh (pretreatment) base line tumor biopsy (core needle biopsy [for superficial lesions a punch biopsy can be performed instead if an adequately deep specimen can be warranted and including a representative amount of tumor cells. Therefore, the punch biopsy should have a diameter of ≥ 2 mm at least.]) from primary tumor or metastases for patient inclusion based on HER2 and HER3 expression will be taken during the screening phase. Biopsies should be performed according to institutional guidelines, e.g. ensuring adequate coagulation status and platelet count. **A second, optional tumor biopsy may be taken at disease progression/relapse at the discretion of the investigator.**

q) HAHA and PK samples: For HAHA and PK sampling time points please refer to Appendix 2.

r) To be completed during screening, including ESF.

Appendix 1 Schedule of Assessments (cont.)

s) Screening will be up to 28 days from signed informed consent until 1st administration of study treatment.

t) Submission of primary archival tumor block is not a mandatory requirement but highly encouraged if available. To protect against oxidation and to allow IHC analysis in large batches, archived tumor block is preferred.

u) Study treatments will be administered in the consecutive sequence of paclitaxel, pertuzumab and RO5479599 on Day 1 of each treatment cycle except for Cycle 1. For Cycle 1, it is recommended to administer paclitaxel and pertuzumab on Day 1 and RO5479599 on Day 2. Pertuzumab will be administered at a loading dose of 840 mg during Cycle 1 followed by 420 mg for the remaining cycles in a q3w schedule. **Extension Phase Cohort 2 will commence at a reduced dose of pertuzumab, i.e. 420 mg for Cycle 1 followed by 420 mg for subsequent cycles.** Paclitaxel will be administered at 80 mg/m^2 in a qw schedule.

v) Echocardiography to assess LVEF will be performed during screening, then every 9 weeks and at 28 (+/-3) day Fup visit/early discontinuation visit in all patients.

w) Optionally, the latest pre-study CT scan should be provided for assessment of tumor growth kinetics within 6 weeks of the patient enrolling into the study.

x) Urinalysis will be performed at screening and at 28 (+/-3) day Fup visit/early discontinuation visit. Additional tests may be performed at the discretion of the investigator. It includes: glucose, protein, hemoglobin (blood), bilirubin, ketones, pH. If there is a clinically significant positive result (i.e. confirmed by a positive repeated sample), urine will be sent to the laboratory for microscopy and culture. If there is an explanation for the positive dipstick result, e.g. menses, it should be recorded, and there is no need to perform laboratory for microscopy and culture.

Appendix 2 Specific Schedule of Assessments for PK and HAHA

Schedule for dose escalation and Extension Phase Cohort 1 patients:

a Assessments on Cycle 1, End-of-study Assessments and Additional Assessments

Assessments	Day 1[g]			Day 2	Day 4	Day 8	Day 12	Day 14	Day 19	Day 21	Day 28 (+/-3 days) post-infusion Fup visit/early-discontinuation visit	Day 42-45 post-infusion Fup visit[b]	IRR	Disease progression	DLT
			Hours post-dose												
	Pre-dose	Post-infusion	3[d]	24[f]	72[a]	168[a]	264[a]	312[a]	432[a]	480[e]					
RO5479599 PK samples	X	X	X	X	X	X	X	X	X	X	X	X	X	X	X
HAHA	X[c]										X	X	X		

a) Timing of sampling within +/-24 h (please record).
b) Only for patients who are not entering another clinical trial or receiving further anti-tumor treatment.
c) HAHA sampling on all cycles prior IV infusion. An additional sample for HAHA assessment as part of an unscheduled assessment will be taken from patients with clinical signs of immune complex-mediated toxicities.
d) Timing of sampling +3 h.
e) Timing of sampling -24 h only (please record).
f) Timing of sampling within +24 h (please record).
g) Please note: Cycle 1 Day 1 for PK sampling is Day 2 in the Schedule of Assessments (Appendix 1).

Appendix 2 Specific Schedule of Assessments for PK and HAHA (cont.)

b Assessments on Cycle 4 and Cycle 8

Assessments	Day 1			Day 2	Day 4	Day 8	Day 12	Day 14	Day 19	Day 21
	Pre-dose	Post-infusion	Hours post-dose							
			3[b]	24[d]	72[a]	168[a]	264[a]	312[a]	432[a]	480[c]
RO5479599 PK samples	X	X	X	X	X	X	X	X	X	X
HAHA	X									

a) Timing of sampling within +/-24 h (please record).
b) Timing of sampling +3 h.
c) Timing of sampling -24 h only (please record).
d) Timing of sampling within +24 h (please record)

c Assessments for All Other Cycles

Assessments	Day 1	
	Pre-dose	Post-infusion
RO5479599 PK samples	X	X
HAHA	X	

Appendix 2 Specific Schedule of Assessments for PK and HAHA (cont.)

Schedule for Extension Phase Cohort 2 patients:

a RO5479599 Assessments on Cycle 1, End-of-study Assessments and Additional Assessments

Assessments	Day 1[f]			Day 2	Day 4	Day 8	Day 12	Day 14	Day 19	Day 28 (+/-3 days) post-infusion Fup visit/early-discontinuation visit	Day 42-45 post-infusion Fup visit[b]	IRR	Disease progression	DLT	≥Grade 2 diarrhea [g]
	Pre-dose	Post-infusion	Hours post-dose												
			3[d]	24[e]	72[a]	168[a]	264[a]	312[a]	432[a]						
RO5479599 PK samples	X	X	X	X	X	X	X	X	X	X	X	X	X	X	**X**
RO5479599 HAHA	X[c]									X	X	X			

a) Timing of sampling within +/-24 h (please record).

b) Only for patients who are not entering another clinical trial or receiving further anti-tumor treatment.

c) HAHA sampling on all cycles prior IV infusion. An additional sample for HAHA assessment as part of an unscheduled assessment will be taken from patients with clinical signs of immune complex-mediated toxicities.

d) Timing of sampling +3 h.

e) Timing of sampling within +24 h (please record).

f) Please note: Cycle 1 Day 1 for PK sampling is Day 2 in the Schedule of Assessments (Appendix 1).

g) **In case of quality of life-impairing Grade 2 diarrhea or Grade ≥3 diarrhea, PK samples should be taken at the beginning of the event, at the end of the event and at any clinical visit of the patient during the event.**

Appendix 2 Specific Schedule of Assessments for PK and HAHA (cont.)

b Pertuzumab and Paclitaxel Assessments on Cycle 1, End-of-study Assessments and Additional Assessments

Assessments	Day 1[f]						Day 2	Day 5	Day 9	Day 15	Day 28 (+/-3 days) post-infusion Fup visit/early-discontinuation visit	Day 42-45 post-infusion Fup visit[b]	IRR	Disease progression	DLT	≥Grade 2 diarrhea[g]
	Pre-dose	Post-infusion	Hours post-dose													
			1	2	4	6	24[e]	96[a]	192[a]	336[a]						
Pertuzumab PK samples	X	X			X		X	X	X	X	X	X	X	X	X	**X**
Paclitaxel PK samples	X	X	X	X	X	X	X	X								**X**
Pertuzumab HAHA	X[c]										X	X	X			

a) Timing of sampling within +/-24 h (please record) for pertuzumab only.
b) Only for patients who are not entering another clinical trial or receiving further anti-tumor treatment.
c) HAHA sampling on all cycles prior IV infusion. An additional sample for HAHA assessment as part of an unscheduled assessment will be taken from patients with clinical signs of immune complex-mediated toxicities.
d) Timing of sampling +3 h for pertuzumab only.
e) Timing of sampling within +24 h (please record) for pertuzumab only.
f) Please note: Cycle 1 Day 1 for PK sampling is Day 1 in the Schedule of Assessments (Appendix 1).
g) **In case of quality of life-impairing Grade 2 diarrhea or Grade ≥3 diarrhea, PK samples should be taken at the beginning of the event, at the end of the event and at any clinical visit of the patient during the event.**

Appendix 2 Specific Schedule of Assessments for PK and HAHA (cont.)

c Assessments on Cycle 4

Assessments	Day 1						Day 2	Day 4	Day 8	Day 12	Day 14	Day 19
	Pre-dose	Post-infusion	Hours post-dose									
			1	2	3	6	24[c]	72[a]	168[a]	264[a]	312[a]	432[a]
RO5479599 PK samples	X	X			X[b]		X	X	X	X	X	X
Pertuzumab PK samples[e]	X	X			X		X	X	X		X	
Paclitaxel PK samples[e]	X	X	X	X	X	X	X	X				
RO5479599 HAHA	X											
Pertuzumab HAHA	X											

a) Timing of sampling within +/-24 h (please record) for RO5479599 and pertuzumab only.
b) Timing of sampling +3 h for RO5479599 and pertuzumab only.
c) Timing of sampling within +24 h (please record) for RO5479599 and pertuzumab only.

d Assessments for All Other Cycles

Assessments	Day 1	
	Pre-dose	Post-infusion
RO5479599, pertuzumab and paclitaxel PK samples	X	X
RO5479599 HAHA	X	
Pertuzumab HAHA	X	

Appendix 3 NYHA Classification

Class I	No limitation of physical activity (asymptomatic)<br>Ordinary physical activity does not cause undue fatigue, palpitation or dyspnea
Class II	Slight limitation of physical activity<br>Comfortable at rest, but ordinary physical activity results in fatigue, palpitation or dyspnea
Class III	Marked limitation of physical activity<br>Comfortable at rest, but less than ordinary activity causes fatigue, palpitation or dyspnea
Class IV	Unable to carry on any physical activity without discomfort. Symptoms of cardiac insufficiency may be present even at rest. If any physical activity is undertaken, discomfort is increased

Appendix 4 Cockcroft-Gault Formula for Calculation of Creatinine Clearance

Creatinine CL (mL/min) for males:

$$\text{Creatinine CL} = \frac{([140 - \text{age <years>}] \text{ x body weight <kg>})}{72 \text{ x serum creatinine <mg/dL>}}$$

Creatinine CL (mL/min) for females:

$$\text{Creatinine CL} = \frac{([140 - \text{age <years>}] \text{ x body weight <kg>})}{72 \text{ x serum creatinine <mg/dL>}} \text{ x } 0.85$$

Appendix 5 Eastern Cooperative Oncology Group Performance Status Scale

Grade	Description
0	Fully active, able to carry on all pre-disease performance without restriction
1	Restricted in physically strenuous activity but ambulatory and able to carry out work of a light or sedentary nature, e.g., light housework or office work
2	Ambulatory and capable of all self-care but unable to carry out any work activities. Up and about >50% of waking hours
3	Capable of only limited self-care, confined to a bed or chair >50% of waking hours
4	Completely disabled. Cannot carry on any self-care. Totally confined to bed or chair
5	Dead

Appendix 6 ICH Guidelines for Clinical Safety Data Management, Definitions and Standards for Expedited Reporting, Topic E2

A serious adverse event (SAE) is any experience that suggests a significant hazard, contraindication, side effect or precaution. It is any Adverse Event (AE) that at any dose fulfills at least one of the following criteria:

- is fatal; (results in death)
  (NOTE: death is an outcome, not an event)
- is Life-Threatening
  (NOTE: the term "Life-Threatening" refers to an event in which the subject was at immediate risk of death at the time of the event; it does not refer to an event which could hypothetically have caused a death had it been more severe)
- required in-patient hospitalization or prolongation of existing hospitalization
- results in persistent or significant disability/incapacity
- is a congenital anomaly/birth defect
- is medically significant or requires intervention to prevent one or other of the outcomes listed above

Medical and scientific judgment should be exercised in deciding whether expedited reporting to the sponsor is appropriate in other situations, such as important medical events that may not be immediately life-threatening or result in death or hospitalization but may jeopardize the subject or may require intervention to prevent one of the outcomes listed in the definitions above. These situations should also usually be considered serious.

Examples of such events are intensive treatment in an emergency room or at home for allergic bronchospasm; blood dyscrasias or convulsions that do not result in hospitalization; or development of drug dependency or drug abuse.

An unexpected AE is one, the nature or severity of which is not consistent with the applicable product information.

Causality is initially assessed by the investigator. For SAEs, possible causes of the event are indicated by selecting one or more options. (Check all that apply)

- Pre-existing/Underlying disease - specify
- Study treatment – specify the drug(s) related to the event
- Other treatment (concomitant or previous) – specify
- Protocol-related procedure
- Other (e.g. accident, new or intercurrent illness) – specify

Appendix 6 ICH Guidelines for Clinical Safety Data Management, Definitions and Standards for Expedited Reporting, Topic E2 (cont.)

The term severe is a measure of intensity, thus a severe AE is not necessarily serious. For example, nausea of several hours' duration may be rated as severe, but may not be clinically serious.

An SAE occurring during the study or which comes to the attention of the investigator 15- 28 days after stopping the treatment or during the protocol-defined follow-up period (if this is longer), whether considered treatment-related or not, must be reported. In addition, an SAE that occurs after this time, if considered related to study drug, should be reported.

Such preliminary reports will be followed by detailed descriptions later which will include copies of hospital case reports, autopsy reports and other documents when requested and applicable.

For SAEs, the following must be assessed and recorded on the AE eform of the eCRF: intensity, relationship to study drug, action taken, and outcome to date.

The investigator must notify the Independent Ethics Committee/Institutional Review Board of an SAE in writing as soon as is practical and in accordance with international and local laws and regulations.

ROCHE CONTACT for SAEs:

See details on form entitled Protocol Administrative and Contact Information & List of Investigators Form [gcp_for00227] which is provided with this protocol.

STUDY MONITOR / CLINICAL PHARMACOLOGY STUDY MANAGER:

See details on form entitled Protocol Administrative and Contact Information & List of Investigators Form [gcp_for00227] which is provided with this protocol.

24 HOUR MEDICAL COVERAGE:

Identification of a contact for 24 Hour Medical Coverage is mandatory to be compliant with worldwide regulatory agencies and to ensure the safety of study patients.

An Emergency Medical Call Center Help Desk will access the Roche Medical Emergency List, escalate emergency medical calls, provide medical translation service (if necessary), connect the investigator with the Roche medical contact for this study and track all calls. The Emergency Medical Call Center Help Desk will be manned 24 hours 7 days a week. Toll free numbers will be distributed to all investigators running Roche Pharma Development clinical trials. The Help Desk will be used for medical emergencies outside regular business hours, or when the regular Clinical Science Leader/Clinical Pharmacology Leader cannot be reached.

See the attached *Protocol Administrative and Contact Information & List of Investigators form [gcp_for000227]*, for details of administrative, contact information, and Emergency Medical Call Center Help Desk toll-free numbers.
