# Supplementary material for: Direct estrogen receptor (ER) / HER family crosstalk mediating sensitivity to lumretuzumab and pertuzumab in ER+ breast cancer
Source: PLoS One. 2017 May 11;12(5):e0177331. doi: 10.1371/journal.pone.0177331 (PMC5426757; doi:10.1371/journal.pone.0177331)
Supplement: S1 Statistical Analyses — (DOCX) [file pone.0177331.s003.docx]

# Statistical Evaluation:

Outbred athymic (nu/nu) female mice (n=10 per treatment group) bearing ER+/HER2-low/HER3+ human breast cancer xenografts (HBCx-19) treated with vehicle control, single-agent lumretuzumab, single-agent pertuzumab, or combination therapy.

Primary tumor growth data were analyzed statistically using non-parametric methods due to the fact that the data showed asymmetrical behavior. Prior to this procedure, the data were baseline corrected with the tumor volume at treatment start, based on ratio.

Briefly, in a randomized two-sample design the treatment-to-control ratio:

and its two-sided parametric or non-parametric confidence interval (1-α) were calculated.

## Comparison of single-agent lumretuzumab and pertuzumab and combination (RO5479599 + pertuzumab) versus controls

(Figure 2a in main manuscript)

### Day 43

| **Group** | **% TGI compared to control** | **Tumor regression %** |
| --- | --- | --- |
| lumretuzumab 10 mg/kg | >100 | 33 |
| pertuzumab 30/15 mg/kg | >100 | 34 |
| lumretuzumab 10 mg/kg + pertuzumab 30/15 mg/kg | >100 | 76 |

TGI=Tumor Growth Inhibition

| **Group** | **Treatment** | **np TCR** | **np CI lower** | **np CI upper** |
| --- | --- | --- | --- | --- |
| 2 | lumretuzumab 10 mg/kg | 0.05 | 0.03 | 0.08 |
| 3 | pertuzumab 30/15 mg/kg | 0.05 | 0.02 | 0.06 |
| 4 | lumretuzumab 10 mg/kg + pertuzumab 30/15 mg/kg | 0.02 | 0.01 | 0.04 |

CI=confidence interval; np=non-parametric; TCR=Treatment-to-control Ratio

### Nonparametric TCR

CI=confidence interval; RO5479599=lumretuzumab; TCR=Treatment-to-control Ratio

All groups significantly different compared to control on Day 43.

## Comparison of single-agent lumretuzumab and pertuzumab versus combination (lumretuzumab + pertuzumab)

(Figure 2b in main manuscript)

### Day 61

| **Group** | **Tumor regression %** |
| --- | --- |
| lumretuzumab 10 mg/kg | 35 |
| pertuzumab 30/15 mg/kg | 47 |
| lumretuzumab 10 mg/kg + pertuzumab 30/15 mg/kg | 92 |

| **Group** | **Treatment** | **np TCR** | **np CI lower** | **np CI upper** |
| --- | --- | --- | --- | --- |
| 2 | lumretuzumab 10 mg/kg | 9.1 | 4.0 | 144 |
| 3 | pertuzumab 30/15 mg/kg | 7.9 | 3.3 | 136 |

CI=confidence interval; np=non-parametric; TCR=Treatment-to-control Ratio
Note: In this comparison, the two single-agent treatments are compared with combination treatment as the control; hence, a high TCR indicates worse tumor growth inhibition with single-agent therapies than with the control (combination treatment).

### Nonparametric TCR


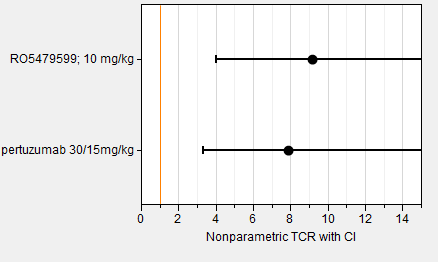


CI=confidence interval; RO5479599=lumretuzumab; TCR=Treatment-to-control Ratio

Combination treatment was significantly different compared to respective single agent arms.

### Day 92

| **Group** | **Tumor regression %** |
| --- | --- |
| lumretuzumab 10 mg/kg | - |
| pertuzumab 30/15mg/kg | - |
| lumretuzumab 10 mg/kg + pertuzumab 30/15mg/kg | 88 |

## Comparison of single-agent lumretuzumab and pertuzumab and combination (lumretuzumab + pertuzumab) versus controls

(Figure 4 in main manuscript)

### Day 41

| **Group** | **% TGI compared to control** |
| --- | --- |
| lumretuzumab 3 mg/kg | 86 |
| pertuzumab 3 mg/kg | 95 |
| fulvestrant 50 mg/kg | 36 |
| lumretuzumab 3 mg/kg + pertuzumab 3 mg/kg | 95 |
| lumretuzumab 3 mg/kg + fulvestrant 50 mg/kg | 89 |
| pertuzumab 3 mg/kg + fulvestrant 50 mg/kg | 94 |
| lumretuzumab 3 mg/kg + pertuzumab 3 mg/kg + fulvestrant 50 mg/kg | 99 |

TGI=Tumor growth inhibition

| **Group** | **Treatment** | **TCR** | **CI low** | **CI up** | **np TCR** | **np CI low** | **np CI up** |
| --- | --- | --- | --- | --- | --- | --- | --- |
| 2 | lumretuzumab 3 mg/kg | 0.21048 | 0.07143 | 0.35778 | 0.21696 | 0.12915 | 0.35986 |
| 3 | pertuzumab 3 mg/kg | 0.10458 | -0.0342 | 0.24746 | 0.09718 | 0.05934 | 0.16615 |
| 4 | fulvestrant 50 mg/kg | 0.64399 | 0.48957 | 0.82367 | 0.63636 | 0.35636 | 0.98904 |
| 5 | lumretuzumab 3 mg/kg + pertuzumab 3 mg/kg | 0.10441 | -0.0344 | 0.24728 | 0.10728 | 0.06063 | 0.16578 |
| 6 | lumretuzumab 3 mg/kg + fulvestrant 50 mg/kg | 0.19221 | 0.05332 | 0.33864 | 0.16143 | 0.1225 | 0.2887 |
| 7 | pertuzumab 3 mg/kg + fulvestrant 50 mg/kg | 0.11105 | -0.0277 | 0.25416 | 0.09925 | 0.04557 | 0.20623 |
| 8 | lumretuzumab 3 mg/kg + pertuzumab 3 mg/kg + fulvestrant 50 mg/kg | 0.05431 | -0.0849 | 0.19563 | 0.05934 | 0.01215 | 0.09495 |

CI=confidence interval; np=non-parametric; TCR=Treatment-to-control Ratio

### Parametric TCR

CI=confidence interval; RO5479599=lumretuzumab; TCR=Treatment-to-control Ratio

### Nonparametric TCR

CI=confidence interval; RO5479599=lumretuzumab; TCR=Treatment-to-control Ratio
